# Towards precision medicine strategies using plasma proteomic profiling for suspected gallbladder cancer: A pilot study

**DOI:** 10.1016/j.jhepr.2025.101365

**Published:** 2025-02-21

**Authors:** Ghada Nouairia, Martin Cornillet, Hannes Jansson, Annika Bergquist, Ernesto Sparrelid

**Affiliations:** 1 Division of Hepatology, Department of Medicine, Huddinge, Karolinska Institutet, Stockholm, Sweden; 2 Center for Infectious Medicine, Department of Medicine Huddinge, Karolinska Institutet, Stockholm, Sweden; 3 Division of Surgery and Oncology, Department of Clinical Science, Intervention and Technology, Karolinska Institutet, Stockholm, Sweden; 4 Department of Upper GI Disease Karolinska University Hospital, Stockholm, Sweden

**Keywords:** Cholecystitis, Tumor, Biomarkers, Machine learning

## Abstract

**Background & Aims:**

Currently, preoperative diagnostic methods that can distinguish cancer from benign disease of the gallbladder are insufficient, and several surgical resections can be avoided if the pathology is known prior to surgery. This study aimed to assess whether preoperative plasma proteins can distinguish gallbladder cancer (GBC) from cholecystitis, with the main goal of identifying proteins for multivariate description of the postoperative diagnosis, before surgery.

**Methods:**

Samples from 82 individuals with suspected GBC who underwent bisegmentectomy and lymphadenectomy at Karolinska University Hospital between 2009 and 2020 were included in this retrospective, observational, single-center study. Preoperative plasma samples were analyzed using a 7,500 proteomics panel from SomaScan®. High-dimensional statistical methods including machine learning regularization, were used to analyze the data.

**Results:**

In our study, we identified and characterized a panel of 651 proteins that exhibited differential expression between GBC and cholecystitis. Through multivariate analysis, we demonstrated that circulating proteomics data provide valuable insights for diagnosing GBC before surgical intervention. Notably, we identified a subset of eight plasma proteins (PAHX, CD8A, HRG, CRIS2, Dynactin subunit 2, AT2A3, CSTN2, and DEPP) that effectively differentiated GBC from cholecystitis with a diagnostic accuracy of 94% when validated on a test set. These findings hold potential for clinical validation and could significantly aid in preoperative decision-making when GBC is suspected.

**Conclusions:**

Our findings demonstrate that the preoperative assessment of plasma proteins can accurately differentiate cholecystitis from malignancy, supporting the potential development of a noninvasive test to assist preoperative decision-making when GBC is suspected.

**Impact and implications:**

This study highlights the potential of plasma proteomic profiling to significantly improve the preoperative diagnostic accuracy of gallbladder cancer *vs.* cholecystitis. Using machine learning models, we identified biologically relevant plasma proteins associated with the diagnosis of gall bladder cancer. A noninvasive preoperative test based on selected plasma proteins could potentially enhance clinical decision-making, reduce unnecessary surgeries, and mitigate the associated risks for patients with suspected GBC, marking a step forward in precision medicine.

## Highlights

• The plasma proteome can differentiate gallbladder cancer from cholecystitis with high accuracy.
• Machine learning models can identify a biologically relevant subset of differentiating plasma proteins.
• Plasma protein tests could have the potential to assist in preoperative decision-making when GBC is suspected.

## Graphical abstract

**Figure:**
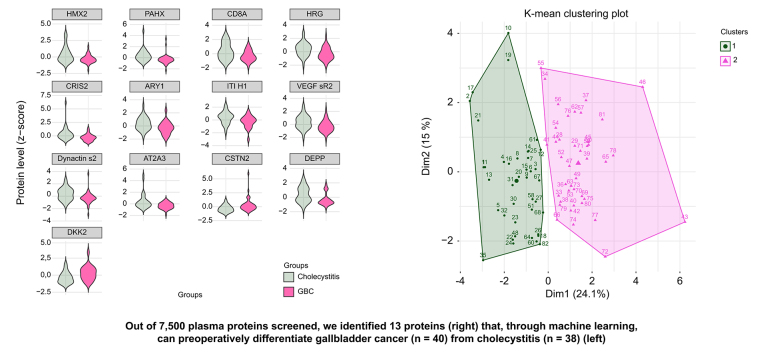

## Introduction

**Fig. 1:**
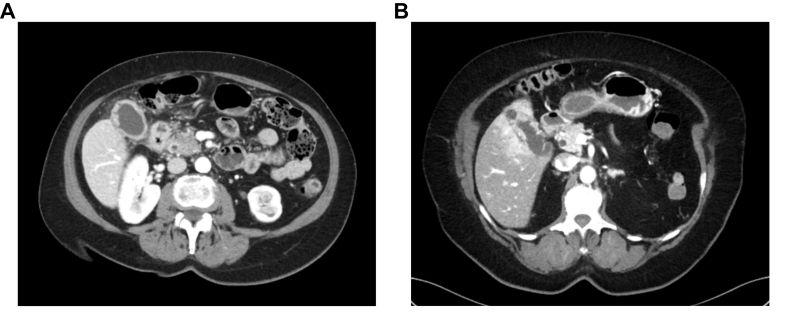
Demonstrating the preoperative diagnostic challenge in gallbladder cancer (GBC). (A) Imaging of a patient with GBC with primary suspicion of cholecystitis on preoperative investigation. (B) Imaging of a patient with cholecystitis with primary suspicion of malignant disease on preoperative investigation.

Gallbladder cancer (GBC) and inflammatory disease of the gallbladder (cholecystitis) are clinical entities that are difficult to separate before histological analysis, especially xanthogranulomatous cholecystitis.^1^^,^^2^ Despite advances in modern radiology, to date, no diagnostic modality has been sufficiently accurate^3^ and differential diagnosis is challenging (Fig. 1).

Biopsies should be avoided preoperatively because of the risk of tumor seeding and spread. Hence, many individuals with radiological suspicion of GBC undergo radical cholecystectomy and lymphadenectomy even if the diagnosis is often uncertain. Radical cholecystectomy generally consists of resection of liver segments 4b and 5 together with gallbladder or wedge resection of the adjacent liver parenchyma surrounding the gallbladder.^4^

When postoperative histology revealed only inflammation in the gallbladder, these patients received an unnecessary invasive treatment, with a substantial risk of complications. If accurate methods to distinguish between cancer and inflammation of the gallbladder before surgery are available, simple cholecystectomy with much lower risks, or even no surgery, could have been preferred for these individuals. Several attempts have been made to improve preoperative diagnostics in this patient group without convincing results.[5], [6], [7] To date, clinicians have been faced with the risk of overtreating some patients to avoid missing the optimal treatment window for individuals with early GBC, where delayed diagnosis leads to poorer prognosis.

The use of plasma biomarkers for diagnosis is gaining interest.[8], [9], [10] In 2014, Wang *et al.*^8^ evaluated the serum levels of CA 242, CA 125, CEA, and CA 19-9, concluding that CA 242 and CA 19-9 might assist in the diagnosis in the early stage and as prognostic markers for GBC compared with benign gallbladder disease.^8^ Recently, in 2023, Baichan *et al.*^9^ analyzed tissue and blood using liquid chromatography-mass spectrometry and identified nine dysregulated proteins (APOA1, APOA2, RET4, TTR, HEMO, HBB, HBA, PIGR, and APOE) in both tissue and plasma compared with benign biliary pathology. Several studies have evaluated GBC protein biomarkers at different tumor stages,^9^^,^^11^ including extracellular vesicles^12^ and plasma proteins. In 2021, a study identified 29 proteins from plasma-derived extracellular vesicles that were uniquely dysregulated in early stage GBC and clinically verified three proteins (NT5E, ANPEP, and MME).^12^ Tissue^13^^,^^14^ and plasma-derived extracellular vesicles^12^ have been identified as potential biomarkers for GBC stages. It is noteworthy that, to our knowledge, no GBC biomarker discovery study has screened more than 2,600 proteins.^10^ These previous studies relied on statistical tests to compare GBC and control groups. The use of such frequency-based analyses can lead to high false-positive rates owing to multiple testing.^16^ They only show associations between the disease and the biomarker, and cannot necessarily be translated to predictive potential.^17^ Bayesian model-based machine learning (ML) methods can improve biomarker discovery in large datasets as follows: (1) they are fitted to high-dimensional data, particularly when the number of variables outweighs observations and (2) they optimize biomarker selection based on outcome predictability (*i.e.* GBC *vs.* cholecystitis).[18], [19], [20]

In this study, we aimed to investigate whether preoperative assessment of plasma proteins in samples can distinguish GBC from cholecystitis and to explore whether proteomics could be used in the future to predict cancer preoperatively.

## Materials and methods

This retrospective single-center study was approved by the Swedish Ethical Review Authority (Dnr 2013/188-31, 2014/2118-32, 2020-02702) and was performed in accordance with the Strengthening the Reporting of Observational Studies in Epidemiology guidelines.^21^

### Study population and design

Adults (≥18 years of age) who underwent bisegmentectomy or wedge resection (anatomical resection of liver segments 4b and 5 or part of these segments) with lymphadenectomy and frozen sections of the cystic duct for suspected GBC at Karolinska University Hospital (2009–2020) were screened for inclusion in the study.

Based on the pathology report after resection, patients were divided into two groups: inflammation (cholecystitis) and GBC. Individuals with advanced tumors requiring more extensive resection, immunosuppressive treatment, surgery for incidental GBC from a previous cholecystectomy, or diagnoses other than GBC or cholecystitis were excluded. After screening, two groups of similar sizes were obtained.

Clinical data of patients from the cholecystitis (n = 38) and GBC (n = 44) groups and their correlation with proteomics data were explored using a computational technique called singular value decomposition (SVD) analysis. In SVD, principal component analysis (PCA) was first performed to reduce data dimensionality, and then the correlation of clinical parameters with the principal components was statistically tested (see Fig. S1A).

### Surgical management

During the study period, all patients with suspected GBC were discussed at a multidisciplinary team conference where the decision to recommend surgery and the operating strategy were taken. Individuals with a preoperative suspicion of GBC were operated (2009–2020) and divided into two groups, cholecystectomy and GBC, based on the histology of the operative specimen. All patients had a frozen section from the cystic duct, and if positive for cancer or high-grade dysplasia, resection of the extrahepatic bile duct was performed with hepaticojejunostomy. All patients had at least one regional lymphadenectomy extending to stations 8a, 12a, b, c, and p.

### Proteomics screening

All patients who underwent surgery at Karolinska University Hospital since 2019 were asked to provide written consent for inclusion in the prospective biobank consisting of all hepatobiliary surgeries. Preoperative EDTA plasma samples were collected on the day of surgery, centrifuged, aliquoted, frozen, and stored at -80 °C. Plasma samples acquired from patients preoperatively were analyzed using an aptamer-based proteomics platform, SomaScan®^22^ (SomaLogic, Boulder, CO, USA), using a 7 K assay. This novel technology ensures specific binding to different proteins and enables their accurate quantification. All raw data are publicly available at https://doi.org/10.6084/m9.figshare.26388166. Additional details are provided in the Supplementary CTAT Table.

### Statistical and computational analyses

A total of 80 patients was sufficient to reach an acceptable power of 80% (medium effect size and a significance level of 0.05), with a significance level of 0.05. All statistical analyses were performed using R version 4.3.1 (2023-06-16, R Foundation for Statistical Computing, Vienna, Austria). The data, in the ‘.adat’ format were loaded and manipulated using the SomaDataIO package.^7^ The quality of the data was controlled and then centered and scaled for downstream analysis. Proteins from organisms other than humans and duplicate isotopes were excluded from analysis.

### ML methods

**Fig. 2:**
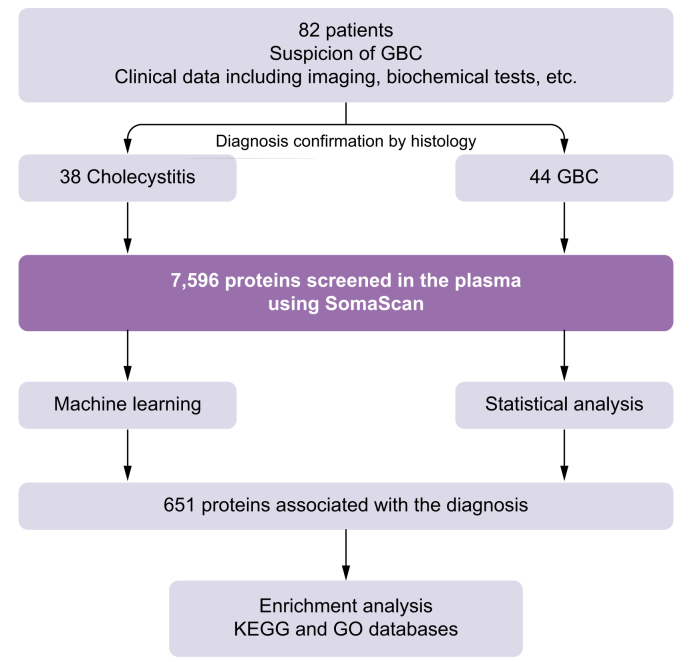
Flow chart of the study design and methods used. GBC, gallbladder cancer; GO, Gene Ontology; KEGG, Kyoto Encyclopedia of Genes and Genomes.

The elastic net (EN) regularization method, based on generalized linear models, was used as a feature (variable) selection method using the glmnet v4.1.8 package in R and the caret package v6.0-94. Data were split into training and test sets (80% to 20%) with respect to the GBC and cholecystitis groups, and we used the leave-one-out cross-validation technique. The models were run iteratively with different hyperparameters (alpha and lambda) to determine the lambda parameter with the highest model performance, best fit, and lowest complexity for a given alpha. Setting alpha defines the regularization method that is used. The least absolute shrinkage and selection operator (LASSO) regression (alpha = 1) defines a stringent cut-off of less informative variables, whereas a strictly positive alpha that is <1 implements an EN regression where collinear variables are maintained in the model (Fig. 2). Model performance was evaluated using the root mean square deviation (RMSE), mean square deviation (MSD), correlation (R^2^), receiver-operating characteristic curve (ROC), and area under the ROC curve (AUC) metrics.

### Association analysis and clustering

We calculated the standardized mean differences and the 95% CIs for each protein using an independent *t* test to compare the mean levels between the cholecystitis and GBC groups. Dimensionality reduction (PCA) was used to visualize the data. The clustering potential of plasma proteins identified by ML was tested using unsupervised clustering methods (k-means clustering and hierarchical clustering).

### Enrichment analysis

Using all the proteins found by the EN methods and the statistical significance test, we performed enrichment analysis, using Kyoto Encyclopedia of Genes and Genomes (KEGG) pathway (https://www.genome.jp/kegg/pathway.html) and Gene Ontology (GO, https://geneontology.org/) databases, clusterProfiler v4.8.3 R package, Enrichr,^23^ and ShinyGO web-tool.^24^ All scripts used in computational analysis are available at GitHub (https://github.com/MedH-AB-group/Cancer_plasma_proteomics).

## Results

Overall, 82 patients with clinical and radiological suspicion of GBC were included in this study. Of these, 44 patients had GBC in the pathology report and 38 had cholecystitis. The clinicopathological characteristics are shown in Table 1.

**Table 1:** General description of clinical characteristics of the cohort.

	Cholecystitis n = 38	GBC n = 44	*p* value (Χ^2^)
**General description**			
Age, median (IQR)	63.5 (18.2)	69 (12.5)	0.036
Sex, n (%)			
Female	16 (42)	26 (59)	
Male	22 (58)	18 (41)	
BMI, median (IQR)	27 (8)	25.9 (5.7)	0.241
Diabetes, n (%)	7 (18)	4 (9)	
Immunosuppressive medication, n (%)	0	0	
Primary sclerosing cholangitis (PSC), n (%)	0	7 (16)	
Cirrhosis, n (%)	0	1 (2)	

**Baseline tests**			
CRP, median (IQR)	2 (4.5)	2 (5.8)	0.227
Albumin, median (IQR)	36 (4)	33 (6)	0.009
Bilirubin, median (IQR)	6 (4)	6 (6)	0.616
CA199, median (IQR)	15 (12)	110 (110.5)	0.076

**Imaging**			
CT, n (%)	34 (89)	39 (88)	
MRI, n (%)	17 (45)	17 (39)	
Both CT and MRI, n (%)	13 (34)	13 (29)	
Preoperative biliary stenting, n (%)	1 (2)	4 (9)	

**Pathological report**			
Inflammation	All	–	
T stage, n (%)	–		
*In situ*		1 (3)	
1		3 (9)	
2		17 (53)	
3		11 (34)	
4		0	
Lymph node status, n (%)	–		
N0 (0 positive nodes)		19 (43)	
N1 (1–3 positive nodes)		11 (25)	
N2 (≥4 positive nodes)		2 (4)	
NA		12 (27)	
M status, n (%)	–		
M0		42 (95)	
M1 (aortocaval nodes)		2 (5)	

*p* values are calculated based on the Χ^2^ test. CRP, C-reactive protein; GBC, gallbladder cancer; n, number of occurrences; n, number of patients.

Before surgery, radiological investigation with either computed tomography (CT) scan (57%), magnetic resonance imaging (MRI) (10%), or both (32%) was performed. The use of preoperative radiology was similar in patients with confirmed GBC and those with a postoperative cholecystitis diagnosis.

We assessed the relationships between clinical factors and proteomics profile using SVD analysis (Fig. S1A). Liver cirrhosis and primary sclerosing cholangitis did not show any significant associations, whereas diabetes displayed correlation (significant at *p* <0.05) with ∼5% of the proteomic data (Table 1 and Fig. S1A).

### Large-scale plasma proteomic signature distinguishes cholecystitis from GBC

**Fig. 3:**
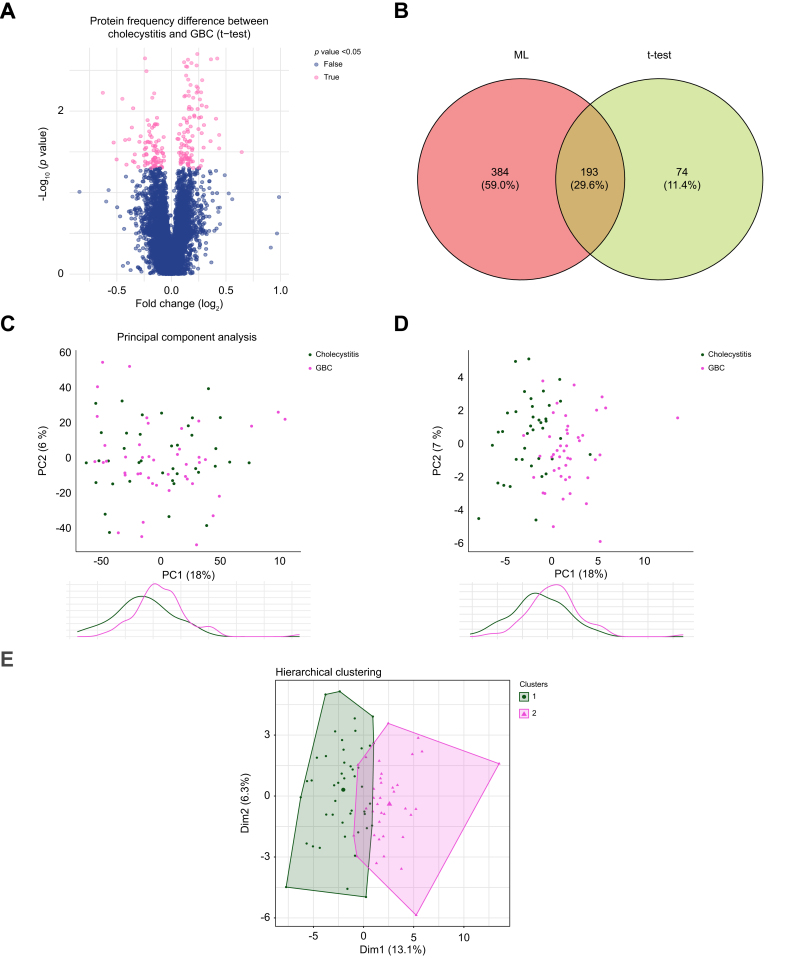
A set of 651 proteins were associated with diagnosis. (A). Volcano plot of all the proteins (in blue) and those significantly (*p* <0.05) linked to the diagnosis (in pink), in *t* test. (B) Venn diagram of the overlap between results from elastic net (n = 577), and *t* test at *p* <0.05 (n = 267). (C) Principal component analysis (PCA) of the whole dataset (7,500 proteins) with no apparent data structure. (D) PCA using the 651 diagnosis-associated proteins showed separation of GBC and cholecystitis. (E) Unsupervised hierarchical clustering of diagnosis-associated proteins largely distinguished GBC and cholecystitis, yet in the central region these groups overlapped (11% of the patients). Dim, dimension; GBC, gallbladder cancer; PC, principal component.

The primary objective was to determine whether circulating proteins detected preoperatively in the peripheral blood can accurately distinguishing between patient groups (malignancy or cholecystitis). We identified 267 proteins in the plasma that were differentially expressed between the groups (Fig. 3A), using statistical tests. Notably, three of these proteins exhibited the highest standard mean difference (SMD >0.73) in this study, indicating prominent group differences: the cysteine-rich secretory protein 2 (CRIS2) with a raw mean difference (MD) of 381 (95% CI 142–619), the homeobox protein HMX2 (MD 70.7, 95% CI 26–115), and the sarcoplasmic endoplasmic reticulum calcium ATPase 3 (AT2A3) (MD 143, 95% CI 51–235). Given the large number of proteins tested (7,500) relative to the modest sample size (n = 82), we recognize the importance of addressing the multiple testing issue to avoid false positives.

Given the high dimensionality of the data, we opted for the regularization method EN, an ML method that (1) can handle high-dimensional datasets, (2) prevents overfitting by utilizing Bayesian statistics, and (3) balances between retaining correlated variables and performing variable selection.^25^ Unlike traditional methods, which may exhibit multicollinearity, EN can identify proteins that contribute jointly to the model even if they are highly correlated. To explore the multivariate association within the proteomic data, we applied an EN-regularized regression. This ML model incorporated a subset of 577 proteins associated with postoperative diagnosis. The EN model achieved a high performance on the test set, with an AUC of 94%. The model’s mean squared error and the root mean squared error were low, at 0.01 and 0.12, respectively, while the R^2^ metric was 93%, indicating a strong fit to the data. Collectively, these measurements underscored the capacity of this subset of proteins to differentiate between the GBC and cholecystitis profiles, supporting the robustness of our multivariate approach.

Notably, 193 proteins (29.6%) identified through EN also appeared in the statistical *t* test results (Fig. 3B), exhibiting a medium to large effect SMD ranging between 0.4 and 0.75. This overlap underscores the significance of these proteins, even when considering the limitations of a small sample size and the burden of multiple testing.

Combining the results from the frequentist statistical analysis and the ML results, we identified diagnosis-associated proteins (651) affected by both upregulation (316 proteins) and downregulation (341 proteins). In the PCA performed using the whole proteomic dataset (7,500 proteins), no data pattern was observed (Fig. 3C). However, using the proteins identified by the EN and *t* test, amounting to 651 proteins, revealed a strong differentiation of GBC and cholecystitis (Fig. 3D and E).

### ML methods identify a minimal plasma protein signature for preoperative diagnosis of GBC

**Fig. 4:**
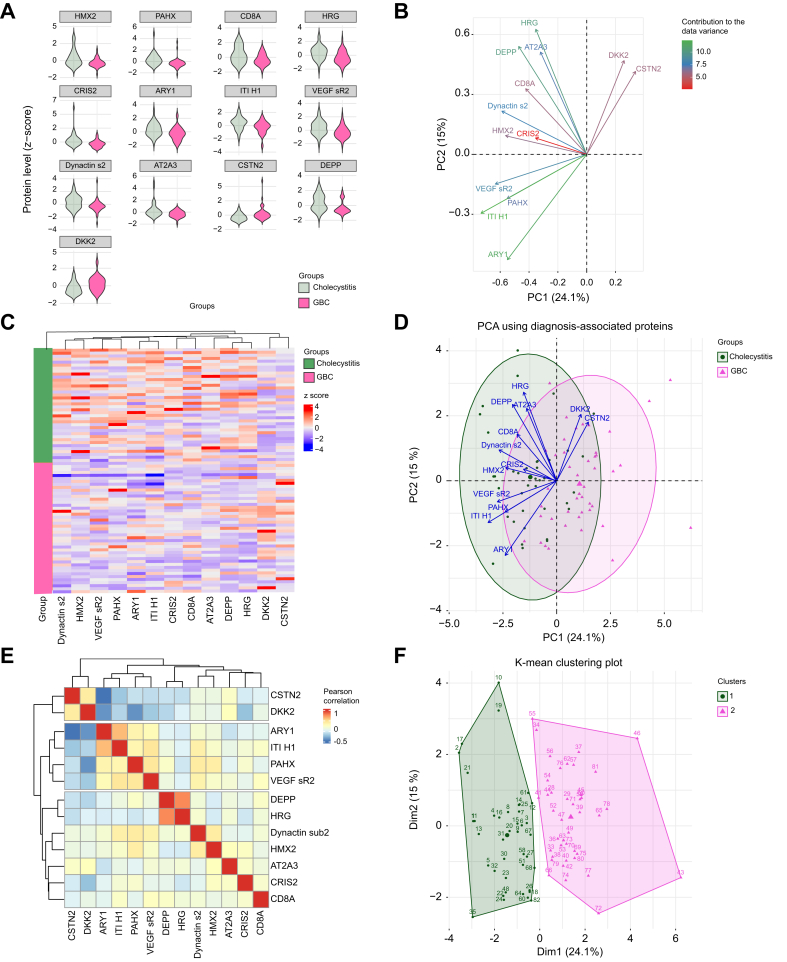
Characteristics of the most descriptive proteins of postoperative diagnosis. (A) The variance of the protein levels in patients with cholecystitis and GBC. (B) Direction and contribution of each protein to the data variance, assessed by principal component analysis (PCA). (C) Heatmap showing the difference of expression of the proteins between the two groups. (D) Data variance and group differences are well described by the proteins, visualized by PCA. (E) Low correlation between the proteins confirming non-redundant information in the ML model. (F) Thirteen proteins could separate the patients into two clusters (k-mean unsupervised clustering) that largely correspond to the cholecystitis and GBC groups (89% of the patients were rightfully assigned). All nine wrongfully assigned patients were in the middle area. PCA, principal component analysis.

By adjusting the alpha parameter in our ML model and performing LASSO regression, we identified a more parsimonious set of 13 proteins (Fig. 4) associated with the postoperative diagnosis. This refined model achieved an AUC of 98% in the test set, suggesting a strong discriminatory capacity for these proteins within the cohort. Although not aimed at predictive applications, the high AUC underscores the potential of these selected proteins to capture key multivariate relationships distinguishing GBC from cholecystitis profiles. Interestingly, 12 of these proteins had the highest SMD in this study, ranging from 0.64 to 0.74. The exception was dickkopf-related protein 2 (DKK2), which had a medium SMD of 0.3. This streamlined protein set further reinforces the robustness of our multivariate approach and offers insight into potentially influential biomarkers.

Next, we further explored the proteins selected by the ML model in relation to the dataset. Individually, no protein showed a distinct frequency difference between GBC and cholecystitis (Fig. 4A and C). However, PCA demonstrated that the proteins largely accounted for data variability and spanned their directions (Fig. 4B and D). K-means clustering, using the 13 same proteins, resulted in an unsupervised bifurcation of the patients into two clusters (*i.e.* groups), largely aligning with the cholecystitis and GBC groups (Fig. 4F). Eleven percent of the patients were misclassified and all fell within the intermediate area. K-means clustering optimizes grouping by minimizing intragroup distances and maximizing intergroup distances. However, for data points in the intermediate area, patients were not accurately assigned to their respective groups. In fact, upon iteration with different cluster quantities (changing the K value), the optimal cluster count was three, where patients in the middle constituted a distinct group.

### Pathway enrichment analysis reveals biological mechanisms underlining cholecystitis and GBC

**Fig. 5:**
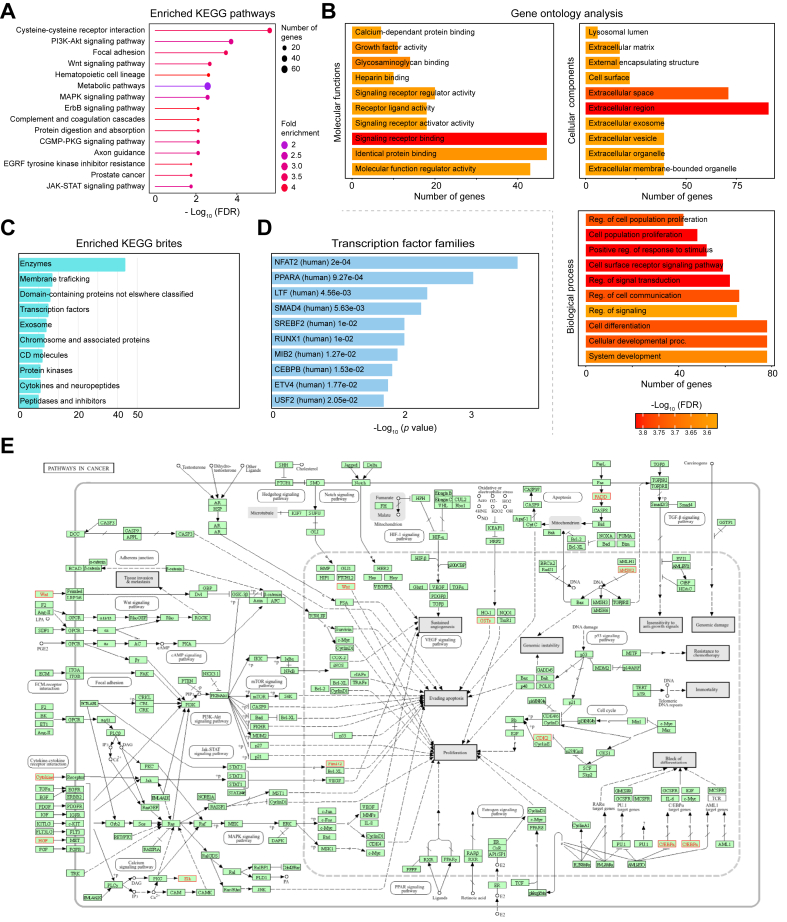
Enrichment analysis of the proteins linked to diagnosis. (A) Enriched KEGG pathways in the diagnosis-associated proteins (651) where cytokine receptors are the most significant. (B) Gene Ontology analysis of the molecular functions, the cellular components, and the biological processes of the diagnosis-associated proteins. (C) Enriched KEGG brites in diagnosis-associated proteins. (D) Transcription factor families enriched in the protein list. (E) Cancer pathways in KEGG database with dysregulated proteins (from this study) highlighted in red. FDR, false discovery rate; KEGG, Kyoto Encyclopedia of Genes and Genomes. N. of genes, number of genes. *p* values are calculated based on Fisher's exact test.

To gain insight into the molecular functions and biological pathways overrepresented in these 651 diagnosis-associated proteins, we performed an enrichment analysis. Cytokine receptor binding as well as activity proteins and the pro-inflammatory cytokine pathway (TNF signaling pathway) were significantly (adjusted *p* <0.01) enriched both in the proteins’ molecular functions and the biological pathways in which they were involved (Fig. 5A and B), as expected in the presence of inflammation. The set was also enriched (adjusted *p* <0.01) in proteins related to growth development (serine activity and growth factor binding), membrane receptors (transmembrane receptors and G protein binding), and glycosaminoglycan-binding functions. Based on KEGG brites (Fig. 5C), several diagnosis-associated proteins belonged to membrane trafficking, chromosome-associated proteins, and transcription factors. Notably, four transcription factor families were significantly enriched in diagnosis-associated proteins (*p* <0.01) (Fig. 5D and Table S2). Finally, we connected HGF, CDK2, FADD, and other proteins to the cancer pathways in the KEGG database (Fig. 5E).

## Discussion

In this retrospective exploratory study, we investigated the potential of the plasma proteome to preoperatively differentiate between GBC and cholecystitis. We aimed to identify a minimum set of proteins that could reflect the postoperative diagnosis before surgery. Plasma samples from 82 individuals who underwent liver resection were analyzed. Using ML techniques, protein subsets could differentiate patients with a postoperative histological diagnosis of GBC from patients with cholecystitis with 98% accuracy. Indeed, reliable clustering is not feasible for a minor subset of patients. This study, although exploratory and lacking external validation, used a reasonable patient pool, screened a large number of proteins (7,500 proteins), and used cutting-edge and suitable computational techniques. Our findings suggest that the preoperative measurement of plasma proteins could be informative regarding the presence of GBC. Preoperative blood tests could potentially be developed to enhance diagnostic accuracy, particularly in individuals with pre-existing inflammatory conditions.

The SomaScan® technology from SomaLogic is a pioneering high-throughput proteomics technology that allows data-driven biomarker discovery. Modified aptamers are called SOMAmers and are small DNA sequences with high-affinity target-specific tertiary structures. Their slow off-rate binding kinetics were measured using microarray-based fluorescence intensity readout.^22^ This affinity-based approach overcomes the challenge of detecting proteins at vastly different concentrations, known as the dynamic range issue, and outperforms mass spectrometry.^26^ Our choice of ML regularization method was motivated by the high dimensionality of the data. EN and LASSO are robust variable (proteins in this case) selection methods that integrate ML techniques, such as cross-validation and training, to optimize the model for outcome predictability (diagnosis in this case). In addition, approximately one-third of the proteins identified by EN overlapped with the statistical *t* test results, reinforcing the relevance of these proteins despite the challenges posed by the limited sample size and multiple testing. This overlap suggests that although individual statistical significance may not have survived strict multiple testing correction, the proteins identified by both methods likely reflect biologically meaningful associations with the disease. Together, these analyses highlight the importance of considering complementary approaches, such as EN in large proteomic studies where traditional statistical methods may be overly conservative.

Among the identified diagnosis-associated proteins, several hold promise for clinical application. For example, CD8 chain (T-cell surface glycoprotein CD8 alpha chain [CD8A]), HMX2, histidine-rich glycoprotein (HRG), ATPase Sarcoplasmic/Endoplasmic Reticulum Ca2+ Transporting 3 (AT2A3), and the decidual protein induced by progesterone (DEPP)^27^ can be tested using commercially available ELISA or Western blot kits. In addition, some of these proteins are associated with oncogenesis and progression of GBC. Arylamine N-acetyltransferase 1 (ARY1) is a key enzyme in various cancers, including GBC.^28^^,^^29^ It is upregulated in several cancer types and can be a target for cancer therapy and patient stratification.^30^ Vascular endothelial growth factor receptor 2 (VEGFR2) is a well-known target in cancer therapy because of its role in tumor induced angiogenesis.^31^^,^^32^ Histidine-rich glycoprotein (HRG) has been detected in plasma and has been suggested as a potential prognostic and diagnostic biomarker in cancer.^33^^,^^34^ Finally, CD8A, a component of the CD8 co-receptor complex, has been linked to GBC subtypes and prognosis.^35^ In contrast, diagnosis-associated proteins include several transcription factors, including HNF4A and PRDM1, which were previously associated with cholangiocarcinoma.^36^^,^^37^ The NFAT2 family, the most significantly enriched (*p* = 0.0001) family in our results, has been linked to the progression of bladder urothelial carcinoma^38^ and various human solid tumors and hematologic malignancies.^39^ Our results highlight the potential relevance of these proteins to GBC pathobiology.

However, knowledge regarding the etiology of GBC is limited. A history of gallstones and cholecystitis is the strongest known risk factor.^40^ Other factors include advanced age, female sex,^41^ chronic bacterial infections (*Salmonella* and *Helicobacter*),^40^ and primary sclerosing cholangitis.^42^ The etiology and related biochemical alterations in blood during GBC development remain largely unknown. Here, we describe a set of proteins that may shed light on the pathogenesis of GBC. Several proteins identified in our analysis are implicated in the pathophysiology of GBC and other biliary tract cancers, suggesting few pathways that are critical to tumor progression and immune evasion. For example, phytanoyl-CoA dioxygenase (PAHX) is involved in peroxisomal metabolism,^43^ and its alterations may disrupt lipid metabolic pathways, which have been increasingly linked to tumorigenesis in biliary tract cancers.^44^ The DEPP protein, known for its role in oxidative stress responses,^45^ may contribute to the heightened oxidative environment that facilitates cancer progression.^46^ CRISP2 is a member of the cysteine-rich secretory protein family and has been linked to immunomodulation and pathophysiology of chronic pancreatitis.^47^ This protein was likely linked to the diagnosis of cholecystitis in our study. VEGF sR2, a soluble form of VEGF receptor, is crucial for vascular endothelial growth in biliary pathophysiology and angiogenesis regulation. Its dysregulation is a known factor in biliary tract cancers, where angiogenesis supports tumor growth and metastasis.^48^^,^^49^ HRG and DKK2 are involved in immune modulation and Wnt signaling,^50^ respectively. Both pathways play essential roles in cancer progression and anticancer immune response.^51^^,^^52^ Additionally, CD8A, a protective gene with the highest correlation with T cells, is a promising prognostic biomarker in bladder cancer,^53^ calsyntenin-2 (CSTN2) has been identified as a potential biomarker for colorectal cancer,^54^ and dynactin subunit 2 has been identified as an oncogene in hepatocellular carcinoma.^55^

Notably, we found a significant association between serpin family A (SERPINA1), Annexin A protein 3 (ANXA3), collagen type VI alpha 1 chain (COL6A1), proteinase 3 (PRTN3), and keratin (KRT18) with GBC diagnosis, through statistical tests and ML techniques. These proteins have been previously associated with early stage GBC.^13^^,^^56^ Additionally, KRT1, ANXA2, peptidyl-prolyl cis-trans isomerase B (PPIB), colony stimulating factor 1 (CSF1), and receptor tyrosine-protein kinase (ERBB3) were identified using ML methods in this study. The first three proteins were recently detected in tissue samples and were associated with lymph node metastasis in GBC,^14^ whereas ERBB3 has been described as a targeted therapy for GBC.^59^ Recently, CSF1 was described as a potential preoperative plasma marker for biliary tract cancer.^60^

Collectively, these proteins provide insights into the multifaceted biological mechanisms driving the development of GBC and other bile duct cancers and may serve as promising future plasma biomarkers for preoperative GBC diagnosis. Our findings complement those of previous studies, in which plasma biomarkers have been reported for the diagnosis and prognosis of GBC.^8^ In a study by Wang *et al.*^8^, a combination of plasma markers (CA125, CA242, and CA199) enhanced the diagnostic sensitivity of GBC compared with individual markers. Notably, our extensive protein panel did not include CA19-9 or CA242, and we found a low SMD for CA125 in our cohort. Furthermore, carcinoembryonic antigen (CEA) exhibited an SMD of 0.2, suggesting a limited role as a diagnostic marker in our analysis. This highlights the need for further investigation of alternative biomarkers that may provide improved diagnostic capabilities for GBC.

The molecular functions and biological pathways identified in our study are related to cancer factors and infections. Signal transduction and signaling molecules and interactions, such as cytokine receptor-related pathways and the TNF signaling pathway, were significantly enriched. These pathways are characteristic of inflammation as seen in cholecystitis.^61^ The MAPK, JAK-STAT, and PI3K/AKR pathways, which were significantly dysregulated in our study, are associated with inflammation, cholecystitis, and tumor progression.^62^^,^^63^ ErbB and the coagulation and complement cascade pathways were associated with thromboinflammatory process regulation in tumor progression^2^ and were enriched in downregulated proteins in our analysis. The axon guidance pathway, which is altered in pancreatic ductal adenocarcinoma,^1^ was enriched in this study. Lastly, the Wnt signaling pathway was significantly enriched with upregulated proteins, including DDK2, which was highly associated with GBC diagnosis in our study. This pathway promotes colon carcinogenesis.^64^

This study had several limitations. Because of the rarity of GBC, a relatively small number of samples were studied. However, this was considered when choosing analysis methods that were adapted to such conditions. Nevertheless, the results are exploratory and further validation of the present results is required. A validation step is feasible and cost-effective because of the low number of proteins, their availability for clinical testing, and their detection at the plasma level. Importantly, validation efforts would require a larger collection of samples and, potentially, multicenter and international collaboration.

In conclusion, to our knowledge, our study provides the largest proteomic characterization of GBC that can serve as a resource for further biomarker discovery. We show that preoperative assessment of plasma proteins can accurately differentiate cholecystitis from malignancy, supporting the development of a noninvasive test to assist preoperative decision-making when GBC is suspected.

## Abbreviations

ANXA, annexin A; ARY1, arylamine N-acetyltransferase 1; AT2A3, sarcoplasmic/endoplasmic reticulum calcium ATPase 3; AUC, area under the ROC curve; CD8A, T-cell surface glycoprotein CD8 alpha chain; CEA, carcinoembryonic antigen; COL6A1, collagen type VI alpha 1 chain; CRIS2, cysteine-rich secretory protein 2; CSF1, colony stimulating factor 1; CSTN2, calsyntenin-2; CT, computed tomography; DEPP, decidual protein induced by progesterone; DKK2, dickkopf-related protein 2; dynactin sub2, dynactin subunit 2; EGRF, epidermal growth factor receptor; EN, elastic net; ERBrbB3, receptor tyrosine-protein kinase erbB-3; FDR, false discovery rate; GBC, gallbladder cancer; GO, Gene Ontology; HMX2, homeobox protein HMX2; HRG, histidine-rich glycoprotein; Is, *in situ*; ITI H1, inter-alpha-trypsin inhibitor heavy chain H1; KEGG, Kyoto Encyclopedia of Genes and Genomes; KRT, keratin protein; LASSO, least absolute shrinkage and selection operator; MD, mean difference; ML, machine learning; MRI, magnetic resonance imaging; MSD, mean square deviation; PAHX, phytanoyl-CoA dioxygenase peroxisomal; PC, principal component; PCA, principal component analysis; PPIB, peptidyl-prolyl cis-trans isomerase B; PRTN, proteinase; RMSE, root mean square deviation; ROC, receiver operating characteristic curve; SERPINA, serpin family A; SMD, standard mean difference; SVD, singular value decomposition; TNF, tumor necrosis factor; VEGF sR2, soluble vascular endothelial growth factor receptor 2.

## Financial support

This study was supported by grants from the 10.13039/501100003748Swedish Society for Medical Research (10.13039/501100003748SSMF), the 10.13039/501100018713Center for Innovative Medicine at 10.13039/501100004047Karolinska Institutet and RegionStockholm. The funding sources were not involved in the design or conduct of the research, nor the analysis and interpretation of the data, or the writing of this manuscript and decision to submit the article for publication to this journal.

## Authors’ contributions

Contributed to the study concept and design: MC, AB, ES. Contributed to data acquisition: MC, AA, HJ. Performed the bioinformatics and computational analyses: GN. Interpreted data and drafted the manuscript: GN, MC, AB, ES. Critically revised and approved the final version of the manuscript: all authors.

## Data availability statement

Raw data is available on https://doi.org/10.6084/m9.figshare.26388166. Public access to the data is restricted by the Swedish laws and regulations that prohibit the release of individual-level datasets that could potentially allow a personal identification. In this study, this particularly concerns some of the clinical metadata and patients’ s characteristics. However, data access can be granted in the framework of a defined academic research collaboration with requirement of data transfers agreement. Anyone wishing to gain access to the data should contact Martin Cornillet and Ernesto Sparrelid (martin.cornillet.jeannin@ki.se, ernesto.sparrelid@ki.se).

## Conflicts of interest

The authors declare no conflicts of interest that pertain to this work.

Please refer to the accompanying ICMJE disclosure forms for further details.
