# Supplementary material for: Towards precision medicine strategies using plasma proteomic profiling for suspected gallbladder cancer: A pilot study
Source: JHEP Rep. 2025 Feb 21;7(6):101365. doi: 10.1016/j.jhepr.2025.101365 (PMC12135361; doi:10.1016/j.jhepr.2025.101365)
Supplement: Multimedia component 1 [file mmc1.pdf]

Towards precision medicine strategies using plasma proteomic profiling for suspected gallbladder cancer: A pilot study

Ghada Nouairia, Martin Cornillet, Hannes Jansson, Annika Bergquist, Ernesto Sparrelid

Table of contents

Fig. S1…………………………………………………………………………………...2
Table S1……………………………………………………………………………….....3
Table S2……………………………………………………………………………...21
Table S3……………………………………………………………………………...22
Supplementary references…………………………………………………………... 23

Fig. S1. Complementary data for the methods **(A1).** Singular Value Decomposition Analysis (SVD) showing the correlation of clinical parameters to the proteomic dataset (7 500 proteins). (**A2). Importance of principal components computed in the SVD analysis.** (**B).** Linear regression model iteration with different lambda values and the corresponding Mean-squared error (MSE). The number of proteins selected by each model is indicated at the top.

A1

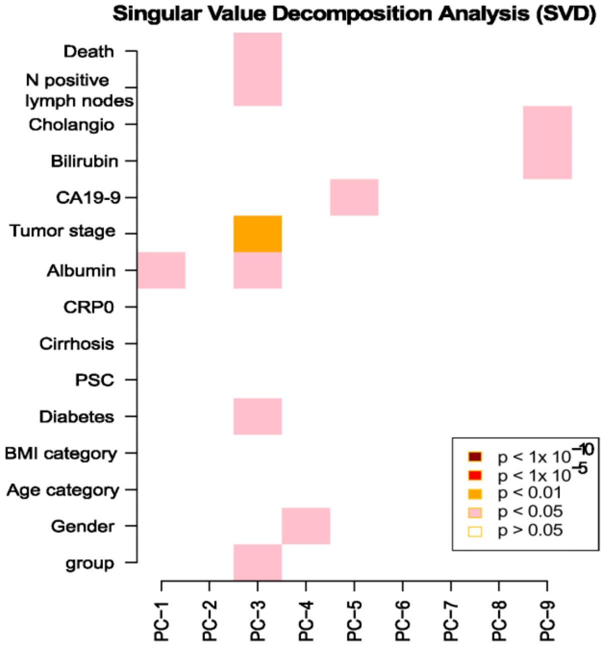

A2

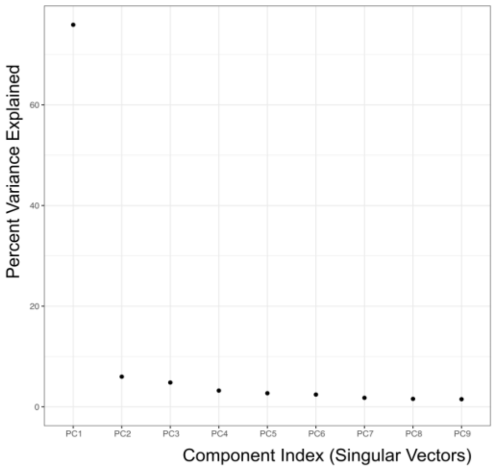

B

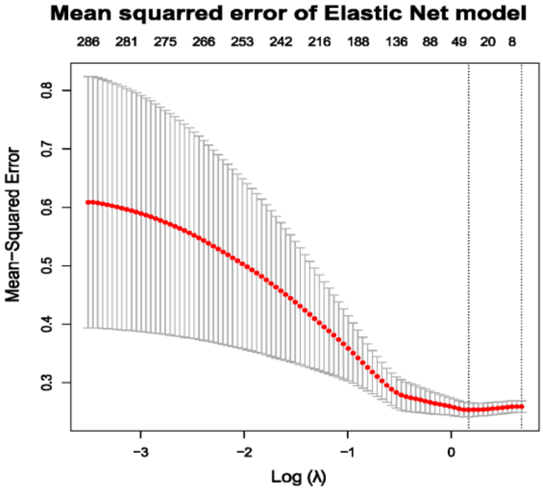

Predicted

Table S1. List of the diagnosis-associated proteins (651) identified by machine learning methods (Elastic Net and LASSO) or significant statistical association based on t-test (Statistical significance cut-off for t-test is p-value < 0.05).
CI: Confidence Interval. EN: Elastic Net. GBC: Gallbladder Cancer. LASSO: Least Absolute Shrinkage and Selection Operator. MD: Mean difference. SMD: Standardized mean difference.

UniProt	Target	SMD	MD	Lower CI	Upper CI	P value	Mean GBC	Mean Cholecystitis	EN	Lasso
**P19419**	ELK1	-0.42	-40.15	-83.42	3.12	0.03	585.32	625.47	TRUE	FALSE
**P09622**	DLDH	0.2	130.02	-165.43	425.47	0.03	1554.17	1424.15	FALSE	FALSE
**P41235**	HNF4A	-0.56	-3391.97	-6009.02	-774.91	0.01	25352.3	28744.26	TRUE	FALSE
**P54274**	TERF1	-0.38	-48.36	-109.26	12.54	0.65	488.35	536.71	TRUE	FALSE
**O75884**	RBBP9	0.07	11.91	-58.74	82.57	0.03	1201.73	1189.82	FALSE	FALSE
**P25440**	BRD2	0.35	98.68	-18.34	215.7	0.14	850.28	751.6	TRUE	FALSE
**P07196**	NFL	0.36	68.81	-13.81	151.43	0.07	618.04	549.23	TRUE	FALSE
**P01106**	c-Myc	-0.28	-613.3	-1664.67	438.07	0.91	320.97	934.27	TRUE	FALSE
**Q9NVQ4**	FAIM1	-0.35	-74.77	-172.37	22.82	0.09	727.23	554.1	TRUE	FALSE
**P09923**	Alkaline phosphatase, intestine	-0.21	-47.13	-148.1	53.84	0.07	680.15	727.28	TRUE	FALSE
**Q92956**	HVEM	-0.06	-16.28	-127.22	94.66	0.02	375.91	416.33	TRUE	FALSE
**P09238**	MMP-10	-0.37	-424.75	-953.15	103.65	0.27	2643.26	3068.01	TRUE	FALSE
**Q96A25**	T106A	-0.34	-58.61	-141.29	24.06	0.07	214.01	272.63	TRUE	FALSE
**Q8WU66**	TSEAR	-0.35	-82.09	-192.3	28.12	0.3	628.6	710.69	TRUE	FALSE
**O00238**	BMP RIB	-0.49	-22.29	-42.96	-1.61	0.02	344.1	366.38	TRUE	FALSE
**Q9H5K3**	SG196	-0.24	-13.75	-37.93	10.44	0.04	313.6	327.34	FALSE	FALSE
**Q9UNP4**	SIAT9	0.07	9.89	-47.46	67.24	0.02	349.6	339.71	FALSE	FALSE
**Q5VY80**	RET1L	-0.22	-67.67	-213.85	78.52	0.41	389.32	376.87	TRUE	FALSE
**Q9NZ53**	PDXL2	-0.11	-53.99	-262.09	154.1	0.05	515.67	569.66	FALSE	FALSE
**Q6UXG2**	K1324:ECD	-0.28	-7.62	-19.57	4.33	0.46	241.83	249.45	TRUE	FALSE
**Q6UXG2**	K1324:CD	-0.28	-7.62	-19.57	4.33	0.46	241.83	249.45	TRUE	FALSE
**Q8N9N2**	ASCC1	-0.2	-27.86	-90.08	34.36	0.59	1076.87	1104.73	TRUE	FALSE
**Q16401**	PSMD5	-0.26	-229.29	-637.95	179.36	0.03	609.58	838.87	FALSE	FALSE
**P0DMV8**	HSP 70	0.38	385.64	-52.92	824.2	0.13	1966.03	1822.48	TRUE	FALSE
**P30740**	Serpin B1	-0.38	-596.31	-1284.88	92.27	0.02	4781.01	5377.32	TRUE	FALSE
**P09603**	CSF-1	-0.11	-1.39	-6.93	4.15	0.96	1566.49	1500.55	TRUE	FALSE
**Q9Y5E7**	PCDB2	-0.36	-282.41	-664.31	99.49	0.01	469.04	751.45	TRUE	FALSE
**Q8IZP9**	GPR64	-0.45	-35.78	-72.39	0.84	0.02	471.68	507.45	TRUE	FALSE
**Q12918**	KLRB1	0.23	20.97	-17.22	59.17	0.03	601.82	748.33	TRUE	FALSE

**Q08554**	Desmocollin-1	-0.32	-30.85	-72.52	10.83	0.03	650.52	681.37	TRUE	FALSE
**Q9UL19**	TIG3	0.1	20.06	-69.93	110.05	0.8	1138.98	1118.92	TRUE	FALSE
**P05113**	IL-5	0.16	63.12	-96.63	222.88	0.2	1859.42	2023.6	TRUE	FALSE
**P04792**	HSP 27	-0.17	-448.48	-1632.15	735.18	0.62	4241.75	4690.23	TRUE	FALSE
**Q8TB22**	SPT20	0.16	702.95	-1112.22	2518.13	0.01	2153.08	1450.13	FALSE	FALSE
**Q9UGC7**	RF1ML	0.45	128.91	6.27	251.55	0.14	967.21	838.29	TRUE	FALSE
**P12109**	Collagen a1(VI)	0.12	79.71	-211.04	370.46	0.12	9964.57	11265.36	TRUE	FALSE
**Q92922**	SMRC1	0.17	943.48	-1328.05	3215.01	0.05	2174.77	1231.29	FALSE	FALSE
**Q9UBS3**	DNJB9	-0.24	-950.38	-2735.62	834.86	0.71	10974	11924.38	TRUE	FALSE
**Q8TAT2**	FGFP3	0.33	77	-26.35	180.34	0.05	1223.5	1146.51	TRUE	FALSE
**P20061**	Holo-TC I	0.11	56.59	-152.76	265.93	0.01	9913.41	11624.43	TRUE	FALSE
**P31415**	CASQ1	-0.01	-14.04	-840.82	812.74	0	7586.03	7600.06	FALSE	FALSE
**P0CG30**	GSTT2	0.43	27.29	0.09	54.5	0.05	427.84	400.55	TRUE	FALSE
**Q9UBS5**	GABR1	0.42	214.9	3.59	426.21	0.42	684	469.1	TRUE	FALSE
**Q8TEY5**	CR3L4	0.35	70	-14.2	154.21	0.43	936.56	866.56	TRUE	FALSE
**Q96EP1**	CHFR	0.09	14.62	-51.99	81.24	0.05	1033.44	1018.81	FALSE	FALSE
**P67870**	CSK2B	-0.29	-105.62	-278.25	67.01	0.74	485.23	590.85	TRUE	FALSE
**O75022**	LIRB3	-0.06	-10.54	-84.82	63.74	0.41	1296.87	2114.11	TRUE	FALSE
**Q96P66**	GP101	0.08	12.97	-52.5	78.44	0.04	480.79	546.44	TRUE	FALSE
**Q9NTX7**	RN146	-0.03	-11.4	-167.57	144.77	0.04	1322.49	1333.89	FALSE	FALSE
**Q8NEZ4**	KMT2C	0.01	1.83	-53.36	57.03	0.69	239.2	237.36	TRUE	FALSE
**P55199**	ELL	0.19	30.65	-39.21	100.52	0.72	1109.63	1078.98	TRUE	FALSE
**Q8TDQ0**	TIMD3	-0.28	-3061.04	-8294.8	2172.72	0.3	2678.87	2770.12	TRUE	FALSE
**O14791**	Apo L1	-0.28	-2440.25	-6188.95	1308.45	0.58	2246.53	2168.7	TRUE	FALSE
**Q3SXY7**	LRIT3	0.39	77.38	-5.1	159.87	0.24	432.63	355.25	TRUE	FALSE
**O95822**	DCMC	0.18	55.09	-89.03	199.21	0.03	760.95	705.86	FALSE	FALSE
**Q8N6T3**	ARFG1	-0.29	-94.79	-240.47	50.9	0.14	1158.99	1253.78	TRUE	FALSE
**P21506**	ZNF10	0.23	26.02	-23.01	75.05	0.19	990.77	964.74	TRUE	FALSE
**O43663**	PRC1	0.07	27.29	-131.68	186.26	0.02	286.76	259.47	FALSE	FALSE
**O43825**	B3GT2	-0.53	-75.17	-140.68	-9.65	0.05	1001.22	1076.39	TRUE	FALSE
**Q9ULW2**	Frizzled-10:CD	-0.11	-38.31	-194.97	118.34	0.01	447.87	557.94	TRUE	FALSE
**Q9ULW2**	Frizzled-10:FZ	-0.11	-38.31	-194.97	118.34	0.01	447.87	557.94	TRUE	FALSE
**Q8IZN3**	ZDH14	0.52	22.57	4.52	40.61	0.06	167.19	144.62	TRUE	FALSE
**Q9NZC2**	TREM2	0.41	422.93	-22.88	868.75	0.12	3373.96	2759.04	TRUE	FALSE
**P07738**	PMGE	-0.51	-142.89	-269.91	-15.86	0.06	539.2	682.09	TRUE	FALSE
**Q9UM13**	APC10	0.22	26.74	-24.18	77.67	0.41	845.71	818.97	TRUE	FALSE
**P06753**	Tropomyosin alpha-3 chain	-0.39	-56.54	-125.73	12.65	0.35	333.73	390.27	TRUE	FALSE
**Q15102**	PA1B3	-0.58	-2428.82	-4364.68	-492.97	0.02	8362.3	10791.13	TRUE	FALSE

**P16152**	CBR1	0.31	370.97	-140.51	882.45	0.03	2900.39	2529.43	FALSE	FALSE
**Q9P2N7**	KLH13	-0.51	-193.18	-372.51	-13.85	0.11	1621.55	1814.72	TRUE	FALSE
**Q9NZA1**	CLIC5	-0.47	-874.91	-1714.99	-34.83	0.14	5648.22	6523.12	TRUE	FALSE
**Q15631**	TSN	-0.47	-134.5	-265.15	-3.85	0.07	1138.8	1273.29	TRUE	FALSE
**Q9ULC4**	MCTS1	-0.64	-264.26	-449.37	-79.14	0	1459.85	1724.11	TRUE	FALSE
**O75347**	TBCA	0.43	322.45	-8.74	653.63	0.03	2797.65	2475.21	TRUE	FALSE
**Q9Y3E7**	CHMP3	0.33	1069.72	-326.45	2465.88	0.2	7637.82	6568.1	TRUE	FALSE
**Q96F10**	SAT2	-0.34	-150.88	-359.03	57.28	0.01	1243.08	1393.96	TRUE	FALSE
**P48643**	TCP-1-epsilon	-0.26	-9299.77	-25052.08	6452.54	0.35	54405.24	63705.01	TRUE	FALSE
**O43281**	EFS	-0.48	-47.98	-93.43	-2.52	0.04	733.99	781.97	TRUE	FALSE
**Q9NQT5**	Exosome component 3	-0.15	-300.77	-1166.32	564.79	0.04	1126.13	1426.9	FALSE	FALSE
**P53365**	ARFP2	-0.26	-949.19	-2740.63	842.24	0.26	1408.27	2357.47	TRUE	FALSE
**P18440**	ARY1	-0.65	-132.7	-222.06	-43.35	0	1212.38	1345.08	TRUE	TRUE
**P43378**	PTN9	-0.23	-10.09	-29.83	9.65	0.82	321.26	331.35	TRUE	FALSE
**O75815**	BCAR3:Ras-GEF	0.47	100.9	9.04	192.76	0.07	963.59	862.69	TRUE	FALSE
**O75815**	BCAR3:SH2	0.47	100.9	9.04	192.76	0.07	963.59	862.69	TRUE	FALSE
**Q96AT9**	RPE	-0.21	-173.81	-562.63	215.01	0.76	1216.17	1389.98	TRUE	FALSE
**Q07866**	KLC1	0.31	1134.44	-432.25	2701.14	0.3	5000.82	3866.37	TRUE	FALSE
**Q13630**	FCL	0.15	167.58	-302.6	637.77	0.02	944.68	777.1	FALSE	FALSE
**Q12923**	PTN13	-0.6	-25.99	-46.45	-5.53	0.21	202.88	228.87	TRUE	FALSE
**Q6YP21**	KAT3	-0.29	-112.02	-290.72	66.69	0.28	696.66	808.68	TRUE	FALSE
**Q9P0V9**	SEP10	-0.23	-1104.66	-3432.14	1222.82	0.55	2596.45	3701.11	TRUE	FALSE
**P50990**	TCP-1-theta	-0.23	-5452.36	-16114.04	5209.32	0.38	37558.86	43011.22	TRUE	FALSE
**Q9HCZ1**	ZN334	-0.43	-67.29	-136.97	2.39	0.13	1399.47	1466.77	TRUE	FALSE
**Q96ER9**	CCD51	0.36	70.67	-10.42	151.75	0.11	587.05	516.39	TRUE	FALSE
**O95989**	NUDT3	-0.18	-35.67	-129.36	58.01	0.8	409.95	445.63	TRUE	FALSE
**P14316**	IRF2	-0.38	-32.2	-70.87	6.47	0.13	291.38	323.58	TRUE	FALSE
**Q15849**	UT2	-0.19	-49.19	-168.7	70.31	0.97	1027.78	1076.98	TRUE	FALSE
**Q86VK4**	ZN410	0.27	11.3	-6.97	29.57	0.11	344.99	333.69	TRUE	FALSE
**O60942**	MCE1	-0.33	-366.97	-912.17	178.22	0.66	4127.81	3969.26	TRUE	FALSE
**P16150**	LEUK	-0.36	-175.59	-411.44	60.26	0.03	632.09	807.68	TRUE	FALSE
**Q8IY33**	MILK2	-0.35	-1202.4	-2841.97	437.17	0.13	1719.53	2921.93	TRUE	FALSE
**Q8IYJ3**	SYTL1	-0.32	-29.99	-73.58	13.61	0.26	458.32	488.31	TRUE	FALSE
**Q9Y6T7**	DGKB	-0.47	-128.43	-250.18	-6.69	0.02	1802.75	1931.19	TRUE	FALSE
**P56278**	MTCP1	0.31	47.26	-17.21	111.73	0.07	863.17	968.92	TRUE	FALSE
**Q8IWT3**	CUL9	-0.32	-8.53	-19.89	2.83	0.04	227.2	235.73	FALSE	FALSE
**O14683**	P5I11	-0.31	-1014.26	-2500.87	472.36	0.25	4100.01	5114.26	TRUE	FALSE
**Q6P2E9**	EDC4	0.31	129.04	-51.15	309.24	0.25	2738.84	2609.79	TRUE	FALSE

**Q13976**	KGP1B	-0.32	-86.03	-203.58	31.52	0.15	1962.95	2048.99	TRUE	FALSE
**P35070**	BTC	0.33	100.8	-24.85	226.44	0.49	473.38	372.58	TRUE	FALSE
**Q9BXY4**	RSPO3	0.33	65.76	-20.44	151.96	0.06	766.93	703.76	TRUE	FALSE
**O43915**	VEGF-D	-0.09	-245.58	-1517.13	1025.97	0.74	1142.72	1300.67	TRUE	FALSE
**Q9BQB4**	SOST	0.18	75.23	-109.61	260.08	0.41	521.18	485.68	TRUE	FALSE
**P10451**	Osteopontin	0.44	71.34	0.36	142.33	0.03	779.89	708.54	TRUE	FALSE
**P41221**	WNT5A	-0.33	-172.85	-400.47	54.77	0.04	1312.78	1485.62	TRUE	FALSE
**Q8N8Q1**	C56D1	-0.55	-259.04	-481.13	-36.95	0.03	1721.86	1980.9	TRUE	FALSE
**O15263**	HBD-2	0.12	958.54	-2375.62	4292.7	0.98	9839.7	8881.16	TRUE	FALSE
**P20155**	2 ISK	0.06	8.23	-50.2	66.67	0.28	383.91	375.67	TRUE	FALSE
**Q8TEU8**	WFKN2	0.18	79.92	-120.48	280.33	0.23	2074.74	1994.81	TRUE	FALSE
**Q15427**	SF3B4	-0.48	-23.11	-45.64	-0.59	0.05	332.88	355.99	TRUE	FALSE
**P0DJ93**	SIM13	0.09	6.63	-26.18	39.44	0.56	593.92	587.29	TRUE	FALSE
**Q9NXH8**	CI167	0.45	29.73	1.31	58.15	0.07	606.68	576.94	TRUE	FALSE
**A8MVW0**	F1712	-0.39	-28.94	-62.21	4.34	0.05	913.81	942.74	TRUE	FALSE
**Q93084**	AT2A3	-0.73	-143.42	-235.08	-51.76	0	1335.81	1479.23	TRUE	TRUE
**Q9UH65**	SWP70	0.5	128.25	18.23	238.28	0.06	1559.91	1431.66	TRUE	FALSE
**Q8IWE4**	DCNL3	0.28	195.46	-92.96	483.88	0.05	740.86	545.4	TRUE	FALSE
**P09211**	Glutathione S-transferase Pi	0.31	132.1	-49	313.2	0.18	3035.13	2903.03	TRUE	FALSE
**O76050**	NEUL1	-0.28	-40.89	-104.82	23.05	0.04	1012.28	1053.17	FALSE	FALSE
**P43363**	MAGE-10	-0.36	-345.71	-766.39	74.96	0.13	5361.17	5706.88	TRUE	FALSE
**P08246**	Elastase	-0.39	-3843.81	-8164.18	476.57	0.02	33753.52	37597.32	TRUE	FALSE
**P19784**	22,00 CSK	-0.3	-13.61	-33.57	6.35	0.99	744.7	1110.41	TRUE	FALSE
**P11926**	Ornithine decarboxylase	-0.39	-70.38	-151.66	10.89	0.16	1320.13	1390.51	TRUE	FALSE
**P07355**	annexin II	0.32	1616.54	-555	3788.08	0.12	964.3	865.05	TRUE	FALSE
**P05155**	C1-Esterase Inhibitor	-0.02	-71.79	-1510.13	1366.56	0.12	5919.91	5544.22	TRUE	FALSE
**Q15485**	FCN2	-0.37	-2647.9	-5732.95	437.16	0.04	57023.67	70010.83	TRUE	FALSE
**P24158**	Proteinase-3	-0.18	-170.52	-569.16	228.11	0	1424.98	1907.94	TRUE	FALSE
**P47736**	RPGP1	-0.39	-81.54	-179.28	16.2	0.02	571.27	652.81	TRUE	FALSE
**P08476**	Inhibin bA chain	0.03	8.15	-107.66	123.95	0.11	691.72	620.27	TRUE	FALSE
**P08476**	Activin A	0.03	8.15	-107.66	123.95	0.11	691.72	620.27	TRUE	FALSE
**P08833**	IGFBP-1	0.51	4259.46	566.5	7952.43	0.01	17101	12841.53	TRUE	FALSE
**Q9GZX6**	IL-22	-0.59	-432.85	-783.13	-82.56	0.02	1701.24	2134.08	TRUE	FALSE
**Q96P47**	AGAP3	0.27	63.02	-35.1	161.14	0.96	669.98	606.97	TRUE	FALSE
**Q08257**	QOR	0.25	1006.24	-833.92	2846.4	0	5054.94	4048.69	FALSE	FALSE
**Q9UHL0**	DDX25	-0.06	-5.58	-45.55	34.39	0.02	422.79	428.37	FALSE	FALSE

**Q92844**	TANK	0.27	413.42	-233.11	1059.96	0.55	2346.75	1933.32	TRUE	FALSE
**Q07011**	4-1BB	-0.36	-25.44	-58	7.11	0.01	440.64	466.09	TRUE	FALSE
**Q96PD4**	IL-17F	0.42	62.42	0.55	124.29	0.02	333.4	453.14	TRUE	FALSE
**P01215**	Glycoprotein hormones a-chain	-0.14	-19.87	-86.64	46.89	0.01	2310.8	1666.12	TRUE	FALSE
**P02747**	C1QC	-0.17	-112.17	-403.92	179.58	0.04	1150.52	1262.69	FALSE	FALSE
**Q16663**	MIP-5	0.51	253.46	41.87	465.06	0.04	1636	1382.54	TRUE	FALSE
**P26447**	S100A4	-0.22	-47.08	-139.88	45.73	0.47	665.52	728.68	TRUE	FALSE
**P27930**	IL-1 sRII	0.03	44.48	-583.94	672.89	1	422.63	520.79	TRUE	FALSE
**Q9NZH7**	IL-1F8	-0.37	-48.52	-107.9	10.86	0.01	623.58	672.1	TRUE	FALSE
**P52797**	Ephrin-A3	-0.21	-42.91	-139.17	53.35	0.05	2232.94	2754.76	FALSE	FALSE
**O75626**	PRDM1	-0.36	-52.99	-118.84	12.86	0.18	867.59	920.58	TRUE	FALSE
**P14649**	MYL6B	-0.19	-33.11	-114.6	48.39	0.65	642.16	675.27	TRUE	FALSE
**P29074**	PTN4	-0.58	-175.68	-312	-39.36	0.02	2130.5	2306.18	TRUE	FALSE
**Q9BYN0**	SRXN1	0.39	57.67	-5.24	120.59	0.39	478.13	420.45	TRUE	FALSE
**P31943**	HNRH1	-0.01	-10.66	-409.36	388.04	0	2549.44	2560.1	FALSE	FALSE
**O43617**	TPPC3	-0.48	-145.96	-284.24	-7.68	0.07	1200.46	1346.42	TRUE	FALSE
**P17655**	CAN2	0.41	496.64	-21.02	1014.31	0.07	4119.6	3622.96	TRUE	FALSE
**P35236**	PTN7	0.45	39.4	2.42	76.37	0.09	303.66	264.27	TRUE	FALSE
**Q15828**	Cystatin M	-0.58	-4290.66	-7581.86	-999.46	0.01	4452.36	5491.6	TRUE	FALSE
**O75462**	CRLF1	-0.04	-13.81	-163.96	136.34	0.01	423.93	473.2	TRUE	FALSE
**Q9H6E4**	CC134	0.05	72.42	-580.66	725.51	0.18	273.39	301.54	TRUE	FALSE
**Q9Y281**	COF2	-0.32	-110.47	-264.98	44.04	0.12	511.3	621.76	TRUE	FALSE
**P01042**	Kininogen, HMW, Two Chain	0.13	491.66	-1084.86	2068.18	0.33	3328.97	3512.56	TRUE	FALSE
**P01042**	Kininostatin	0.13	491.66	-1084.86	2068.18	0.33	3328.97	3512.56	TRUE	FALSE
**P01042**	Kininogen, HMW	0.13	491.66	-1084.86	2068.18	0.33	3328.97	3512.56	TRUE	FALSE
**Q8N8U9**	BMPER	0.33	131.27	-38.11	300.64	0.32	1686.05	1554.79	TRUE	FALSE
**P15086**	Carboxypeptidase B1	0.16	484.39	-832.24	1801.03	0.26	3067.36	2582.97	TRUE	FALSE
**P12104**	FABP2	0.41	286.61	-7.32	580.55	0.13	1731.22	1444.6	TRUE	FALSE
**P55789**	HERV1	0.25	105.09	-79.37	289.55	0.3	1323.79	1218.7	TRUE	FALSE
**P05014**	IFNA4	0.26	14.29	-9.06	37.63	0.96	308.56	294.28	TRUE	FALSE
**P00338**	LDHA	0.14	1592.31	-3225.93	6410.55	0.23	728.32	839.55	TRUE	FALSE
**P16519**	NEC2	-0.24	-867.14	-2629.36	895.08	0.05	3298.9	3103.55	TRUE	FALSE
**Q02297**	SMDF	0.07	63.73	-338.45	465.91	0.54	190.9	161.32	TRUE	FALSE
**Q02297**	NEUREGULIN-1	0.07	63.73	-338.45	465.91	0.54	190.9	161.32	TRUE	FALSE
**P01732**	CD8A	-0.66	-2730.88	-4570.98	-890.78	0.01	1371.73	1631.3	TRUE	TRUE
**P01732**	CD8A	-0.66	-2730.88	-4570.98	-890.78	0.01	12890.82	15621.7	TRUE	FALSE

**Q6UW15**	REG3G	0.18	1478.58	-1914.26	4871.42	0.41	506.92	568.95	TRUE	FALSE
**P19801**	ABP1	-0.37	-705.89	-1554.02	142.23	0.07	3068.27	3774.16	TRUE	FALSE
**P54802**	NAG	-0.37	-679.49	-1483.62	124.64	0.05	5396.89	6076.38	TRUE	FALSE
**P06276**	Pseudocholinesterase	-0.62	-1165.52	-1986.26	-344.78	0	55717.04	64338.51	TRUE	FALSE
**P63098**	Calcineurin B a	0.38	378.68	-55.01	812.37	0.17	5112.08	4733.39	TRUE	FALSE
**Q07075**	AMPE	-0.64	-357.55	-603.93	-111.18	0	2260.21	2617.76	TRUE	FALSE
**Q8WXI7**	CA125	-0.09	-16.6	-105.36	72.15	0	102.98	119.58	FALSE	FALSE
**P20023**	Complement receptor type 2	-0.46	-1056.65	-2099.39	-13.91	0.06	4814.11	5870.76	TRUE	FALSE
**P08887**	IL-6 sRa	-0.61	-372.05	-647.64	-96.46	0.01	10069.52	11292.57	TRUE	FALSE
**P17301**	Integrin alpha-2	0.39	55.13	-3.29	113.54	0.14	326.21	271.09	TRUE	FALSE
**Q9HAP6**	LIN7B	0.02	2.92	-66.21	72.05	0.01	870.03	395.61	FALSE	FALSE
**P02753**	RBP	-0.49	-414.5	-784.68	-44.33	0.02	25739.17	28642.1	TRUE	FALSE
**P43251**	Biotinidase	-0.01	-5.17	-183.73	173.39	0.35	13135.02	13674.02	TRUE	FALSE
**Q03692**	COAA1	0.37	212.56	-27.53	452.66	0.01	747.24	534.67	TRUE	FALSE
**P15056**	BRAF1	-0.27	-94.89	-258.15	68.38	0.39	1346.32	1441.21	TRUE	FALSE
**Q9UBU2**	2,00 DKK	0.3	1917.66	-794.78	4630.11	0	682.2	574.24	TRUE	TRUE
**Q9UBU2**	2,00 DKK	0.3	1917.66	-794.78	4630.11	0.59	17335.92	15418.26	TRUE	FALSE
**P35916**	VEGF sR3	-0.37	-527.06	-1154.32	100.2	0.08	6392.57	6919.63	TRUE	FALSE
**O75473**	LGR5	-0.36	-55.95	-125.71	13.81	0.19	812.34	868.29	TRUE	FALSE
**Q9BXB1**	LGR4	0.28	915.73	-454.94	2286.39	0.49	2412.87	1497.14	TRUE	FALSE
**P55285**	Cadherin-6	0.02	2.82	-54.9	60.53	0.1	1628.13	1707.75	TRUE	FALSE
**Q13158**	FADD	-0.48	-182.31	-348.57	-16.06	0.02	2976.25	3158.57	TRUE	FALSE
**Q86SX6**	GLRX5	-0.44	-60.54	-123.24	2.16	0.02	268.53	329.07	TRUE	FALSE
**O75056**	SDC3	-0.36	-166.13	-370.5	38.24	0.22	1689.03	1855.16	TRUE	FALSE
**Q2MKA7**	RSPO1	0.49	341.43	37.57	645.28	0.01	2344.11	2002.68	TRUE	FALSE
**P55107**	BMP-3b	0.25	48.58	-35.57	132.73	0.28	1398.35	1349.76	TRUE	FALSE
**P16860**	BNP	0.04	4.03	-35.94	43.99	0.03	481.26	460.29	TRUE	FALSE
**P16860**	BNP-32	0.04	4.03	-35.94	43.99	0.03	481.26	460.29	TRUE	FALSE
**P16860**	N-terminal pro-BNP	0.04	4.03	-35.94	43.99	0.03	481.26	460.29	TRUE	FALSE
**Q7Z4P5**	GDF7	0.34	45.57	-10.98	102.12	0.59	417.54	382.46	TRUE	FALSE
**Q01523**	HD-5	0.5	592.9	78.53	1107.26	0.03	3500.52	2907.63	TRUE	FALSE
**P30043**	BLVRB	-0.62	-11219.94	-19470.07	-2969.81	0.02	44542.11	55762.05	TRUE	FALSE
**O15075**	DCAK1	-0.27	-294.92	-801.02	211.18	0.68	2890.78	3185.7	TRUE	FALSE
**P12429**	Annexin III	-0.44	-168.95	-354.32	16.42	0.04	235.36	404.3	TRUE	FALSE
**P52564**	MP2K6	-0.21	-22.11	-70.74	26.52	0.76	621.35	643.46	TRUE	FALSE
**P13796**	L-plastin	-0.28	-888.71	-2274.74	497.32	0.04	19879.48	20768.19	FALSE	FALSE

**Q96MU8**	KREM1	0.12	23.84	-62.5	110.18	0.53	3213.13	3079.79	TRUE	FALSE
**Q70SY1**	CR3L2	0.37	24.17	-3.63	51.96	0.46	223.58	199.41	TRUE	FALSE
**O15079**	SNPH	0.28	47.51	-24.6	119.62	0.04	520.12	472.61	FALSE	FALSE
**P13804**	ETFA	-0.46	-117.44	-230.44	-4.44	0.03	1969.41	2086.85	TRUE	FALSE
**P21926**	CD9	-0.32	-115.8	-292.45	60.85	0.13	576.65	692.45	TRUE	FALSE
**P15328**	FOLR1	-0.47	-101.87	-203.48	-0.26	0.07	696.54	798.41	TRUE	FALSE
**Q9UNL4**	ING4	-0.21	-28.44	-88.15	31.27	0.01	533.15	561.59	FALSE	FALSE
**Q92598**	HS105	0.54	65.51	13.37	117.65	0.02	635.87	570.36	TRUE	FALSE
**Q9Y6N9**	USH1C	-0.26	-22.69	-62.52	17.14	0.21	386.9	409.59	TRUE	FALSE
**Q96EY8**	MMAB	0.27	675.51	-419.87	1770.88	0.36	5058.86	4383.36	TRUE	FALSE
**P37235**	HPCL1	-0.28	-95.78	-256.16	64.61	0.51	951.36	1042.93	TRUE	FALSE
**Q9NVS9**	PNPO	0.41	64.21	-2.55	130.97	0.12	1072.05	1007.84	TRUE	FALSE
**Q9BXS1**	IDI2	0.13	16.3	-41.23	73.83	0.4	307.29	290.99	TRUE	FALSE
**Q96FQ6**	S100A16	0.19	100.73	-129.61	331.06	0.47	2345.87	2245.15	TRUE	FALSE
**P49798**	RGS4	-0.25	-80.1	-235.33	75.12	0.03	781.67	834.71	TRUE	FALSE
**Q9Y294**	ASF1A	-0.3	-226.67	-588.08	134.73	0.23	870.12	1096.8	TRUE	FALSE
**Q96F85**	CB032	-0.59	-1212	-2149.7	-274.29	0.03	5767.72	6979.71	TRUE	FALSE
**Q9UKY0**	PRND	-0.56	-214.4	-382.38	-46.42	0.01	2156.87	2371.27	TRUE	FALSE
**A6NDG6**	PGP	-0.21	-159.65	-507.88	188.59	0.64	2576.86	2736.51	TRUE	FALSE
**Q04837**	SSB	0.49	518.81	75.89	961.74	0.01	1459.98	941.17	TRUE	FALSE
**Q9P287**	BCCIP	0.25	21.01	-15.32	57.34	0.5	991.41	970.4	TRUE	FALSE
**Q9H2U2**	PPase 2	0.29	73.24	-35.58	182.06	0.29	1071.55	998.31	TRUE	FALSE
**Q14194**	DRP-1	-0.3	-39.44	-101.91	23.02	0.59	344.29	383.73	TRUE	FALSE
**Q96G03**	PGM2	-0.27	-65.72	-178.2	46.75	0.64	995.21	1060.93	TRUE	FALSE
**P04062**	GLCM	0.21	309.07	-330.24	948.38	0.31	5430.15	5121.08	TRUE	FALSE
**P21964**	Catechol O-methyltransferase	-0.34	-77.69	-182.29	26.92	0.22	1312.46	1390.14	TRUE	FALSE
**P84085**	ARF5	-0.02	-4.41	-88.46	79.64	0.82	820.24	824.65	TRUE	FALSE
**Q9H0F7**	6,00 ARL	0.35	406.34	-84.31	896.99	0.39	2782.04	2375.71	TRUE	FALSE
**P62380**	TBPL1	0.12	20.14	-56.1	96.37	0.66	560.76	540.62	TRUE	FALSE
**Q96EQ0**	SGTB	-0.32	-434.04	-1089.9	221.82	0.01	455.94	889.98	TRUE	FALSE
**P62837**	UB2D2	0.22	17.91	-17.64	53.46	0.12	631.88	613.97	TRUE	FALSE
**P35030**	3,00 TRY	0.23	582.79	-493.49	1659.07	0.1	360.97	396.96	TRUE	FALSE
**Q9BQI0**	AIF1L	0.35	125.51	-31.69	282.7	0.09	1851.54	1726.03	TRUE	FALSE
**P49715**	CEBPA	0.18	220.28	-283.52	724.08	0.05	517.15	296.87	FALSE	FALSE
**Q9H4D0**	CSTN2	0.65	90.29	31.28	149.3	0	564.27	473.98	TRUE	TRUE
**A6NFH5**	FBP12	-0.3	-100.7	-264.56	63.17	0.26	828.54	929.24	TRUE	FALSE
**P32456**	GBP2	0.42	155.52	-7.7	318.75	0.04	1870.86	1715.34	TRUE	FALSE
**Q9Y3E1**	HDGR3	0.3	28.76	-12.45	69.97	0.19	328.48	299.72	TRUE	FALSE

**O94813**	SLIT2	0.21	209.7	-216.38	635.79	0.4	4038.96	3829.26	TRUE	FALSE
**O75094**	SLIT3	0.37	159.33	-29.62	348.27	0.04	2243.14	2083.82	TRUE	FALSE
**Q9HAN9**	NMNA1	-0.49	-109.38	-214.52	-4.25	0.46	543.34	652.73	TRUE	FALSE
**P49221**	TGM4	-0.29	-21.07	-53.44	11.29	0.03	370.72	391.8	TRUE	FALSE
**Q8NI38**	IKBD	-0.31	-512.72	-1303.66	278.22	0.03	1715.83	2228.55	TRUE	FALSE
**Q02252**	MMSA	0.2	1477.7	-1721.23	4676.62	0.5	9223.7	7746	TRUE	FALSE
**Q8N565**	MREG	-0.63	-74.96	-128.39	-21.52	0.01	854.42	929.37	TRUE	FALSE
**Q9HCU8**	DNA polymerase subunit delta 4	-0.28	-63.59	-174.1	46.92	0.19	409.97	473.55	TRUE	FALSE
**O14933**	UB2L6	0.22	23.68	-25.28	72.65	0.02	802.36	778.68	FALSE	FALSE
**P36639**	8ODP	-0.41	-69.55	-150.72	11.62	0.04	644.17	713.71	TRUE	FALSE
**Q92597**	NDRG1	0.3	372.16	-148.1	892.41	0.4	1427.07	1054.92	TRUE	FALSE
**Q16775**	GLO2	-0.43	-5088.64	-10407.73	230.45	0.08	41887.21	46975.85	TRUE	FALSE
**O14958**	CASQ2	0.11	17.99	-51.8	87.78	0.12	418.81	400.82	TRUE	FALSE
**Q01449**	MLRA	-0.56	-568.22	-1024.93	-111.51	0.02	4255.16	4823.37	TRUE	FALSE
**Q6UWS5**	PT117	-0.41	-30.24	-63.06	2.59	0.07	533.88	564.12	TRUE	FALSE
**Q86WC4**	OSTM1	-0.34	-7.1	-16.16	1.96	0.03	293.85	300.96	TRUE	FALSE
**O95897**	NOE2	-0.39	-267.15	-578.33	44.03	0.03	24573.57	27861.95	TRUE	FALSE
**Q9BW66**	CINP	-0.37	-371.24	-861.15	118.67	0.52	682.02	1053.26	TRUE	FALSE
**P55289**	Cadherin-12:ECD	0.21	56.52	-59.02	172.06	0.02	2071.17	791.17	TRUE	FALSE
**P55289**	Cadherin-12:CD	0.21	56.52	-59.02	172.06	0.02	2071.17	791.17	TRUE	FALSE
**Q16623**	STX1a	-0.23	-259.2	-809.49	291.09	0.56	430.97	418.94	TRUE	FALSE
**Q86Z14**	KLOTB	-0.44	-128.73	-259.82	2.37	0.05	717.89	846.61	TRUE	FALSE
**P00533**	EGFRvIII	0.17	869.47	-1268.07	3007.02	0.12	11585.47	12290.21	TRUE	FALSE
**P00533**	ERBB1	0.17	869.47	-1268.07	3007.02	0.12	11585.47	12290.21	TRUE	FALSE
**P24593**	IGFBP-5	-0.07	-131.24	-967.55	705.07	0	53106.23	57784.16	TRUE	FALSE
**Q96DX5**	ASB9	0.32	321.09	-117.59	759.76	0.13	2965.96	2644.88	TRUE	FALSE
**P08865**	40S ribosomal protein SA	-0.43	-70.98	-148.56	6.61	0.25	815.33	886.31	TRUE	FALSE
**P10997**	IAPP	0.26	188.82	-127.04	504.67	0.31	2616.4	2427.59	TRUE	FALSE
**Q6UX27**	VSTM1	-0.41	-92.95	-193.58	7.68	0.04	1472.06	1565.01	FALSE	FALSE
**P15085**	CBPA1	0.22	310.83	-286.19	907.85	0.94	3035.87	2725.04	TRUE	FALSE
**Q712K3**	UB2R2	-0.5	-141.05	-266.49	-15.6	0.05	2265.72	2406.77	TRUE	FALSE
**O75897**	ST1C4	0.31	30.53	-10.43	71.49	0.58	274.89	244.36	TRUE	FALSE
**Q86WK6**	AMGO1:ECD	-0.04	-3.03	-37.97	31.9	0.04	306.01	309.05	FALSE	FALSE
**Q86WK6**	AMGO1:CD	-0.04	-3.03	-37.97	31.9	0.04	306.01	309.05	FALSE	FALSE
**Q92630**	DYRK2	-0.23	-5.67	-16.38	5.03	0.02	211.25	216.92	FALSE	FALSE
**Q96PP8**	GBP5	-0.36	-142.79	-320.31	34.73	0.15	1721.58	1864.37	TRUE	FALSE

**O95045**	UPP2	0.36	31.47	-5.3	68.25	0.19	490.92	459.44	TRUE	FALSE
**Q969E8**	TSR2	-0.31	-123.27	-303.94	57.39	0.38	1350.25	1473.52	TRUE	FALSE
**Q6PID6**	TTC33	-0.33	-1155.81	-2867.09	555.47	0.04	3067.85	4223.66	TRUE	FALSE
**Q03014**	HHEX	-0.42	-59.36	-125.92	7.2	0.17	683.81	743.18	TRUE	FALSE
**P23582**	Natriuretic Peptide C-Type	0.25	13.34	-9.58	36.27	0.21	479.17	465.82	TRUE	FALSE
**A6NKN8**	PC4L1	-0.15	-26.25	-103.25	50.75	0.04	332.36	358.61	FALSE	FALSE
**Q68CL5**	TPGS2	-0.53	-138.37	-253.4	-23.35	0.02	2812.98	2951.35	TRUE	FALSE
**Q9UKA8**	RCAN3	-0.47	-675.01	-1309.87	-40.15	0.05	5823.76	6498.77	TRUE	FALSE
**Q9BW30**	TPPP3	0.24	219.1	-176.49	614.69	0.36	2943.44	2724.34	TRUE	FALSE
**Q9NY72**	SCN3B	-0.49	-48.01	-93.64	-2.37	0.07	337.04	385.05	TRUE	FALSE
**Q9Y6N8**	CAD10	-0.46	-16.97	-33.39	-0.56	0.07	252.84	269.82	TRUE	FALSE
**Q8NFR9**	I17RE	-0.29	-114.34	-294.06	65.38	0.46	1310.33	1424.67	TRUE	FALSE
**P59666**	HNP-3	-0.43	-1481.13	-3015.48	53.21	0.03	5485.4	6966.54	TRUE	FALSE
**Q9NTN9**	SEM4G	-0.39	-182.95	-389.14	23.24	0.12	2042.16	2225.11	TRUE	FALSE
**P29016**	CD1B	-0.24	-895.15	-2692.79	902.5	0.03	144.85	1039.99	TRUE	FALSE
**P01303**	NPY	0.43	132.26	0.43	264.1	0.09	1643.04	1510.77	TRUE	FALSE
**Q9Y2C3**	B3GT5	-0.11	-56.07	-296.91	184.77	0.04	1368.34	1424.41	FALSE	FALSE
**Q9Y4U1**	MMAC	-0.24	-197.23	-553.78	159.32	0.01	2381.03	2578.26	FALSE	FALSE
**Q9NZV6**	MSRB1	-0.45	-68.29	-138.57	1.98	0.1	809.35	877.65	TRUE	FALSE
**Q8TA86**	RP9	0.27	86.97	-55.85	229.79	0.27	1635.06	1548.09	TRUE	FALSE
**Q5SZJ8**	BEND6	-0.33	-20.88	-50.15	8.38	0.02	299.47	320.35	FALSE	FALSE
**Q8WUW1**	BRK1	-0.35	-82.81	-189.11	23.5	0.14	1397.22	1480.03	TRUE	FALSE
**A6NIH7**	U119B	-0.21	-657.54	-2166.22	851.15	0.94	1816.62	2474.16	TRUE	FALSE
**Q96A32**	MLRS	-0.31	-406.03	-1003.31	191.25	0.3	3075.34	3481.37	TRUE	FALSE
**P24941**	CDK2	-0.13	-185.94	-827.69	455.81	0.55	367.43	358.22	TRUE	FALSE
**O00141**	SGK1	-0.4	-33.01	-68.24	2.22	0.01	782.35	815.37	TRUE	FALSE
**O00294**	TULP1	-0.37	-174.32	-405.1	56.45	0.01	184.84	359.16	TRUE	FALSE
**A2RU49**	HYKK	0.33	299.48	-74.73	673.68	0.26	951.48	652.01	TRUE	FALSE
**Q8IUE1**	TF2LX	0.34	55.33	-12.58	123.23	0.72	226.16	170.84	TRUE	FALSE
**Q9H1K6**	MESD1	-0.64	-481.85	-828.27	-135.43	0.02	2389.72	2871.57	TRUE	FALSE
**Q9P2M1**	LR2BP	-0.01	-4.53	-162.01	152.95	0.01	1197.45	1201.98	FALSE	FALSE
**Q6QNY1**	BL1S2	-0.4	-80.55	-178.77	17.67	0.23	168.41	248.96	TRUE	FALSE
**Q9H596**	DUS21	0.43	29.63	-0.16	59.41	0.07	377.21	347.58	TRUE	FALSE
**Q14863**	PO6F1	-0.12	-522.58	-2349.51	1304.35	0.03	6383.34	6905.93	FALSE	FALSE
**Q9GZZ9**	UBA5	-0.36	-29.39	-65.99	7.22	0.11	487.93	517.32	TRUE	FALSE
**P21579**	SYT1	-0.53	-60.46	-114.11	-6.82	0.02	406.17	466.63	TRUE	FALSE
**Q6ZN17**	LN28B	-0.01	-1.07	-38.88	36.75	0.03	338.88	339.95	FALSE	FALSE
**Q8WUP2**	FBLI1	0.49	669.05	99.61	1238.48	0.29	1601.19	932.15	TRUE	FALSE

**Q96BD8**	SKA1	-0.18	-24.68	-82.32	32.95	0.04	776.68	801.36	FALSE	FALSE
**Q8IUF8**	MINA	0.45	43.48	2.69	84.27	0.04	348.75	305.27	TRUE	FALSE
**Q8TBN0**	R3GEF	-0.19	-517.47	-1774.26	739.31	0.04	1120.42	1637.89	FALSE	FALSE
**P0DN86**	b-CF	0.43	376.72	9.85	743.59	0.07	1770.11	1393.39	TRUE	FALSE
**Q16584**	M3K11	-0.29	-1320.36	-3443.76	803.05	0.04	7889.32	9209.68	TRUE	FALSE
**Q8WYN0**	ATG4A	-0.37	-38.39	-84.13	7.34	0.11	416.08	454.47	TRUE	FALSE
**Q969S2**	NEIL2	0.4	11.22	-0.54	22.99	0.08	152.28	141.06	TRUE	FALSE
**Q15846**	CLUL1	0.35	102.82	-21.39	227.03	0.38	1342.79	1239.98	TRUE	FALSE
**Q99807**	COQ7	0.19	115.6	-144.83	376.03	0.05	1289.52	1173.92	FALSE	FALSE
**Q96Q40**	CDK15	-0.36	-341.83	-763.19	79.52	0.08	5521.67	5863.51	TRUE	FALSE
**O60888**	CUTA	-0.28	-39.06	-100.37	22.25	0.03	795.39	834.45	FALSE	FALSE
**Q8NFZ3**	NLGNY	0.3	14.72	-5.48	34.91	0.45	223.14	208.42	TRUE	FALSE
**O14514**	BAI1	-0.28	-16.55	-42.26	9.16	0.04	286.85	303.4	TRUE	FALSE
**O75191**	XYLB	-0.33	-138.49	-336.38	59.39	0.83	766.63	905.13	TRUE	FALSE
**P10321**	HLA-C	0.23	3171.07	-2756.35	9098.5	0.53	20077.65	16906.58	TRUE	FALSE
**P18615**	NELFE	0.18	275.73	-343.44	894.89	0.02	1060.85	785.13	FALSE	FALSE
**P29508**	SCCA1	0.43	168.53	2.06	335	0.03	1400.45	1231.92	TRUE	FALSE
**P24539**	AT5F1	-0.38	-72.72	-162.92	17.48	0.62	2113.04	2248.94	TRUE	FALSE
**P35226**	BMI-1	-0.27	-146.27	-410.98	118.44	0.07	422.88	569.15	TRUE	FALSE
**Q9UBR1**	BUP1	0.16	51.32	-89.91	192.55	0.01	733.89	682.57	FALSE	FALSE
**Q8IZL9**	CDK20	0.15	32.01	-65.51	129.53	0.02	705.42	673.41	FALSE	FALSE
**P01033**	TIMP-1	0.31	451.19	-191.6	1093.97	0.14	7406.27	6955.08	TRUE	FALSE
**O60519**	CRBL2	-0.45	-23.99	-48.56	0.58	0.03	396.83	420.82	TRUE	FALSE
**P32242**	OTX1	0.11	42.37	-111.18	195.92	0.03	371.62	329.26	FALSE	FALSE
**Q96QV6**	H2A1A	0.46	1335.14	103.54	2566.74	0.1	4890.14	3555.01	TRUE	FALSE
**O60814**	H2B1K	0.44	557.63	23.12	1092.14	0.07	1972.76	1415.13	TRUE	FALSE
**Q96FA3**	PELI1	-0.35	-61.73	-145.94	22.47	0.02	221.3	283.03	TRUE	FALSE
**A2RU54**	HMX2	-0.74	-70.74	-115.14	-26.35	0.01	581.22	651.96	TRUE	TRUE
**Q99081**	HTF4	-0.27	-735.1	-1964.19	493.98	0.18	4835.99	5571.09	TRUE	FALSE
**Q6ZST4**	LCNL1	0.42	44.86	0.34	89.38	0.04	283.1	238.24	TRUE	FALSE
**Q9P086**	MED11	-0.28	-169.37	-452.76	114.01	0.05	1094.11	1263.48	TRUE	FALSE
**P29558**	RBMS1	0.42	254.9	-1.63	511.43	0.19	1617.93	1363.02	TRUE	FALSE
**Q9NNX6**	DC-SIGN	0.11	64.28	-185.13	313.69	0.15	2522.15	2200.3	TRUE	FALSE
**P58400**	NRX1B	0.31	13.78	-5.33	32.88	0.27	296.89	283.11	TRUE	FALSE
**Q9UF33**	EPHA6	0.16	326.78	-570.9	1224.47	0.45	7942.85	7616.07	TRUE	FALSE
**Q9HBM1**	SPC25	-0.44	-259.21	-525.76	7.35	0.03	1686.07	1945.28	TRUE	FALSE
**Q5MJ09**	SPXN3	-0.23	-15.58	-45.68	14.51	0.2	281.46	297.05	TRUE	FALSE
**Q9H0W7**	THAP2	0.26	296.7	-181.33	774.74	0.05	1289.24	992.53	FALSE	FALSE
**P10828**	THB	0.59	389	110.5	667.49	0.02	3032.2	2643.2	TRUE	FALSE

**Q9UBT3**	Dkk-4	0.13	15.86	-39.03	70.76	0.99	7988.56	7173.85	TRUE	FALSE
**Q13323**	BIK	-0.47	-882.15	-1780.64	16.33	0.02	1118.12	2000.28	TRUE	FALSE
**P29376**	LTK	-0.53	-38.41	-72.13	-4.69	0.03	409.67	448.08	TRUE	FALSE
**Q9HAE3**	EFCB1	0.29	100.12	-44.5	244.74	0.82	901.18	801.06	TRUE	FALSE
**Q8WVD5**	RN141	0.44	146.41	7.81	285.02	0.08	927.91	781.5	TRUE	FALSE
**Q5T4F7**	SARP-3	0.35	555.92	-112.08	1223.93	0.31	2769.37	2213.44	TRUE	FALSE
**Q6UWP2**	DHR11	-0.35	-519.5	-1189.73	150.73	0.14	4366.96	4886.46	TRUE	FALSE
**Q96MC5**	CP045	0.31	612.42	-202.5	1427.34	0.58	2445.2	1832.77	TRUE	FALSE
**Q14565**	DMC1	-0.32	-38.62	-91.39	14.16	0.05	907.33	945.95	TRUE	FALSE
**Q8TC99**	FNDC8	-0.41	-21.18	-44.41	2.05	0.07	420.41	441.59	TRUE	FALSE
**O14832**	PAHX	-0.7	-315.3	-516.52	-114.07	0	2300.2	2615.5	TRUE	TRUE
**Q9UFW8**	CGBP1	-0.1	-3.07	-16.39	10.25	0.01	99.31	102.38	FALSE	FALSE
**Q96GK7**	FAH2A	-0.29	-205.02	-530.45	120.4	0.3	1354.9	1559.92	TRUE	FALSE
**Q8N7B1**	HORM2	-0.33	-21.76	-52.14	8.62	0.03	222.15	243.91	FALSE	FALSE
**P57768**	Sorting nexin 16	-0.21	-31	-95.96	33.97	0.58	560.39	591.38	TRUE	FALSE
**Q9UL42**	PNMA2	-0.33	-81.86	-193.27	29.54	0.05	1399.64	1481.5	TRUE	FALSE
**Q8TAM6**	ERMIN	-0.1	-6.97	-36.35	22.42	0.03	359.61	366.58	FALSE	FALSE
**P55042**	RAD	-0.08	-19.81	-141.99	102.37	0.07	368.65	388.46	TRUE	FALSE
**Q9BZL3**	SMIM3	0.38	105.18	-12.12	222.48	0.11	947.87	842.68	TRUE	FALSE
**Q9UBU3**	Obestatin	0.54	126.27	25.79	226.75	0.04	1114.36	988.09	TRUE	FALSE
**Q9UBU3**	ghrelin	0.54	126.27	25.79	226.75	0.04	1114.36	988.09	TRUE	FALSE
**Q96AT1**	K1143	-0.27	-44.42	-121.03	32.19	0.43	1066.4	1110.82	TRUE	FALSE
**Q5VUM1**	CF057	-0.27	-26.21	-69.92	17.5	0.02	344.58	370.78	FALSE	FALSE
**Q8NDH3**	PEPL1	-0.33	-78.25	-192.18	35.68	0.09	308.27	386.52	TRUE	FALSE
**Q9NZN4**	EHD2	0.4	1038.02	-102.29	2178.33	0.05	11215.13	10177.11	TRUE	FALSE
**Q9UJ83**	HACL1	-0.28	-84.03	-228.58	60.52	0.27	657.75	741.78	TRUE	FALSE
**Q9BST9**	RTKN	-0.66	-67.93	-115.38	-20.48	0.01	545.36	613.29	TRUE	FALSE
**Q14833**	GRM4	0.29	1427.5	-604.45	3459.44	0.2	2057.4	629.9	TRUE	FALSE
**P35914**	HMGCL	0.26	557.45	-371.64	1486.53	0.26	3592.25	3034.81	TRUE	FALSE
**Q8N5L8**	RP25L	0.33	163.16	-43.44	369.77	0.06	935.22	772.06	TRUE	FALSE
**Q6NUJ5**	PWP2B	0.35	28.39	-7.54	64.33	0.04	736.53	708.14	TRUE	FALSE
**Q5VW32**	BROX	0.12	31.57	-79.41	142.56	0.29	1176.91	1145.34	TRUE	FALSE
**Q8N4X5**	AF1L2	0.46	155.46	12.38	298.53	0.19	758.18	602.72	TRUE	FALSE
**P53701**	CCHL	0.21	38.36	-39.69	116.4	0.03	486.46	448.1	FALSE	FALSE
**Q9Y6X0**	SETBP	0.34	39.04	-9.26	87.35	0.52	543.13	504.09	TRUE	FALSE
**Q6QNY0**	BL1S3	0.44	78.49	3.42	153.57	0.32	941.73	863.24	TRUE	FALSE
**P57055**	DSCR6	0.43	537.23	11.29	1063.16	0.07	1513.17	975.95	TRUE	FALSE
**P21549**	SPYA	0.11	72.35	-238.57	383.28	0.04	623.06	550.7	FALSE	FALSE

**Q5UCC4**	INM02	-0.45	-31.22	-61.3	-1.14	0.11	443.61	474.83	TRUE	FALSE
**Q9H3N1**	TXND1	0.06	49.77	-313.04	412.59	0.03	1401.17	1351.4	TRUE	FALSE
**O15266**	SHOX	-0.4	-48.3	-106.4	9.8	0.05	229.85	278.15	TRUE	FALSE
**Q4G0F5**	VP26B	-0.24	-58.04	-173.27	57.19	0.05	366.91	424.95	FALSE	FALSE
**Q86WN1**	FCSD1	-0.01	-4.15	-242.34	234.04	0.03	1548.08	1552.23	FALSE	FALSE
**P51178**	Phospholipase C-delta-1	0.04	24.15	-277.7	326	0.23	2337.75	2313.6	TRUE	FALSE
**P35712**	SOX6	-0.48	-52.22	-103.01	-1.44	0	368.4	420.62	TRUE	FALSE
**Q7Z5H3**	RHG22	-0.57	-12.53	-22.52	-2.55	0.01	199.97	212.5	TRUE	FALSE
**P48382**	RFX5	0.24	48.45	-36.07	132.97	0.45	860.25	811.81	TRUE	FALSE
**Q9ULA0**	DNPEP	-0.37	-51.93	-113.74	9.89	0.05	796.84	848.77	TRUE	FALSE
**O60609**	GFRa-3	0.24	47.92	-38.58	134.43	0.4	999.58	951.65	TRUE	FALSE
**P29144**	TPP2	-0.29	-356.18	-906.68	194.32	0.48	1958.11	2314.29	TRUE	FALSE
**Q8IUI8**	CRLF3	-0.34	-46.32	-108.64	15.99	0.02	493.53	539.86	TRUE	FALSE
**Q86WJ1**	CHD1L	0.41	47.64	-1.63	96.91	0.15	495.46	447.82	TRUE	FALSE
**Q13263**	KRIP-1	0.24	1036.29	-760.4	2832.98	0.05	3593.73	2557.44	FALSE	FALSE
**Q15124**	PGM5	0.5	782.05	104.75	1459.34	0.03	5358.78	4576.73	TRUE	FALSE
**Q9UBZ4**	APEX2	0.3	32.26	-13.29	77.82	0.32	540.17	507.91	TRUE	FALSE
**Q5TDH0**	DDI2	-0.62	-3210.25	-5582.4	-838.09	0.02	10664.55	13874.8	TRUE	FALSE
**Q8TD55**	PKHO2	-0.16	-227.87	-862.34	406.6	0.65	3028.07	3255.94	TRUE	FALSE
**O60784**	TOM1	-0.54	-27.89	-51.69	-4.09	0.03	283.11	311	TRUE	FALSE
**P05231**	IL-6	-0.12	-4.61	-22.39	13.16	0.32	534.12	456.82	TRUE	FALSE
**O14678**	ABCD4	0.1	95.75	-290.89	482.4	0.05	703.01	607.26	TRUE	FALSE
**Q92608**	DOCK2	-0.52	-58.42	-108.17	-8.68	0.02	1522.92	1581.35	TRUE	FALSE
**Q05066**	SRY	-0.33	-24.46	-58.15	9.23	0.24	464.16	488.62	TRUE	FALSE
**P21860**	ERBB3	-0.44	-301.97	-604.25	0.31	0.08	4172.89	4474.86	TRUE	FALSE
**Q16288**	TrkC	0.2	155.82	-181.64	493.29	0.23	3872.15	3716.32	TRUE	FALSE
**P06732**	CK-MM	-0.34	-111.27	-261.31	38.77	0.11	654.49	765.76	TRUE	FALSE
**P14210**	HGF	0.37	226.46	-32.44	485.37	0.17	1527.04	1300.58	TRUE	FALSE
**Q16674**	MIA	-0.39	-171.89	-366.92	23.14	0.04	2078.81	2250.7	TRUE	FALSE
**P14555**	NPS-PLA2	0.43	640.34	22.59	1258.09	0.05	2074.55	1434.21	TRUE	FALSE
**Q12904**	EMAP-2	-0.47	-204.82	-398.87	-10.77	0.09	2329.31	2534.13	TRUE	FALSE
**P24821**	Tenascin	0.17	41.74	-61.59	145.07	0.01	1450.11	1258.41	TRUE	FALSE
**O75173**	ADAMTS-4	0.08	1.18	-5.24	7.6	0.64	167.78	166.6	TRUE	FALSE
**O00253**	ART	-0.44	-76.02	-153.8	1.76	0.12	993.5	1069.52	TRUE	FALSE
**O43278**	HAI-1	-0.48	-613.8	-1182.28	-45.33	0.03	4441.3	5055.11	TRUE	FALSE
**Q9Y5K2**	Kallikrein 4	0.37	18.74	-3.4	40.87	0.04	348.13	329.4	TRUE	FALSE
**Q9ULZ9**	MMP-17	0.21	49.18	-52.51	150.88	0.63	1045.22	996.03	TRUE	FALSE
**O43291**	SPINT2	-0.21	-30.32	-94.89	34.25	0.35	678.88	709.2	TRUE	FALSE

**P55008**	AIF1	-0.4	-1166.44	-2490.78	157.91	0.16	10591.8	11758.23	TRUE	FALSE
**P55773**	MPIF-1	-0.16	-61.19	-234.94	112.55	0.36	30971.43	33614.24	TRUE	FALSE
**P55773**	Ck-b-8-1	-0.16	-61.19	-234.94	112.55	0.36	30971.43	33614.24	TRUE	FALSE
**Q9UNE0**	EDAR	-0.35	-94.75	-215.95	26.44	0.2	816.65	911.4	TRUE	FALSE
**Q9H2X3**	DC-SIGNR	0.52	179.23	27.93	330.54	0.04	3660.73	3481.5	TRUE	FALSE
**P21246**	PTN	0.29	5359.42	-2907.71	13626.55	0.08	37226.78	31867.36	TRUE	FALSE
**P04275**	vWF	0.5	4103.29	561.87	7644.7	0.02	26800.33	22697.04	TRUE	FALSE
**P07858**	Cathepsin B	0.14	47.86	-99.56	195.28	0.09	2132.19	1895.98	TRUE	FALSE
**O95998**	IL-18 BPa	0.18	192.87	-268.72	654.46	0.72	4386.69	4193.82	TRUE	FALSE
**P00742**	Coagulation Factor Xa	-0.12	-89.37	-415.16	236.42	0.42	5580.98	5674.66	TRUE	FALSE
**P00742**	Coagulation Factor X	-0.12	-89.37	-415.16	236.42	0.42	5580.98	5674.66	TRUE	FALSE
**Q9HAV5**	XEDAR	0.45	174.07	13.34	334.81	0.04	864.2	690.13	TRUE	FALSE
**P35475**	IDUA	0.37	148.24	-25.33	321.81	0.09	1460.31	1312.07	TRUE	FALSE
**Q9NQ76**	MEPE	-0.15	-8.54	-34.28	17.2	0.04	152.17	160.71	FALSE	FALSE
**Q9NR71**	ASAH2	-0.26	-151.55	-414.16	111.05	0.33	1676.57	1828.13	TRUE	FALSE
**Q14563**	Semaphorin 3A	-0.39	-30.27	-65.25	4.71	0.12	658.41	688.68	TRUE	FALSE
**P00736**	C1r	-0.47	-10337.57	-20129.59	-545.56	0.02	109867.32	120204.89	TRUE	FALSE
**P0CG37**	CFC1	0.11	35.97	-102.68	174.62	0.43	1347.58	1311.61	TRUE	FALSE
**O75015**	FCG3B	0.42	405.22	-15.73	826.17	0.03	3558.29	3153.07	TRUE	FALSE
**P56159**	GFRa-1	0.48	106.39	12.27	200.51	0.05	947.49	841.1	TRUE	FALSE
**Q16270**	IGFBP-7	0.32	1686.58	-570.84	3944	0.46	33284.76	31598.18	TRUE	FALSE
**Q13007**	IL24	0.3	18.89	-8.52	46.3	0.23	432.56	413.67	TRUE	FALSE
**Q6UXM1**	LRIG3	-0.59	-764.64	-1336.3	-192.99	0.03	6837.18	7601.82	TRUE	FALSE
**P13497**	BMP-1	-0.32	-925.04	-2235.45	385.36	0.28	10893.33	11818.37	TRUE	FALSE
**Q9BU40**	CRDL1	0.45	305.23	10.85	599.61	0.05	2262.78	1957.56	TRUE	FALSE
**P20718**	Granzyme H	0.29	33.12	-14.5	80.73	0.36	396.94	363.82	TRUE	FALSE
**P49862**	Kallikrein 7	-0.57	-223.12	-407.34	-38.9	0.01	878.21	1101.33	TRUE	FALSE
**O94907**	1,00 DKK	0.22	703.71	-626	2033.42	0.84	7286.53	6582.82	TRUE	FALSE
**P01009**	a1-Antitrypsin	0.37	3190.83	-594.11	6975.77	0.04	36040.56	32849.73	TRUE	FALSE
**P15586**	GNS	0.03	16.07	-242.89	275.03	0.19	1177.8	1030.8	TRUE	FALSE
**Q99538**	LGMN	0.27	74.04	-45.39	193.47	0.12	2642.93	2209.72	TRUE	FALSE
**P35968**	VEGF sR2	-0.69	-573.76	-944.28	-203.25	0	5359.31	5933.08	TRUE	TRUE
**P43489**	TNR4	0.52	118.79	23.12	214.46	0.03	772.97	654.19	TRUE	FALSE
**Q13443**	ADAM 9	0.34	389.34	-91.31	869.98	0.05	1425.67	1036.34	TRUE	FALSE
**Q16790**	Carbonic anhydrase 9	0.22	20.71	-19.51	60.94	0.41	437.64	416.93	TRUE	FALSE
**P51665**	PSD7	0.25	19.25	-13.8	52.29	0.23	640.71	621.46	TRUE	FALSE

**P16109**	P-Selectin	0.31	2020	-827.75	4867.76	0.31	25979.66	23959.66	TRUE	FALSE
**P00749**	uPA	0.32	150.74	-55.11	356.59	0.15	2129.42	1978.68	TRUE	FALSE
**Q9HCB6**	Spondin-1	0.43	93.31	-1.44	188.07	0.05	1167.51	1074.19	TRUE	FALSE
**P05362**	sICAM-1	0.59	379.86	96.57	663.14	0.02	2035.01	1655.15	TRUE	FALSE
**Q8IZJ0**	IFN-lambda 2	0.38	20.48	-2.87	43.83	0.08	350.73	330.24	TRUE	FALSE
**Q9GZX3**	CHST6	0.28	17.11	-9.27	43.5	0.83	431.29	414.17	TRUE	FALSE
**O75356**	ENTP5	-0.35	-311.37	-695.9	73.16	0.12	4457	4768.37	TRUE	FALSE
**Q08188**	TGM3	-0.35	-401.9	-911.98	108.19	0.03	592.54	994.44	TRUE	FALSE
**Q4KMG0**	CDON	-0.61	-796.01	-1366.81	-225.21	0.01	5992.99	6789	TRUE	FALSE
**P10909**	Clusterin	-0.4	-746.54	-1572.26	79.17	0.09	17241.82	17988.36	TRUE	FALSE
**P18509**	PACAP-38	0.04	5.89	-56.51	68.28	0.05	184.17	147.21	TRUE	FALSE
**P18509**	PACA	0.04	5.89	-56.51	68.28	0.05	184.17	147.21	TRUE	FALSE
**P18509**	PACAP-27	0.04	5.89	-56.51	68.28	0.05	184.17	147.21	TRUE	FALSE
**P31937**	3HIDH	-0.26	-1681.23	-4830.57	1468.11	0.68	572.65	2253.88	TRUE	FALSE
**P01374**	TNF-b	0.26	120.51	-69.59	310.62	0.02	197.6	213.18	FALSE	FALSE
**P23284**	PPIB	0.1	23.29	-76.83	123.41	0.93	1839.4	1816.11	TRUE	FALSE
**P31947**	STRATIFIN	0.39	689.61	-46.13	1425.35	0.14	4736.84	4047.23	TRUE	FALSE
**P29317**	Epithelial cell kinase	0.33	78.35	-24.01	180.7	0.12	1123.69	1045.34	TRUE	FALSE
**Q13873**	BMP RII	0.25	83.2	-52.34	218.75	0.42	606.14	522.93	TRUE	FALSE
**P52823**	Stanniocalcin-1	0.28	860.13	-395.53	2115.78	0.02	2484.32	1624.19	FALSE	FALSE
**Q07021**	C1QBP	-0.28	-48.52	-124.19	27.16	0.08	2379.86	2428.37	TRUE	FALSE
**P28799**	GRN	0.46	1104.1	37.96	2170.25	0.04	13597.42	12493.32	TRUE	FALSE
**P04196**	HRG	-0.71	-998.13	-1619.11	-377.15	0	5709.37	6707.5	TRUE	TRUE
**Q08380**	LG3BP	0.52	1731.32	275.54	3187.1	0.01	9705.94	7974.62	TRUE	FALSE
**P00568**	Myokinase, human	-0.33	-2677.37	-6362.17	1007.44	0.24	21165.08	23842.45	TRUE	FALSE
**P16333**	NCK1	-0.47	-114.53	-231.13	2.07	0.14	640.83	755.36	TRUE	FALSE
**P30566**	PUR8	-0.5	-1675.91	-3172.05	-179.77	0.01	4972.42	6648.33	TRUE	FALSE
**Q15762**	CD226	0.46	86.5	7.33	165.66	0.2	1353.59	1267.09	TRUE	FALSE
**P16471**	Prolactin Receptor	0.13	31.11	-78.86	141.08	0.03	1014.15	1065.53	TRUE	FALSE
**P04035**	HMGR	0.34	39.4	-8.34	87.14	0.03	311.98	272.58	TRUE	FALSE
**P20393**	NR1D1	0.46	25.71	1.73	49.7	0.03	522.83	497.12	TRUE	FALSE
**P45985**	MP2K4	-0.42	-3005.63	-6136.26	124.99	0.07	22088.4	25094.04	TRUE	FALSE
**O00408**	cGMP-stimulated PDE	0.4	21.55	-1.71	44.81	0.08	466.48	444.93	TRUE	FALSE
**Q14432**	PDE3A	0.31	70.25	-23.82	164.31	0.26	545.85	475.6	TRUE	FALSE
**P45379**	Troponin T	-0.27	-115.19	-317.45	87.07	0.81	1064.95	1180.14	TRUE	FALSE
**P05783**	Keratin 18	-0.32	-30.96	-76.96	15.04	0.29	302.04	333	TRUE	FALSE

**P11309**	PIM1	0.23	36.74	-31.99	105.47	0.41	1146.02	1109.28	TRUE	FALSE
**Q01105**	SET	-0.49	-1011.52	-1940.08	-82.96	0.04	12451.12	13462.63	TRUE	FALSE
**P26842**	CD27	-0.46	-481.99	-975.14	11.15	0	3123.69	3605.68	TRUE	FALSE
**P01160**	ANP	0.24	59.04	-47.87	165.96	0.36	1242.87	1183.82	TRUE	FALSE
**Q13740**	ALCAM	0.25	416.15	-302.09	1134.39	0.41	10458.24	10042.09	TRUE	FALSE
**O60243**	H6ST1	-0.3	-43.4	-108.22	21.42	0.26	1004.1	1047.51	TRUE	FALSE
**P61247**	RS3A	0.34	25.61	-6.43	57.65	0.48	551.43	525.82	TRUE	FALSE
**O14763**	TRAIL R2:ECD	0.37	26.45	-3.84	56.73	0.26	474.21	447.77	TRUE	FALSE
**O14763**	TRAIL R2	0.37	26.45	-3.84	56.73	0.26	474.21	447.77	TRUE	FALSE
**O14763**	TRAIL R2:Death	0.37	26.45	-3.84	56.73	0.26	474.21	447.77	TRUE	FALSE
**Q9H772**	GREM2	0.35	310.25	-76.33	696.83	0.18	4795.06	4484.81	TRUE	FALSE
**Q8NBM8**	PCYXL	0.38	110.59	-17.9	239.09	0.03	2009.28	1898.69	FALSE	FALSE
**Q5GAN6**	RNS10	0.1	30.03	-96.82	156.88	0.35	772.07	742.04	TRUE	FALSE
**Q00888**	PSG4	0.47	69.96	6.89	133.04	0.13	525.97	456	TRUE	FALSE
**A4D1T9**	PRS37	0.14	9.24	-18.67	37.16	0.2	172.04	162.8	TRUE	FALSE
**P05160**	coagulation factor XIII B	-0.29	-1020.41	-2618.6	577.78	0.13	26493.11	27513.52	TRUE	FALSE
**Q9NPH6**	OBP2B	-0.16	-80.27	-301.58	141.04	0.03	784.99	865.26	FALSE	FALSE
**Q9NTU7**	CBLN4	-0.59	-263.61	-460.77	-66.46	0.01	1601.49	1865.11	TRUE	FALSE
**Q99784**	NOE1	0.31	73.42	-24.89	171.73	0.05	256.33	182.91	FALSE	FALSE
**P51124**	Granzyme M	0.48	57.24	7.88	106.6	0.01	498.39	441.15	TRUE	FALSE
**Q6UWP8**	SBSN	0.11	56.8	-157	270.61	0.02	479.46	422.66	FALSE	FALSE
**Q5T2D2**	TRML2	-0.36	-316.92	-706.13	72.3	0.03	3121.35	3438.26	FALSE	FALSE
**Q9BUN1**	CA056	-0.37	-170.52	-375.44	34.4	0.2	1429.6	1600.12	TRUE	FALSE
**P13385**	Cripto	-0.49	-470.32	-898.65	-41.99	0.01	900.34	1370.66	TRUE	FALSE
**P42702**	LIF sR	-0.38	-114.44	-249.15	20.27	0.14	1709	1823.44	TRUE	FALSE
**Q13561**	Dynactin subunit 2	-0.69	-97.5	-159.64	-35.36	0	1165.15	1262.65	TRUE	TRUE
**P30042**	ES1	-0.35	-8.7	-19.66	2.26	0.03	161.49	170.19	TRUE	FALSE
**O95157**	NXPH3	-0.53	-155.94	-287.08	-24.79	0.02	1881.94	2037.88	TRUE	FALSE
**Q9NZK5**	CECR1	0.44	2644.46	18.69	5270.24	0.06	14803.19	12158.73	TRUE	FALSE
**Q9UNI1**	ELA1	0.35	2837.43	-704.36	6379.22	0.05	27987.31	25149.88	TRUE	FALSE
**Q9UQ74**	PSG8	0.36	50.25	-7.6	108.1	0.08	501.51	451.26	TRUE	FALSE
**Q9BXJ1**	C1QT1	0.46	469.98	38.94	901.02	0.06	4817.57	4347.59	TRUE	FALSE
**Q9Y5H3**	PCDGA	0.15	40.27	-86.77	167.32	0.02	1012.43	972.15	FALSE	FALSE
**Q92874**	DNSL2	0.54	246.61	50.19	443.02	0.04	2625.94	2379.33	TRUE	FALSE
**Q6UXI9**	Nephronectin	0.28	57.63	-31.5	146.75	0.39	1028.15	970.53	TRUE	FALSE
**Q86SI9**	CEI	-0.42	-679.64	-1381.42	22.14	0.07	4880.61	5560.25	TRUE	FALSE
**O43897**	TLL1	-0.01	-26.08	-1861.66	1809.5	0.02	7594.64	7620.72	FALSE	FALSE
**P22792**	CPN2	-0.21	-800.08	-2492.84	892.67	0.16	20455.95	21256.04	TRUE	FALSE

**Q00889**	PSG6	0	0.25	-24.55	25.05	0.45	623.06	622.81	TRUE	FALSE
**Q6DKI7**	PVRIG	-0.18	-28.35	-96.74	40.05	0.28	1471.62	1499.97	TRUE	FALSE
**Q9UJZ1**	Stomatin-like protein 2	0.13	56.61	-118.93	232.16	0.02	942.23	885.62	FALSE	FALSE
**Q5TAT6**	CODA1	-0.52	-137.3	-255.79	-18.81	0.05	1190.25	1327.55	TRUE	FALSE
**P23327**	SRCH	0.35	60.57	-11.78	132.92	0.7	235.02	174.45	TRUE	FALSE
**Q14435**	GALT3	-0.11	-21.05	-111.32	69.22	0.33	531.85	552.9	TRUE	FALSE
**Q6DN72**	FCRL6	-0.32	-105.96	-262.4	50.47	0.23	1400.03	1505.99	TRUE	FALSE
**P10696**	PPBN	-0.12	-53.76	-260.73	153.21	0.82	324.45	335.08	TRUE	FALSE
**P02786**	TR:CD	0.13	83.61	-194	361.21	0.21	4502.05	3997.54	TRUE	FALSE
**P02786**	TR:ECD	0.13	83.61	-194	361.21	0.21	4502.05	3997.54	TRUE	FALSE
**O94766**	B3GA3	-0.23	-874.67	-2694.12	944.77	0.76	442.79	1317.46	TRUE	FALSE
**Q6UWJ8**	C16L2	-0.49	-49.67	-96.58	-2.75	0.07	344.75	394.42	TRUE	FALSE
**Q8NBK3**	SUMF1	-0.49	-145.23	-278.12	-12.34	0.06	1361.18	1506.41	TRUE	FALSE
**Q92562**	FIG4	0.18	760.31	-997.67	2518.29	0.02	1587.89	827.58	FALSE	FALSE
**Q9BXS0**	COPA1	-0.26	-6.32	-17.42	4.77	0.61	166.27	172.59	TRUE	FALSE
**P58658**	F176C:CD	0.45	2722.47	78.43	5366.51	0.02	24416.47	21694	TRUE	FALSE
**P58658**	F176C:ECD	0.45	2722.47	78.43	5366.51	0.02	24416.47	21694	TRUE	FALSE
**P21854**	CD72	0.03	5.05	-76.06	86.16	0.53	1057.77	920.34	TRUE	FALSE
**O75023**	LIRB5	0.04	165.63	-1799.97	2131.23	0.84	11940.92	11775.29	TRUE	FALSE
**O75326**	Semaphorin-7A	0.22	163.72	-150.58	478.02	0.87	1818.13	1654.42	TRUE	FALSE
**Q8N807**	PDILT	0.36	23.36	-4.59	51.3	0.35	623.57	600.21	TRUE	FALSE
**Q8N475**	FSTL5	-0.33	-15.92	-37.17	5.34	0.04	400.24	416.16	TRUE	FALSE
**Q9NTK1**	DEPP	-0.71	-938.68	-1531.05	-346.3	0	3202.58	4141.25	TRUE	TRUE
**Q6UXH0**	TD26	0.46	38.72	3.66	73.79	0.09	489.28	450.56	TRUE	FALSE
**P14415**	AT1B2	-0.5	-511.71	-972.57	-50.85	0.03	3530.35	4042.06	TRUE	FALSE
**Q99584**	S100A13	0.61	870.87	244.01	1497.73	0	3487.22	2616.35	TRUE	FALSE
**A6NI73**	LIRA5	0.6	218.53	64.54	372.52	0.02	1879.53	1661	TRUE	FALSE
**Q8N109**	KI2LA	0.16	49.25	-80.89	179.39	0.02	879.01	505.36	TRUE	FALSE
**Q9UQ72**	PSG11	-0.36	-33.42	-74.91	8.08	0.24	729.02	762.44	TRUE	FALSE
**Q24JP5**	T132A	0.19	86.96	-107.63	281.55	0.66	1809.47	1722.51	TRUE	FALSE
**Q8N5Y8**	PAR16	0.47	95.79	11.64	179.95	0.83	569.9	474.11	TRUE	FALSE
**Q9Y2R0**	CCD56	0.25	124.07	-83.59	331.73	0.64	2234.11	2110.04	TRUE	FALSE
**P19224**	UGT 1A6	0.27	322.29	-186.97	831.55	0.7	1119.9	797.61	TRUE	FALSE
**Q8TC05**	MDM1	0.47	54.39	4.53	104.24	0.04	986.45	932.07	TRUE	FALSE
**Q9BVH7**	SIA7E	0.31	31.59	-11.1	74.28	0.64	376.41	344.82	TRUE	FALSE
**O60507**	TPST1	-0.32	-303.61	-723.66	116.43	0.18	3983.47	4287.09	TRUE	FALSE
**Q8NBV8**	SYT8	0.3	166.41	-62.13	394.94	0.71	384.72	218.31	TRUE	FALSE
**O95461**	LARGE	0.35	85.84	-20.17	191.86	0.12	1055.12	969.27	TRUE	FALSE

**Q86UP6**	CUZD1	-0.5	-94.91	-185.49	-4.32	0.03	284.02	378.93	TRUE	FALSE
**P19827**	ITI heavy chain H1	-0.66	-3764.09	-6264.88	-1263.29	0	39492.08	43256.16	TRUE	TRUE
**Q9BYC8**	RM32	0.2	23.57	-27.34	74.47	0.03	442.46	418.89	TRUE	FALSE
**Q9H3S3**	Spinesin	0.24	19.91	-15.48	55.3	0.93	421.5	401.59	TRUE	FALSE
**P84157**	MXRA7	0.34	97.69	-22.52	217.91	0.12	999.4	901.71	TRUE	FALSE
**Q96CU9**	FXRD1	-0.48	-79.59	-154.89	-4.29	0.07	682.84	762.43	TRUE	FALSE
**P15151**	Poliovirus receptor	-0.14	-193.54	-850.28	463.2	0.64	752.08	945.62	TRUE	FALSE
**P0C8F1**	PATE4	0.23	12.56	-9.95	35.08	0.9	223.94	211.38	TRUE	FALSE
**Q9Y6H6**	KCNE3	0.26	33.44	-19.44	86.31	0.7	289.18	255.74	TRUE	FALSE
**Q1L6U9**	PSMP	-0.59	-213.03	-377.4	-48.66	0.03	952.51	1165.54	TRUE	FALSE
**O15400**	Syntaxin-7	0.34	2327.04	-670.9	5324.98	0.25	15529.57	13202.53	TRUE	FALSE
**O00300**	OPG	0.44	933.25	0.05	1866.46	0.04	7666.62	6733.37	TRUE	FALSE
**Q6IA17**	SIGIRR	0.21	44.58	-48.43	137.58	0.1	856.05	811.48	TRUE	FALSE
**A8MWY0**	K132L	0.38	17.63	-2.11	37.38	0.13	415.83	398.2	TRUE	FALSE
**Q86XK7**	VSIG1	0.42	31.84	0.36	63.32	0.02	205.84	174	TRUE	FALSE
**P08138**	NGF R	-0.4	-1068.71	-2358.4	220.99	0.03	1302.46	2371.17	TRUE	FALSE
**P01229**	LSHB	0.42	44.6	-0.49	89.69	0.07	476.2	431.6	TRUE	FALSE
**Q9UBX1**	CATF	0.29	11.94	-6.41	30.28	0.26	1660.99	1468.89	TRUE	FALSE
**Q2I0M5**	RSPO4	0.3	47.97	-19.73	115.67	0.09	445.77	397.8	TRUE	FALSE
**Q12805**	FBLN3	0.47	301.32	30.36	572.28	0.02	2749.39	2448.07	TRUE	FALSE
**Q8NCW6**	GLT11	0.25	33.47	-23.94	90.88	0.37	1291.97	1258.49	TRUE	FALSE
**P18206**	Vinculin	0.49	178.75	24.42	333.09	0.01	794.55	615.8	TRUE	FALSE
**Q8N5W8**	FA24B	0.33	30.18	-8.5	68.85	0.76	420.58	390.4	TRUE	FALSE
**Q9P2E7**	PCD10:CD	-0.03	-51.66	-764.85	661.52	0.15	1536.5	1373.54	TRUE	FALSE
**Q9P2E7**	PCD10:ECD	-0.03	-51.66	-764.85	661.52	0.15	1536.5	1373.54	TRUE	FALSE
**Q9P218**	COKA1	-0.49	-11.49	-22.77	-0.2	0.21	70.4	81.88	TRUE	FALSE
**P60059**	SC61G	0.27	15.07	-10.21	40.35	0.04	399.25	384.17	TRUE	FALSE
**Q86U42**	PABP2	0.22	84.5	-78.14	247.13	0.45	1206.11	1121.62	TRUE	FALSE
**O75354**	ENTP6	-0.53	-40.07	-74.18	-5.95	0.02	839.91	879.98	TRUE	FALSE
**Q6UVK1**	CSPG4	-0.5	-57.9	-112.07	-3.73	0.01	502.88	560.78	TRUE	FALSE
**Q8IVH8**	M4K3	0.28	10.02	-5.54	25.58	0.15	338.38	328.36	TRUE	FALSE
**Q6Q8B3**	MO2R2	0.42	72.96	1.04	144.87	0.35	480.37	407.41	TRUE	FALSE
**P54709**	AT1B3	0.07	486.26	-2377.76	3350.28	0.04	8197.06	7710.81	FALSE	FALSE
**O94906**	PRP6:region 1	-0.06	-4.59	-34.83	25.64	0.04	726.93	808.72	TRUE	FALSE
**O94906**	PRP6:region 2	-0.06	-4.59	-34.83	25.64	0.04	726.93	808.72	TRUE	FALSE
**Q96D42**	TIM-1	0.29	587.97	-247.87	1423.81	0.09	1657.52	1069.55	TRUE	FALSE
**Q8NFZ4**	NLGN2:CD	-0.18	-48.37	-180.77	84.03	0.04	246.29	262.07	FALSE	FALSE

**Q8NFZ4**	NLGN2:ECD	-0.18	-48.37	-180.77	84.03	0.04	246.29	262.07	FALSE	FALSE
**P52943**	CRIP2	0.42	121.5	-3.55	246.55	0.11	1633.88	1512.38	TRUE	FALSE
**Q5XG99**	LYSM4	0.3	38.6	-14.29	91.5	0.45	314.49	275.88	TRUE	FALSE
**Q5DID0**	UROL1	0.38	29.32	-2.75	61.39	0.47	459.61	430.29	TRUE	FALSE
**P04155**	TFF1	0.37	513.42	-75.11	1101.95	0.23	2868.51	2355.09	TRUE	FALSE
**P60604**	UB2G2	-0.47	-108.58	-211.45	-5.7	0.04	1573.82	1682.4	TRUE	FALSE
**P50897**	PPT1	0.33	68.75	-22.19	159.69	0.33	999.7	930.95	TRUE	FALSE
**P16562**	CRIS2	-0.74	-381.2	-619.63	-142.77	0	1056.16	1437.36	TRUE	TRUE
**Q8TAE8**	G45IP	-0.16	-4.98	-18.31	8.35	0.49	273.43	278.41	TRUE	FALSE
**O60487**	MPZL2	-0.43	-112.81	-230.77	5.14	0.06	1110.68	1223.5	TRUE	FALSE
**Q96HG1**	CX069	0.26	46.76	-29.12	122.65	0.52	731.54	684.78	TRUE	FALSE
**O95156**	NXPH2	-0.25	-86.23	-236.73	64.26	0.23	828.55	914.79	TRUE	FALSE
**O00453**	LST1	0.4	231.29	-14.34	476.93	0.12	2195.55	1964.26	TRUE	FALSE
**Q13753**	Laminin gamma-2	0.45	609.45	44.54	1174.37	0.03	2724.49	2115.03	TRUE	FALSE
**Q96PZ7**	CSMD1	-0.4	-62.78	-134.27	8.71	0.21	740.72	803.51	TRUE	FALSE
**P43246**	MSH2	-0.47	-176.95	-353.47	-0.43	0.07	577.61	754.56	TRUE	FALSE
**Q8TDY8**	IGDC4	-0.53	-92.75	-170.65	-14.85	0.1	708.94	801.69	TRUE	FALSE
**Q9UKW4**	VAV3	-0.31	-82.62	-199.23	33.99	0.11	1381.66	1464.28	TRUE	FALSE
**P47813**	IF1AX	0.28	34.1	-17.65	85.86	0.53	660.86	626.75	TRUE	FALSE
**P48775**	T23O	-0.36	-269.31	-593.75	55.13	0.1	3031.69	3300.99	TRUE	FALSE
**Q8TED9**	AF1L1	0.2	106.2	-112.73	325.12	0.33	309.19	202.99	TRUE	FALSE
**P55316**	FOXGB	-0.38	-17.89	-38.52	2.74	0.16	145.7	163.59	TRUE	FALSE
**O75829**	LECT1	-0.28	-82.94	-211.69	45.82	0.18	2161.76	2244.7	TRUE	FALSE
**P04264**	Keratin-1	0.54	295.52	64.8	526.24	0.1	1660.37	1364.85	TRUE	FALSE
**Q9UN67**	PCDBA	-0.56	-147.88	-269.36	-26.41	0.01	1171.73	1319.61	TRUE	FALSE
**Q8N3Z0**	PRS35	-0.33	-67.38	-165.37	30.6	0.04	501.12	568.51	TRUE	FALSE

Table S2. KEGG database pathway enrichment analysis for all the proteins associated to diagnosis (651 proteins). N Genes: Number of genes. FDR: False discovery rate. Fold enrichment is calculated based on the observed number of genes in the pathway divided by the expected number of genes in the pathway[1]. The enrichment FDR is calculated based on the Benjamini-Hochberg correction of the Fisher's exact test[1].

Enrichment FDR	N Genes	Pathway Genes	Fold Enrichment	Pathway	Genes
2.51085581412774e-06	27	294	3.64811862244898	Path:hsa04060 Cytokine-cytokine receptor interaction	EDAR IL24 IL17F IL17RE CSF1 GDF7 GDF10 IL<br>L2 IFNA4 IL5 IL6 IL6R TNFRSF9 INHBA LIFR<br>NFRSF11B IL22 PRLR EDA2R BMPR1B BMPR2<br>4 IL1R2 TNFRSF10B CD27
0.000191745669841688	26	354	2.9175788606403	Path:hsa04151 PI3K-Akt signaling pathway	CDK2 COL6A1 CSF1 CREB3L4 EFNA3 EGFR EP<br>BB3 VEGFD FLT4 HGF TNC IFNA4 IL6 IL6R IT<br>DR LAMC2 MTCP1 MYC NGFR PRLR SGK1 CR<br>SPP1 VWF
0.000343437089581484	18	200	3.57515625	Path:hsa04510 Focal adhesion	VAV3 COL6A1 EGFR ELK1 VEGFD FLT4 MYLP<br>TNC ITGA2 KDR LAMC2 MYL7 SPP1 BRAF VC<br>CAPN2
0.00210273506143515	15	170	3.50505514705882	Path:hsa04310 Wnt signaling pathway	FZD10 CSNK2A2 DKK1 DKK4 DKK2 RSPO1 RS<br>C SOST PPP3R1 LGR4 SFRP5 WNT5A RSPO3 L
0.00242140654688771	11	99	4.41377314814815	Path:hsa04640 Hematopoietic cell lineage	CR2 CSF1 IL5 IL6 IL6R ITGA2 TFRC IL1R2 CD<br>A CD9
0.00267867379709375	64	1538	1.6530125704378	Path:hsa01100 Metabolic pathways	COQ7 B3GALT5 HIBADH SAT2 CKM HYKK CO<br>P2 ADSL DGKB DLD AGXT AK1 MLYCD ALPI<br>GALNT3 AOC1 B3GAT3 GNS PGP GSTP1 HAG<br>HMGCL HMGCR MMAB IDUA ALDH6A1 NAG<br>1 PAFAH1B3 PDE2A PDE3A ATP5PB UPB1 AD<br>2G2A PLCD1 UGT1A6 PNPO PGM2 PPT1 KYAT<br>H2 KMT2C RPE GALNT11 BLVRB NMNAT1 BP<br>D CA9 ST6GALNAC5 POMK B3GALT2 CBR1 ST<br>IDI2 LARGE1 ENTPD6 ENTPD5 FIG4 XYLB
0.0026922193720459	20	294	2.70231009070295	Path:hsa04010 MAPK signaling pathway	CSF1 EFNA3 EGFR EPHA2 ELK1 ERBB3 VEGFD<br>HGF HSPB1 KDR MAP3K11 MYC NGFR PPP3R<br>K6 PTPN7 MAP2K4 BRAF MAP4K3
0.00778122472561134	9	84	4.25613839285714	Path:hsa04012 ErbB signaling pathway	EGFR ELK1 ERBB3 NRG1 MYC NCK1 MAP2K4<br>BTC
0.00778122472561134	13	166	3.11091239959839	Path:hsa04022 cGMP-PKG signaling pathway	CREB3L4 KNG1 ATP1B2 ATP1B3 NPPA NPPB N<br>TP2A3 PDE2A PDE3A PPP3R1 PRKG1 CREB3L2
0.00778122472561134	14	181	3.07257136279926	Path:hsa04360 Axon guidance	SEMA3A CFL2 GDF7 EFNA3 EPHA2 EPHA6 NC<br>3R1 SEMA4G BMPR1B SLIT3 BMPR2 WNT5A S
0.00778122472561134	9	85	4.20606617647059	Path:hsa04610 Complement and coagulation cascades	CLU CR2 F10 F13B KNG1 PLAU SERPING1 C1C<br>F
0.00778122472561134	10	103	3.85669498381877	Path:hsa04974 Protein digestion and absorption	COL6A1 COL10A1 COL13A1 CPA1 CPB1 ATP1B<br>B3 PRSS3 COL20A1 COL25A1
0.0172001314510004	8	79	4.02267932489451	Path:hsa01521 EGFR tyrosine kinase inhibitor resistance	EGFR ERBB3 HGF NRG1 IL6 IL6R KDR BRAF
0.0172001314510004	12	162	2.94251543209877	Path:hsa04630 JAK-STAT signaling pathway	IL24 EGFR IFNL2 IFNA4 IL5 IL6 IL6R LIFR MY<br>PIM1 PRLR
0.0172001314510004	9	97	3.6857280927835	Path:hsa05215 Prostate cancer	CDK2 CREB3L4 EGFR GSTP1 PLAU CREB3L2 S<br>RAF IL1R2
0.0285766206178678	14	221	2.51644984917044	Path:hsa04024 cAMP signaling pathway	VAV3 CGA ADCYAP1 CREB3L4 LHB ATP1B2 A<br>NPY NPPA ATP2A3 PDE3A GHRL CREB3L2 BR
0.0377136593677669	9	112	3.19210379464286	Path:hsa04668 TNF signaling pathway	CSF1 CREB3L4 VEGFD ICAM1 IL6 MAP2K6 MA<br>REB3L2 FADD
0.0398716524197991	23	476	1.91943496148459	Path:hsa05022 Pathways of neurodegeneration-multiple diseases	DCTN2 FZD10 CSF1 CSNK2A2 DKK1 DKK4 DK<br>KLC1 NEFL ATP2A3 ATP5PB PPP3R1 MAP2K6<br>BRAF STX1A UBE2G2 WNT5A CAPN2 FADD U<br>FIG4
0.0416364262884972	13	210	2.45910218253968	Path:hsa04015 Rap1 signaling pathway	VAV3 CSF1 EFNA3 EGFR EPHA2 VEGFD FLT4<br>DR NGFR MAP2K6 RAP1GAP BRAF
0.0416364262884972	11	162	2.6973058127572	Path:hsa05161 Hepatitis B	CDK2 CREB3L4 ELK1 IFNA4 IL6 MYC MAP2K6<br>K4 CREB3L2 BRAF FADD

Table S3. Transcription factors families significantly enriched ($p < 0.05$) according to TRANSFAC and JASPAR databases (only human origin were included). P-values are based on Fisher's exact test and adjusted p-values (q-values) are corrected according to the Benjamini-Hochberg procedure[2].

term	p-value	q-value
NFAT2	0.0001995321074739234	0.06365074228418156
PPARA	0.0009267238837761202	0.14781245946229116
LTF	0.004558679501037194	0.44898873042817905
SMAD4	0.005629952732641744	0.44898873042817905
SREBF2	0.01003370616595746	0.5342158353744388
RUNX1	0.01004794674685465	0.5342158353744388
MIB2	0.012734118011709579	0.580311949390765
CEBPB	0.015326688654753916	0.6111517101083124
ETV4	0.017695930432271197	0.6272224230993901
USF2	0.020521200144212406	0.6546262846003758
PDX1	0.028282582098918877	0.7659509485165976
RBPJ	0.03103722012684079	0.7659509485165976
POU3F1	0.031214301977165418	0.7659509485165976
SND1	0.0405410668359135	0.7875124811922151
FOS	0.04391921013798342	0.7875124811922151
CBEPB	0.044121681497102466	0.7875124811922151
JDP2	0.04757238342557294	0.7987152796188299

Supplementary references

1. Ge SX, Jung D, Yao R. ShinyGO: a graphical gene-set enrichment tool for animals and plants. Valencia A, ed. *Bioinformatics*. 2020;36(8):2628-2629. doi:10.1093/bioinformatics/btz931

2. Gene Set Knowledge Discovery with Enrichr - Xie - 2021 - Current Protocols - Wiley Online Library. Accessed July 10, 2024. https://currentprotocols.onlinelibrary.wiley.com/doi/10.1002/cpz1.90
