# Supplementary material for: Towards precision medicine strategies using plasma proteomic profiling for suspected gallbladder cancer: A pilot study
Source: JHEP Rep. 2025 Feb 21;7(6):101365. doi: 10.1016/j.jhepr.2025.101365 (PMC12135361; doi:10.1016/j.jhepr.2025.101365)
Supplement: Multimedia component 2 [file mmc2.docx]

**JHEP Reports**

**CTAT methods**

Tables for a “Complete, Transparent, Accurate and Timely account” (CTAT) are now mandatory for all revised submissions. The aim is to enhance the reproducibility of methods.

- Only include the parts relevant to your study
- Refer to the CTAT in the main text as ‘Supplementary CTAT Table’
- Do not add subheadings
- Add as many rows as needed to include all information
- Only include one item per row

**If the CTAT form is not relevant to your study, please outline the reasons why:**

|  |
| --- |

- 1. **Antibodies**

| **Name** | **Citation** | **Supplier** | **Cat no.** | **Clone no.** |
| --- | --- | --- | --- | --- |
|  |  |  |  |  |

- 1. **Cell lines**

| **Name** | **Citation** | **Supplier** | **Cat no.** | **Passage no.** | **Authentication test method** |
| --- | --- | --- | --- | --- | --- |
|  |  |  |  |  |  |

- 1. **Organisms**

| **Name** | **Citation** | **Supplier** | **Strain** | **Sex** | **Age** | **Overall n number** |
| --- | --- | --- | --- | --- | --- | --- |
|  |  |  |  |  |  |  |

- 1. **Sequence based reagents**

| **Name** | **Sequence** | **Supplier** |
| --- | --- | --- |
|  |  |  |

- 1. **Biological samples**

| **Description** | **Source** | **Identifier** |
| --- | --- | --- |
| Plasma samples | Human |  |

- 1. **Deposited data**

| **Name of repository** | **Identifier** | **Link** |
| --- | --- | --- |
| Figshare | [m9.figshare.26388166](https://doi.org/10.6084/m9.figshare.26388166) | <https://doi.org/10.6084/m9.figshare.26388166> |

- 1. **Software**

| **Software name** | **Manufacturer** | **Version** |
| --- | --- | --- |
| R Studio | Posit Software | 2024.04.0 |
| ClusterProfiler | https://maayanlab.cloud/Enrichr/ | V 4.8.3 |
| ShinyGO web-tool | http://bioinformatics.sdstate.edu/go/ | V 0.80 |
| Enrichr | https://maayanlab.cloud/Enrichr/ | June 8, 2023 |

- 1. **Other (*e.g*. drugs, proteins, vectors etc.)**

| Proteins | SomaScan 7 000 panel | SomaLogic, Boulder, Colorado, USA |
| --- | --- | --- |
| Bioinformatics pipeline | Developed by the authors | [https://github.com/MedH-AB-group/Cancer_plasma_proteomics](https://github.com/MedH-AB-group/Cancer_serum_proteomics) |

- 1. **Please provide the details of the corresponding methods author for the manuscript:**

| **Ghada Nouairia:** [**ghada.nouairia@ki.se**](mailto:ghada.nouairia@ki.se)**, phone: 0046739944562,**  **adress: Karolinska Universitetssjukhuset Huddinge, C177, 14186 Huddinge** |
| --- |

**2.0 Please confirm for randomised controlled trials all versions of the clinical protocol are included in the submission. These will be published online as supplementary information.**

|  |
| --- |
