# Supplementary material for: Towards precision medicine strategies using plasma proteomic profiling for suspected gallbladder cancer: A pilot study
Source: JHEP Rep. 2025 Feb 21;7(6):101365. doi: 10.1016/j.jhepr.2025.101365 (PMC12135361; doi:10.1016/j.jhepr.2025.101365)
Supplement: Multimedia component 4 [file mmc4.pdf]

JHEP|Reports

Towards precision medicine strategies using plasma proteomic profiling for suspected gallbladder cancer: A pilot study

Authors

Ghada Nouairia, Martin Cornillet, Hannes Jansson, Annika Bergquist, Ernesto Sparrelid

Correspondence

ghada.nouairia@ki.se (G. Nouairia).

Graphical abstract

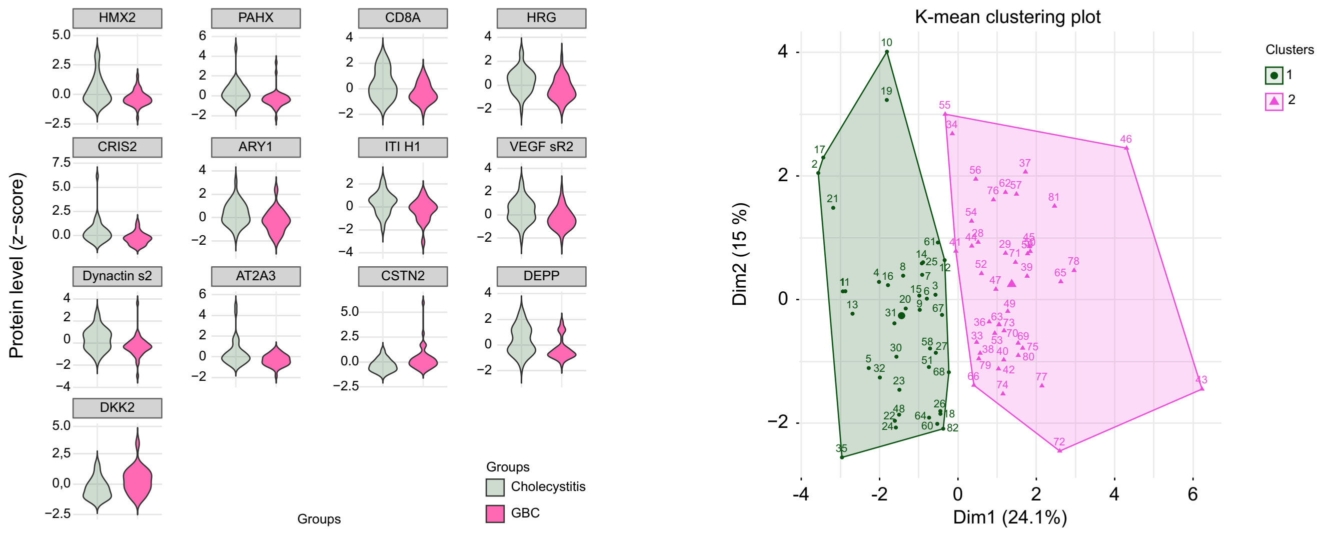

Out of 7,500 plasma proteins screened, we identified 13 proteins (right) that, through machine learning, can preoperatively differentiate gallbladder cancer (n = 40) from cholecystitis (n = 38) (left)

Highlights:

- The plasma proteome can differentiate gallbladder cancer from cholecystitis with high accuracy.
- Machine learning models can identify a biologically relevant subset of differentiating plasma proteins.
- Plasma protein tests could have the potential to assist in preoperative decision-making when GBC is suspected.

Impact and implications:

This study highlights the potential of plasma proteomic profiling to significantly improve the preoperative diagnostic accuracy of gallbladder cancer *vs.* cholecystitis. Using machine learning models, we identified biologically relevant plasma proteins associated with the diagnosis of gall bladder cancer. A noninvasive preoperative test based on selected plasma proteins could potentially enhance clinical decision-making, reduce unnecessary surgeries, and mitigate the associated risks for patients with suspected GBC, marking a step forward in precision medicine.

https://doi.org/10.1016/j.jhepr.2025.101365

© 2025 The Author(s). Published by Elsevier B.V. on behalf of European Association for the Study of the Liver (EASL). This is an open access article under the CC BY license (http://creativecommons.org/licenses/by/4.0/). JHEP Reports, 2025, 7, 1–11

Research article

JHEP|Reports

Towards precision medicine strategies using plasma proteomic profiling for suspected gallbladder cancer: A pilot study

Ghada Nouairia[1,*], Martin Cornillet[2], Hannes Jansson[3,4], Annika Bergquist[1,4], Ernesto Sparrelid[3,4]

JHEP Reports **2025**. vol. 7 | 1–11

Check for updates

Background & Aims: Currently, preoperative diagnostic methods that can distinguish cancer from benign disease of the gallbladder are insufficient, and several surgical resections can be avoided if the pathology is known prior to surgery. This study aimed to assess whether preoperative plasma proteins can distinguish gallbladder cancer (GBC) from cholecystitis, with the main goal of identifying proteins for multivariate description of the postoperative diagnosis, before surgery.

Methods: Samples from 82 individuals with suspected GBC who underwent bisegmentectomy and lymphadenectomy at Karolinska University Hospital between 2009 and 2020 were included in this retrospective, observational, single-center study. Preoperative plasma samples were analyzed using a 7,500 proteomics panel from SomaScan®. High-dimensional statistical methods including machine learning regularization, were used to analyze the data.

Results: In our study, we identified and characterized a panel of 651 proteins that exhibited differential expression between GBC and cholecystitis. Through multivariate analysis, we demonstrated that circulating proteomics data provide valuable insights for diagnosing GBC before surgical intervention. Notably, we identified a subset of eight plasma proteins (PAHX, CD8A, HRG, CRIS2, Dynactin subunit 2, AT2A3, CSTN2, and DEPP) that effectively differentiated GBC from cholecystitis with a diagnostic accuracy of 94% when validated on a test set. These findings hold potential for clinical validation and could significantly aid in preoperative decision-making when GBC is suspected.

Conclusions: Our findings demonstrate that the preoperative assessment of plasma proteins can accurately differentiate cholecystitis from malignancy, supporting the potential development of a noninvasive test to assist preoperative decision-making when GBC is suspected.

© 2025 The Author(s). Published by Elsevier B.V. on behalf of European Association for the Study of the Liver (EASL). This is an open access article under the CC BY license (http://creativecommons.org/licenses/by/4.0/).

Introduction

Gallbladder cancer (GBC) and inflammatory disease of the gallbladder (cholecystitis) are clinical entities that are difficult to separate before histological analysis, especially xanthogranulomatous cholecystitis.[1,2] Despite advances in modern radiology, to date, no diagnostic modality has been sufficiently accurate[3] and differential diagnosis is challenging (Fig. 1).

Biopsies should be avoided preoperatively because of the risk of tumor seeding and spread. Hence, many individuals with radiological suspicion of GBC undergo radical cholecystectomy and lymphadenectomy even if the diagnosis is often uncertain. Radical cholecystectomy generally consists of resection of liver segments 4b and 5 together with gallbladder or wedge resection of the adjacent liver parenchyma surrounding the gallbladder.[4]

When postoperative histology revealed only inflammation in the gallbladder, these patients received an unnecessary invasive treatment, with a substantial risk of complications. If accurate methods to distinguish between cancer and inflammation of the gallbladder before surgery are available, simple cholecystectomy with much lower risks, or even no surgery, could have been preferred for these individuals. Several attempts have been made to improve preoperative diagnostics in this patient group without convincing results.[5–7] To date, clinicians have been faced with the risk of overtreating some patients to avoid missing the optimal treatment window for individuals with early GBC, where delayed diagnosis leads to poorer prognosis.

The use of plasma biomarkers for diagnosis is gaining interest.[8–10] In 2014, Wang *et al.*[8] evaluated the serum levels of CA 242, CA 125, CEA, and CA 19-9, concluding that CA 242 and CA 19-9 might assist in the diagnosis in the early stage and as prognostic markers for GBC compared with benign gallbladder disease.[8] Recently, in 2023, Baichan *et al.*[9] analyzed tissue and blood using liquid chromatography-mass spectrometry and identified nine dysregulated proteins (APOA1, APOA2, RET4, TTR, HEMO, HBB, HBA, PIGR, and APOE) in both tissue and plasma compared with benign biliary

* Corresponding author. Address: Hälsovägen 13, C177, Karolinska University Hospital, Stockholm, 14186, Stockholm, Sweden.
E-mail address: ghada.nouairia@ki.se (G. Nouairia).
https://doi.org/10.1016/j.jhepr.2025.101365

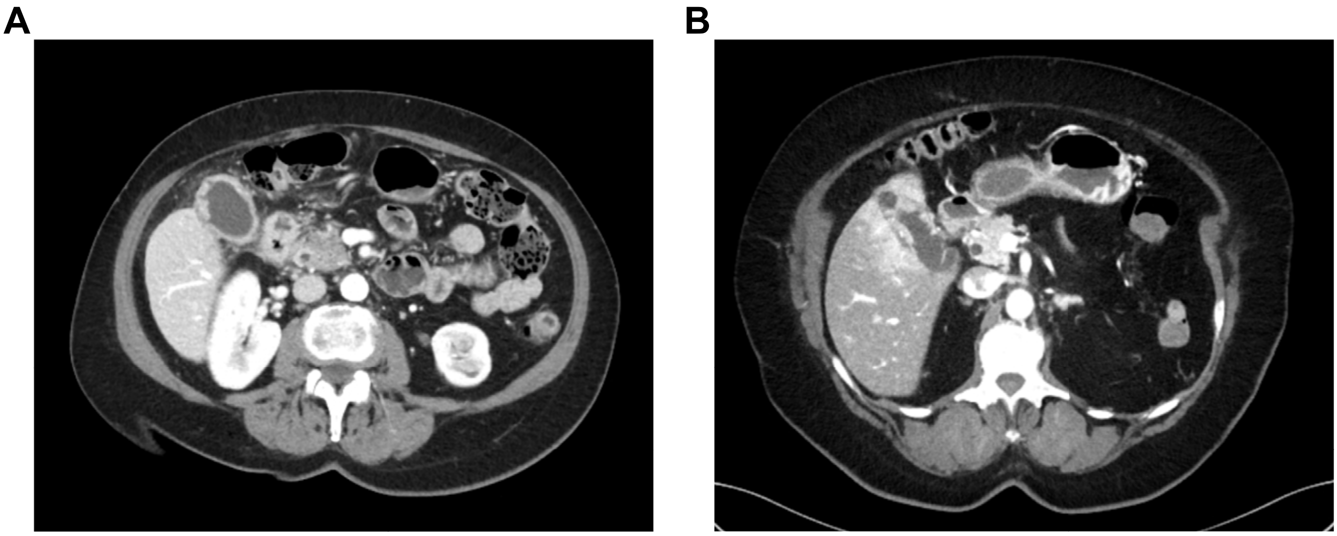

Fig. 1. Demonstrating the preoperative diagnostic challenge in gallbladder cancer (GBC). (A) Imaging of a patient with GBC with primary suspicion of cholecystitis on preoperative investigation. (B) Imaging of a patient with cholecystitis with primary suspicion of malignant disease on preoperative investigation.

pathology. Several studies have evaluated GBC protein biomarkers at different tumor stages,[9,11] including extracellular vesicles[12] and plasma proteins. In 2021, a study identified 29 proteins from plasma-derived extracellular vesicles that were uniquely dysregulated in early stage GBC and clinically verified three proteins (NT5E, ANPEP, and MME).[12] Tissue[13,14] and plasma-derived extracellular vesicles[12] have been identified as potential biomarkers for GBC stages. It is noteworthy that, to our knowledge, no GBC biomarker discovery study has screened more than 2,600 proteins.[10] These previous studies relied on statistical tests to compare GBC and control groups. The use of such frequency-based analyses can lead to high false-positive rates owing to multiple testing.[16] They only show associations between the disease and the biomarker, and cannot necessarily be translated to predictive potential.[17] Bayesian model-based machine learning (ML) methods can improve biomarker discovery in large datasets as follows: (1) they are fitted to high-dimensional data, particularly when the number of variables outweighs observations and (2) they optimize biomarker selection based on outcome predictability (*i.e.* GBC *vs.* cholecystitis).[18–20]

In this study, we aimed to investigate whether preoperative assessment of plasma proteins in samples can distinguish GBC from cholecystitis and to explore whether proteomics could be used in the future to predict cancer preoperatively.

Materials and methods

This retrospective single-center study was approved by the Swedish Ethical Review Authority (Dnr 2013/188-31, 2014/2118-32, 2020-02702) and was performed in accordance with the Strengthening the Reporting of Observational Studies in Epidemiology guidelines.[21]

Study population and design

Adults (≥18 years of age) who underwent bisegmentectomy or wedge resection (anatomical resection of liver segments 4b and 5 or part of these segments) with lymphadenectomy and frozen sections of the cystic duct for suspected GBC at Karolinska University Hospital (2009–2020) were screened for inclusion in the study.

Based on the pathology report after resection, patients were divided into two groups: inflammation (cholecystitis) and GBC. Individuals with advanced tumors requiring more extensive resection, immunosuppressive treatment, surgery for incidental GBC from a previous cholecystectomy, or diagnoses other than GBC or cholecystitis were excluded. After screening, two groups of similar sizes were obtained.

Clinical data of patients from the cholecystitis (n = 38) and GBC (n = 44) groups and their correlation with proteomics data were explored using a computational technique called singular value decomposition (SVD) analysis. In SVD, principal component analysis (PCA) was first performed to reduce data dimensionality, and then the correlation of clinical parameters with the principal components was statistically tested (see Fig. S1A).

Surgical management

During the study period, all patients with suspected GBC were discussed at a multidisciplinary team conference where the decision to recommend surgery and the operating strategy were taken. Individuals with a preoperative suspicion of GBC were operated (2009–2020) and divided into two groups, cholecystectomy and GBC, based on the histology of the operative specimen. All patients had a frozen section from the cystic duct, and if positive for cancer or high-grade dysplasia, resection of the extrahepatic bile duct was performed with hepaticojejunostomy. All patients had at least one regional lymphadenectomy extending to stations 8a, 12a, b, c, and p.

Proteomics screening

All patients who underwent surgery at Karolinska University Hospital since 2019 were asked to provide written consent for inclusion in the prospective biobank consisting of all hepatobiliary surgeries. Preoperative EDTA plasma samples were

collected on the day of surgery, centrifuged, aliquoted, frozen, and stored at -80 °C. Plasma samples acquired from patients preoperatively were analyzed using an aptamer-based proteomics platform, SomaScan®[22] (SomaLogic, Boulder, CO, USA), using a 7 K assay. This novel technology ensures specific binding to different proteins and enables their accurate quantification. All raw data are publicly available at https://doi.org/10.6084/m9.figshare.26388166. Additional details are provided in the Supplementary CTAT Table.

Statistical and computational analyses

A total of 80 patients was sufficient to reach an acceptable power of 80% (medium effect size and a significance level of 0.05), with a significance level of 0.05. All statistical analyses were performed using R version 4.3.1 (2023-06-16, R Foundation for Statistical Computing, Vienna, Austria). The data, in the '.adat' format were loaded and manipulated using the SomaDataIO package.[7] The quality of the data was controlled and then centered and scaled for downstream analysis. Proteins from organisms other than humans and duplicate isotopes were excluded from analysis.

ML methods

The elastic net (EN) regularization method, based on generalized linear models, was used as a feature (variable) selection method using the glmnet v4.1.8 package in R and the caret package v6.0-94. Data were split into training and test sets (80% to 20%) with respect to the GBC and cholecystitis groups, and we used the leave-one-out cross-validation technique. The models were run iteratively with different hyperparameters (alpha and lambda) to determine the lambda parameter with the highest model performance, best fit, and lowest complexity for a given alpha. Setting alpha defines the regularization method that is used. The least absolute shrinkage and selection operator (LASSO) regression (alpha = 1) defines a stringent cut-off of less informative variables, whereas a strictly positive alpha that is <1 implements an EN regression where collinear variables are maintained in the model (Fig. 2). Model performance was evaluated using the root mean square deviation (RMSE), mean square deviation (MSD), correlation (R^2), receiver-operating characteristic curve (ROC), and area under the ROC curve (AUC) metrics.

Association analysis and clustering

We calculated the standardized mean differences and the 95% CIs for each protein using an independent *t* test to compare the mean levels between the cholecystitis and GBC groups. Dimensionality reduction (PCA) was used to visualize the data. The clustering potential of plasma proteins identified by ML was tested using unsupervised clustering methods (k-means clustering and hierarchical clustering).

Enrichment analysis

Using all the proteins found by the EN methods and the statistical significance test, we performed enrichment analysis, using Kyoto Encyclopedia of Genes and Genomes (KEGG) pathway (https://www.genome.jp/kegg/pathway.html) and Gene Ontology (GO, https://geneontology.org/) databases, clusterProfiler v4.8.3 R package, Enrichr,[23] and ShinyGO web-tool.[24] All scripts used in computational analysis are available at GitHub (https://github.com/MedH-AB-group/Cancer_plasma_proteomics).

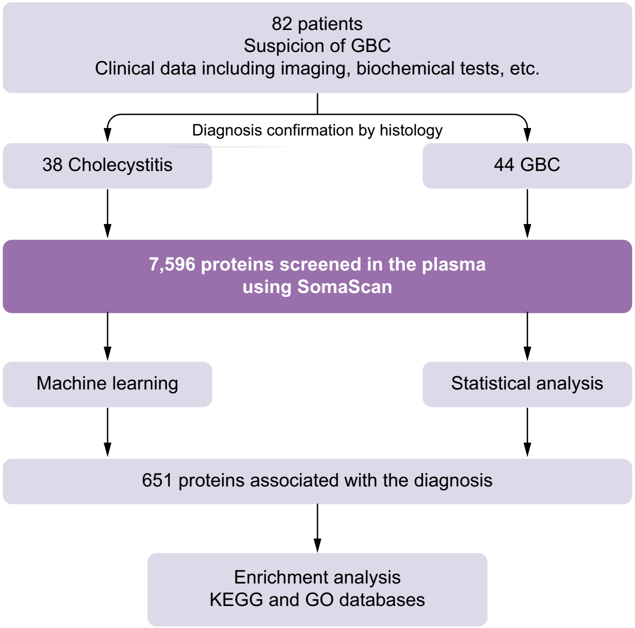

Fig. 2. Flow chart of the study design and methods used. GBC, gallbladder cancer; GO, Gene Ontology; KEGG, Kyoto Encyclopedia of Genes and Genomes.

Results

Overall, 82 patients with clinical and radiological suspicion of GBC were included in this study. Of these, 44 patients had GBC in the pathology report and 38 had cholecystitis. The clinicopathological characteristics are shown in Table 1.

Before surgery, radiological investigation with either computed tomography (CT) scan (57%), magnetic resonance imaging (MRI) (10%), or both (32%) was performed. The use of preoperative radiology was similar in patients with confirmed GBC and those with a postoperative cholecystitis diagnosis.

We assessed the relationships between clinical factors and proteomics profile using SVD analysis (Fig. S1A). Liver cirrhosis and primary sclerosing cholangitis did not show any significant associations, whereas diabetes displayed correlation (significant at p <0.05) with ~5% of the proteomic data (Table 1 and Fig. S1A).

Large-scale plasma proteomic signature distinguishes cholecystitis from GBC

The primary objective was to determine whether circulating proteins detected preoperatively in the peripheral blood can accurately distinguishing between patient groups (malignancy or cholecystitis). We identified 267 proteins in the plasma that were differentially expressed between the groups (Fig. 3A), using statistical tests. Notably, three of these proteins exhibited the highest standard mean difference (SMD >0.73) in this study, indicating prominent group differences: the cysteine-rich secretory protein 2 (CRIS2) with a raw mean difference (MD) of 381 (95% CI 142–619), the homeobox protein HMX2 (MD 70.7, 95% CI 26–115), and the sarcoplasmic endoplasmic

Table 1. General description of clinical characteristics of the cohort.

	Cholecystitis n = 38	GBC n = 44	p value (X^2)
**General description**			
Age, median (IQR)	63.5 (18.2)	69 (12.5)	0.036
Sex, n (%)			
Female	16 (42)	26 (59)	
Male	22 (58)	18 (41)	
BMI, median (IQR)	27 (8)	25.9 (5.7)	0.241
Diabetes, n (%)	7 (18)	4 (9)	
Immunosuppressive medication, n (%)	0	0	
Primary sclerosing cholangitis (PSC), n (%)	0	7 (16)	
Cirrhosis, n (%)	0	1 (2)	
**Baseline tests**			
CRP, median (IQR)	2 (4.5)	2 (5.8)	0.227
Albumin, median (IQR)	36 (4)	33 (6)	0.009
Bilirubin, median (IQR)	6 (4)	6 (6)	0.616
CA199, median (IQR)	15 (12)	110 (110.5)	0.076
**Imaging**			
CT, n (%)	34 (89)	39 (88)	
MRI, n (%)	17 (45)	17 (39)	
Both CT and MRI, n (%)	13 (34)	13 (29)	
Preoperative biliary stenting, n (%)	1 (2)	4 (9)	
**Pathological report**			
Inflammation	All	–	
T stage, n (%)	–		
In situ		1 (3)	
1		3 (9)	
2		17 (53)	
3		11 (34)	
4		0	
Lymph node status, n (%)	–		
N0 (0 positive nodes)		19 (43)	
N1 (1–3 positive nodes)		11 (25)	
N2 (≥4 positive nodes)		2 (4)	
NA		12 (27)	
M status, n (%)	–		
M0		42 (95)	
M1 (aortocaval nodes)		2 (5)	

p values are calculated based on the X^2 test. CRP, C-reactive protein; GBC, gallbladder cancer; n, number of occurrences; n, number of patients.

reticulum calcium ATPase 3 (AT2A3) (MD 143, 95% CI 51–235). Given the large number of proteins tested (7,500) relative to the modest sample size (n = 82), we recognize the importance of addressing the multiple testing issue to avoid false positives.

Given the high dimensionality of the data, we opted for the regularization method EN, an ML method that (1) can handle high-dimensional datasets, (2) prevents overfitting by utilizing Bayesian statistics, and (3) balances between retaining correlated variables and performing variable selection.[25] Unlike traditional methods, which may exhibit multicollinearity, EN can identify proteins that contribute jointly to the model even if they are highly correlated. To explore the multivariate association within the proteomic data, we applied an EN-regularized regression. This ML model incorporated a subset of 577 proteins associated with postoperative diagnosis. The EN model achieved a high performance on the test set, with an AUC of 94%. The model's mean squared error and the root mean squared error were low, at 0.01 and 0.12, respectively, while the R^2 metric was 93%, indicating a strong fit to the data. Collectively, these measurements underscored the capacity of this subset of proteins to differentiate between the GBC and cholecystitis profiles, supporting the robustness of our multivariate approach.

Notably, 193 proteins (29.6%) identified through EN also appeared in the statistical t test results (Fig. 3B), exhibiting a medium to large effect SMD ranging between 0.4 and 0.75. This overlap underscores the significance of these proteins, even when considering the limitations of a small sample size and the burden of multiple testing.

Combining the results from the frequentist statistical analysis and the ML results, we identified diagnosis-associated proteins (651) affected by both upregulation (316 proteins) and downregulation (341 proteins). In the PCA performed using the whole proteomic dataset (7,500 proteins), no data pattern was observed (Fig. 3C). However, using the proteins identified by the EN and t test, amounting to 651 proteins, revealed a strong differentiation of GBC and cholecystitis (Fig. 3D and E).

ML methods identify a minimal plasma protein signature for preoperative diagnosis of GBC

By adjusting the alpha parameter in our ML model and performing LASSO regression, we identified a more parsimonious set of 13 proteins (Fig. 4) associated with the postoperative diagnosis. This refined model achieved an AUC of 98% in the test set, suggesting a strong discriminatory capacity for these proteins within the cohort. Although not aimed at predictive applications, the high AUC underscores the potential of these selected proteins to capture key multivariate relationships distinguishing GBC from cholecystitis profiles. Interestingly, 12

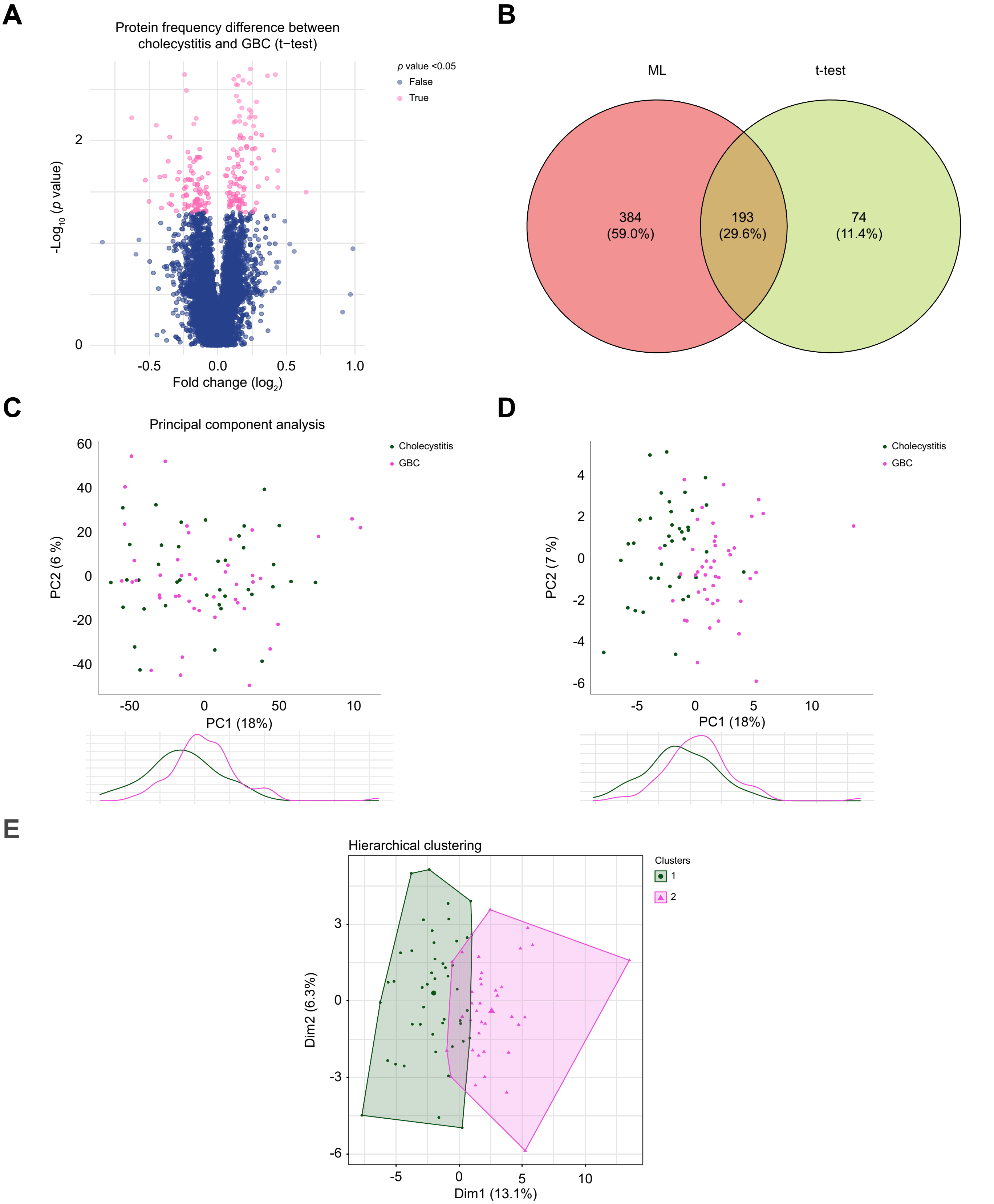

Fig. 3. A set of 651 proteins were associated with diagnosis. (A). Volcano plot of all the proteins (in blue) and those significantly (p <0.05) linked to the diagnosis (in pink), in t test. (B) Venn diagram of the overlap between results from elastic net (n = 577), and t test at p <0.05 (n = 267). (C) Principal component analysis (PCA) of the whole dataset (7,500 proteins) with no apparent data structure. (D) PCA using the 651 diagnosis-associated proteins showed separation of GBC and cholecystitis. (E) Unsupervised hierarchical clustering of diagnosis-associated proteins largely distinguished GBC and cholecystitis, yet in the central region these groups overlapped (11% of the patients). Dim, dimension; GBC, gallbladder cancer; PC, principal component.

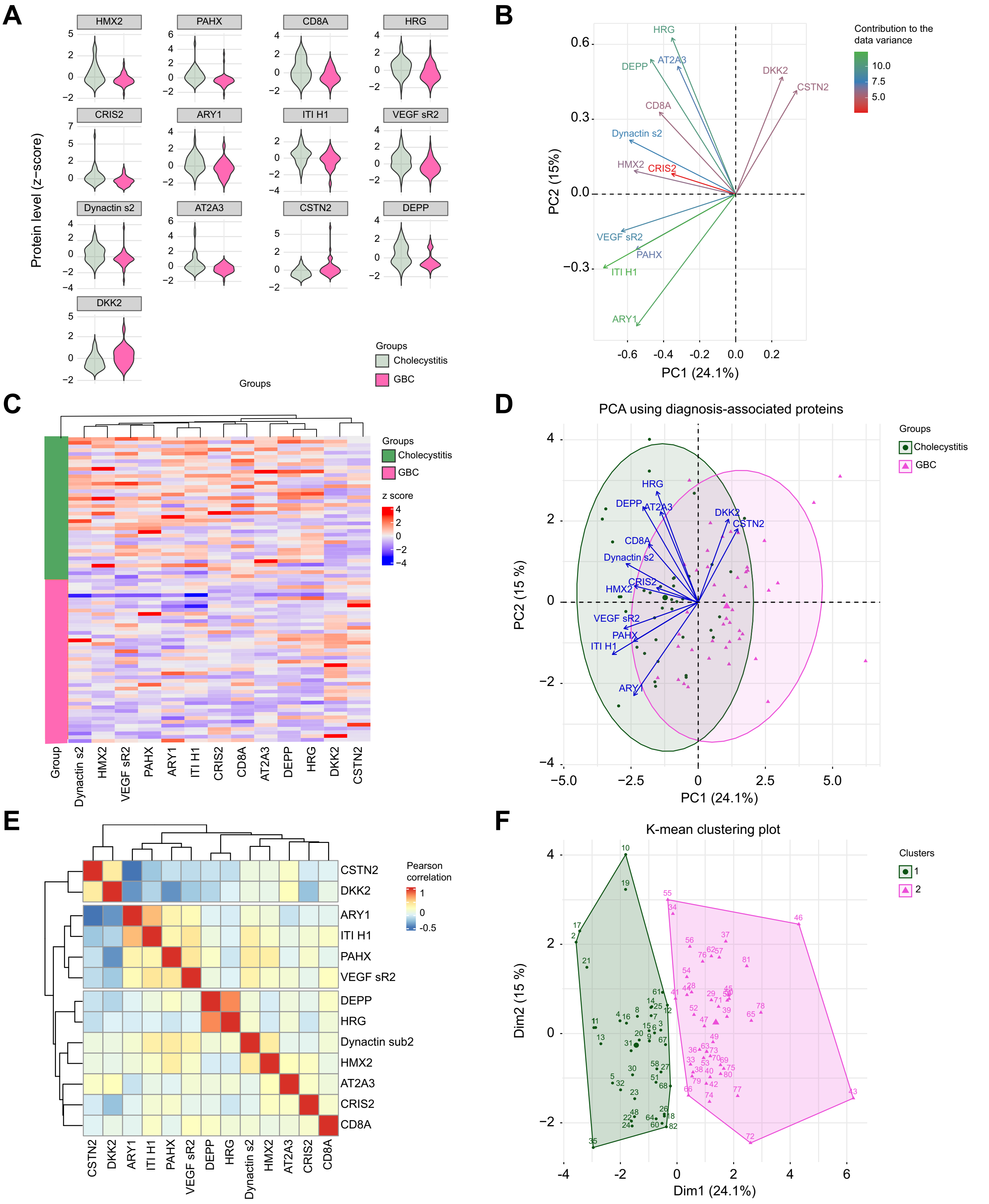

Fig. 4. Characteristics of the most descriptive proteins of postoperative diagnosis. (A) The variance of the protein levels in patients with cholecystitis and GBC. (B) Direction and contribution of each protein to the data variance, assessed by principal component analysis (PCA). (C) Heatmap showing the difference of expression of the proteins between the two groups. (D) Data variance and group differences are well described by the proteins, visualized by PCA. (E) Low correlation between the proteins confirming non-redundant information in the ML model. (F) Thirteen proteins could separate the patients into two clusters (k-mean unsupervised clustering) that largely correspond to the cholecystitis and GBC groups (89% of the patients were rightfully assigned). All nine wrongfully assigned patients were in the middle area. PCA, principal component analysis.

of these proteins had the highest SMD in this study, ranging from 0.64 to 0.74. The exception was dickkopf-related protein 2 (DKK2), which had a medium SMD of 0.3. This streamlined protein set further reinforces the robustness of our multivariate approach and offers insight into potentially influential biomarkers.

Next, we further explored the proteins selected by the ML model in relation to the dataset. Individually, no protein showed a distinct frequency difference between GBC and cholecystitis (Fig. 4A and C). However, PCA demonstrated that the proteins largely accounted for data variability and spanned their directions (Fig. 4B and D). K-means clustering, using the 13 same proteins, resulted in an unsupervised bifurcation of the patients into two clusters (*i.e.* groups), largely aligning with the cholecystitis and GBC groups (Fig. 4F). Eleven percent of the patients were misclassified and all fell within the intermediate area. K-means clustering optimizes grouping by minimizing intragroup distances and maximizing intergroup distances. However, for data points in the intermediate area, patients were not accurately assigned to their respective groups. In fact, upon iteration with different cluster quantities (changing the K value), the optimal cluster count was three, where patients in the middle constituted a distinct group.

Pathway enrichment analysis reveals biological mechanisms underlining cholecystitis and GBC

To gain insight into the molecular functions and biological pathways overrepresented in these 651 diagnosis-associated proteins, we performed an enrichment analysis. Cytokine receptor binding as well as activity proteins and the pro-inflammatory cytokine pathway (TNF signaling pathway) were significantly (adjusted $p < 0.01$) enriched both in the proteins' molecular functions and the biological pathways in which they were involved (Fig. 5A and B), as expected in the presence of inflammation. The set was also enriched (adjusted $p < 0.01$) in proteins related to growth development (serine activity and growth factor binding), membrane receptors (transmembrane receptors and G protein binding), and glycosaminoglycan-binding functions. Based on KEGG brites (Fig. 5C), several diagnosis-associated proteins belonged to membrane trafficking, chromosome-associated proteins, and transcription factors. Notably, four transcription factor families were significantly enriched in diagnosis-associated proteins ($p < 0.01$) (Fig. 5D and Table S2). Finally, we connected HGF, CDK2, FADD, and other proteins to the cancer pathways in the KEGG database (Fig. 5E).

Discussion

In this retrospective exploratory study, we investigated the potential of the plasma proteome to preoperatively differentiate between GBC and cholecystitis. We aimed to identify a minimum set of proteins that could reflect the postoperative diagnosis before surgery. Plasma samples from 82 individuals who underwent liver resection were analyzed. Using ML techniques, protein subsets could differentiate patients with a post-operative histological diagnosis of GBC from patients with cholecystitis with 98% accuracy. Indeed, reliable clustering is not feasible for a minor subset of patients. This study, although exploratory and lacking external validation, used a reasonable patient pool, screened a large number of proteins (7,500 proteins), and used cutting-edge and suitable computational techniques. Our findings suggest that the preoperative measurement of plasma proteins could be informative regarding the presence of GBC. Preoperative blood tests could potentially be developed to enhance diagnostic accuracy, particularly in individuals with pre-existing inflammatory conditions.

The SomaScan® technology from SomaLogic is a pioneering high-throughput proteomics technology that allows data-driven biomarker discovery. Modified aptamers are called SOMAmers and are small DNA sequences with high-affinity target-specific tertiary structures. Their slow off-rate binding kinetics were measured using microarray-based fluorescence intensity readout.[22] This affinity-based approach overcomes the challenge of detecting proteins at vastly different concentrations, known as the dynamic range issue, and outperforms mass spectrometry.[26] Our choice of ML regularization method was motivated by the high dimensionality of the data. EN and LASSO are robust variable (proteins in this case) selection methods that integrate ML techniques, such as cross-validation and training, to optimize the model for outcome predictability (diagnosis in this case). In addition, approximately one-third of the proteins identified by EN overlapped with the statistical t test results, reinforcing the relevance of these proteins despite the challenges posed by the limited sample size and multiple testing. This overlap suggests that although individual statistical significance may not have survived strict multiple testing correction, the proteins identified by both methods likely reflect biologically meaningful associations with the disease. Together, these analyses highlight the importance of considering complementary approaches, such as EN in large proteomic studies where traditional statistical methods may be overly conservative.

Among the identified diagnosis-associated proteins, several hold promise for clinical application. For example, CD8 chain (T-cell surface glycoprotein CD8 alpha chain [CD8A]), HMX2, histidine-rich glycoprotein (HRG), ATPase Sarcoplasmic/Endoplasmic Reticulum Ca2+ Transporting 3 (AT2A3), and the decidual protein induced by progesterone (DEPP)[27] can be tested using commercially available ELISA or Western blot kits. In addition, some of these proteins are associated with oncogenesis and progression of GBC. Arylamine N-acetyltransferase 1 (ARY1) is a key enzyme in various cancers, including GBC.[28,29] It is upregulated in several cancer types and can be a target for cancer therapy and patient stratification.[30] Vascular endothelial growth factor receptor 2 (VEGFR2) is a well-known target in cancer therapy because of its role in tumor induced angiogenesis.[31,32] Histidine-rich glycoprotein (HRG) has been detected in plasma and has been suggested as a potential prognostic and diagnostic biomarker in cancer.[33,34] Finally, CD8A, a component of the CD8 co-receptor complex, has been linked to GBC subtypes and prognosis.[35] In contrast, diagnosis-associated proteins include several transcription factors, including HNF4A and PRDM1, which were previously associated with cholangiocarcinoma.[36,37] The NFAT2 family, the most significantly enriched ($p = 0.0001$) family in our results, has been linked to the progression of bladder urothelial carcinoma[38] and various human solid tumors and hematologic malignancies.[39] Our results highlight the potential relevance of these proteins to GBC pathobiology.

However, knowledge regarding the etiology of GBC is limited. A history of gallstones and cholecystitis is the strongest

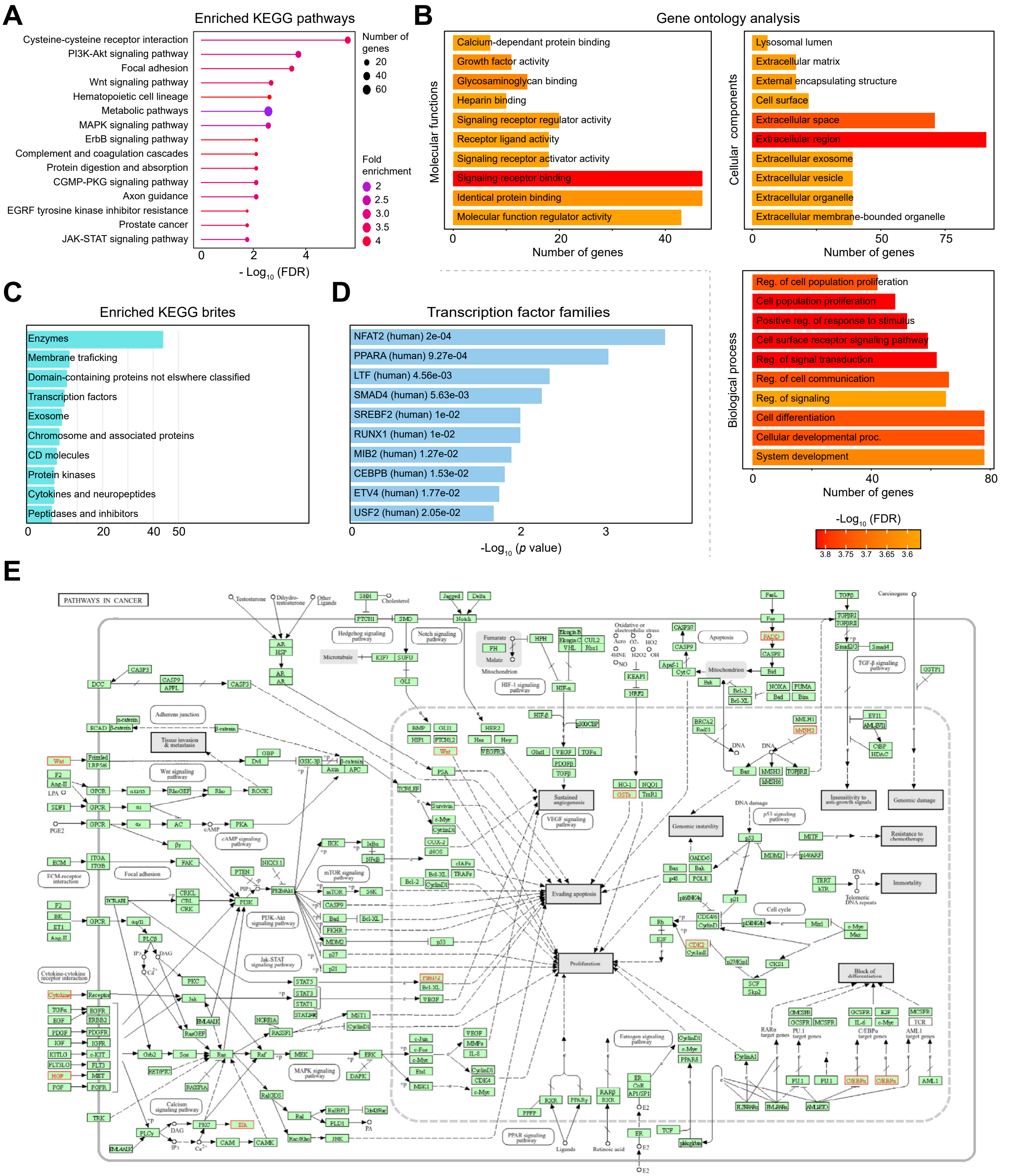

Fig. 5. Enrichment analysis of the proteins linked to diagnosis. (A) Enriched KEGG pathways in the diagnosis-associated proteins (651) where cytokine receptors are the most significant. (B) Gene Ontology analysis of the molecular functions, the cellular components, and the biological processes of the diagnosis-associated proteins. (C) Enriched KEGG brites in diagnosis-associated proteins. (D) Transcription factor families enriched in the protein list. (E) Cancer pathways in KEGG database with dysregulated proteins (from this study) highlighted in red. FDR, false discovery rate; KEGG, Kyoto Encyclopedia of Genes and Genomes. N. of genes, number of genes. *p* values are calculated based on Fisher's exact test.

known risk factor.[40] Other factors include advanced age, female sex,[41] chronic bacterial infections (*Salmonella* and *Helicobacter*),[40] and primary sclerosing cholangitis.[42] The etiology and related biochemical alterations in blood during GBC development remain largely unknown. Here, we describe a set of proteins that may shed light on the pathogenesis of GBC. Several proteins identified in our analysis are implicated in the pathophysiology of GBC and other biliary tract cancers, suggesting few pathways that are critical to tumor progression and immune evasion. For example, phytanoyl-CoA dioxygenase (PAHX) is involved in peroxisomal metabolism,[43] and its alterations may disrupt lipid metabolic pathways, which have been increasingly linked to tumorigenesis in biliary tract cancers.[44] The DEPP protein, known for its role in oxidative stress responses,[45] may contribute to the heightened oxidative environment that facilitates cancer progression.[46] CRISP2 is a member of the cysteine-rich secretory protein family and has been linked to immunomodulation and pathophysiology of chronic pancreatitis.[47] This protein was likely linked to the diagnosis of cholecystitis in our study. VEGF sR2, a soluble form of VEGF receptor, is crucial for vascular endothelial growth in biliary pathophysiology and angiogenesis regulation. Its dysregulation is a known factor in biliary tract cancers, where angiogenesis supports tumor growth and metastasis.[48,49] HRG and DKK2 are involved in immune modulation and Wnt signaling,[50] respectively. Both pathways play essential roles in cancer progression and anticancer immune response.[51,52] Additionally, CD8A, a protective gene with the highest correlation with T cells, is a promising prognostic biomarker in bladder cancer,[53] calsyntenin-2 (CSTN2) has been identified as a potential biomarker for colorectal cancer,[54] and dynactin subunit 2 has been identified as an oncogene in hepatocellular carcinoma.[55]

Notably, we found a significant association between serpin family A (SERPINA1), Annexin A protein 3 (ANXA3), collagen type VI alpha 1 chain (COL6A1), proteinase 3 (PRTN3), and keratin (KRT18) with GBC diagnosis, through statistical tests and ML techniques. These proteins have been previously associated with early stage GBC.[13,56] Additionally, KRT1, ANXA2, peptidyl-prolyl cis-trans isomerase B (PPIB), colony stimulating factor 1 (CSF1), and receptor tyrosine-protein kinase (ERBB3) were identified using ML methods in this study. The first three proteins were recently detected in tissue samples and were associated with lymph node metastasis in GBC,[14] whereas ERBB3 has been described as a targeted therapy for GBC.[59] Recently, CSF1 was described as a potential preoperative plasma marker for biliary tract cancer.[60]

Collectively, these proteins provide insights into the multifaceted biological mechanisms driving the development of GBC and other bile duct cancers and may serve as promising future plasma biomarkers for preoperative GBC diagnosis. Our findings complement those of previous studies, in which plasma biomarkers have been reported for the diagnosis and prognosis of GBC.[8] In a study by Wang *et al.*[8], a combination of plasma markers (CA125, CA242, and CA199) enhanced the diagnostic sensitivity of GBC compared with individual markers. Notably, our extensive protein panel did not include CA19-9 or CA242, and we found a low SMD for CA125 in our cohort. Furthermore, carcinoembryonic antigen (CEA) exhibited an SMD of 0.2, suggesting a limited role as a diagnostic marker in our analysis. This highlights the need for further investigation of alternative biomarkers that may provide improved diagnostic capabilities for GBC.

The molecular functions and biological pathways identified in our study are related to cancer factors and infections. Signal transduction and signaling molecules and interactions, such as cytokine receptor-related pathways and the TNF signaling pathway, were significantly enriched. These pathways are characteristic of inflammation as seen in cholecystitis.[61] The MAPK, JAK-STAT, and PI3K/AKR pathways, which were significantly dysregulated in our study, are associated with inflammation, cholecystitis, and tumor progression.[62,63] ErbB and the coagulation and complement cascade pathways were associated with thromboinflammatory process regulation in tumor progression[2] and were enriched in downregulated proteins in our analysis. The axon guidance pathway, which is altered in pancreatic ductal adenocarcinoma,[1] was enriched in this study. Lastly, the Wnt signaling pathway was significantly enriched with upregulated proteins, including DDK2, which was highly associated with GBC diagnosis in our study. This pathway promotes colon carcinogenesis.[64]

This study had several limitations. Because of the rarity of GBC, a relatively small number of samples were studied. However, this was considered when choosing analysis methods that were adapted to such conditions. Nevertheless, the results are exploratory and further validation of the present results is required. A validation step is feasible and cost-effective because of the low number of proteins, their availability for clinical testing, and their detection at the plasma level. Importantly, validation efforts would require a larger collection of samples and, potentially, multicenter and international collaboration.

In conclusion, to our knowledge, our study provides the largest proteomic characterization of GBC that can serve as a resource for further biomarker discovery. We show that preoperative assessment of plasma proteins can accurately differentiate cholecystitis from malignancy, supporting the development of a noninvasive test to assist preoperative decision-making when GBC is suspected.

Affiliations

[1]*Division of Hepatology, Department of Medicine, Huddinge, Karolinska Institutet, Stockholm, Sweden;* [2]*Center for Infectious Medicine, Department of Medicine Huddinge, Karolinska Institutet, Stockholm, Sweden;* [3]*Division of Surgery and Oncology, Department of Clinical Science, Intervention and Technology, Karolinska Institutet, Stockholm, Sweden;* [4]*Department of Upper GI Disease Karolinska University Hospital, Stockholm, Sweden*

Abbreviations

ANXA, annexin A; ARY1, arylamine N-acetyltransferase 1; AT2A3, sarcoplasmic/endoplasmic reticulum calcium ATPase 3; AUC, area under the ROC curve; CD8A, T-cell surface glycoprotein CD8 alpha chain; CEA, carcinoembryonic antigen; COL6A1, collagen type VI alpha 1 chain; CRIS2, cysteine-rich secretory protein 2; CSF1, colony stimulating factor 1; CSTN2, calsyntenin-2; CT, computed tomography; DEPP, decidual protein induced by progesterone; DKK2, dickkopf-related protein 2; dynactin sub2, dynactin subunit 2; EGRF, epidermal growth factor receptor; EN, elastic net; ERBrbB3, receptor tyrosine-protein kinase erbB-3; FDR, false discovery rate; GBC, gallbladder cancer; GO, Gene Ontology; HMX2, homeobox protein HMX2; HRG, histidine-rich glycoprotein; Is, *in situ*; ITI H1, inter-alpha-trypsin inhibitor heavy chain H1; KEGG, Kyoto Encyclopedia of

Genes and Genomes; KRT, keratin protein; LASSO, least absolute shrinkage and selection operator; MD, mean difference; ML, machine learning; MRI, magnetic resonance imaging; MSD, mean square deviation; PAHX, phytanoyl-CoA dioxygenase peroxisomal; PC, principal component; PCA, principal component analysis; PPIB, peptidyl-prolyl cis-trans isomerase B; PRTN, proteinase; RMSE, root mean square deviation; ROC, receiver operating characteristic curve; SERPINA, serpin family A; SMD, standard mean difference; SVD, singular value decomposition; TNF, tumor necrosis factor; VEGF sR2, soluble vascular endothelial growth factor receptor 2.

Financial support

This study was supported by grants from the Swedish Society for Medical Research (SSMF), the Center for Innovative Medicine at Karolinska Institutet and RegionStockholm. The funding sources were not involved in the design or conduct of the research, nor the analysis and interpretation of the data, or the writing of this manuscript and decision to submit the article for publication to this journal.

Conflicts of interest

The authors declare no conflicts of interest that pertain to this work.

Please refer to the accompanying ICMJE disclosure forms for further details.

Authors' contributions

Contributed to the study concept and design: MC, AB, ES. Contributed to data acquisition: MC, AA, HJ. Performed the bioinformatics and computational analyses: GN. Interpreted data and drafted the manuscript: GN, MC, AB, ES. Critically revised and approved the final version of the manuscript: all authors.

Data availability statement

Raw data is available on https://doi.org/10.6084/m9.figshare.26388166. Public access to the data is restricted by the Swedish laws and regulations that prohibit the release of individual-level datasets that could potentially allow a personal identification. In this study, this particularly concerns some of the clinical metadata and patients' s characteristics. However, data access can be granted in the framework of a defined academic research collaboration with requirement of data transfers agreement. Anyone wishing to gain access to the data should contact Martin Cornillet and Ernesto Sparrelid (martin.cornillet.jeannin@ki.se, ernesto.sparrelid@ki.se).

Acknowledgements

Preliminary data were presented at the ESMO GI 2024 congress.

Supplementary data

Supplementary data to this article can be found online at https://doi.org/10.1016/j.jhepr.2025.101365.

References

Author names in bold designate shared co-first authorship

[1] Deng YL, Cheng NS, Zhang SJ, et al. Xanthogranulomatous cholecystitis mimicking gallbladder carcinoma: an analysis of 42 cases. World J Gastroenterol 2015;21:12653–12659.
[2] Kim SH, Jung D, Ahn JH, et al. Differentiation between gallbladder cancer with acute cholecystitis: considerations for surgeons during emergency cholecystectomy, a cohort study. Int J Surg 2017;45:1–7.
[3] Yu MH, Kim YJ, Park HS, et al. Benign gallbladder diseases: imaging techniques and tips for differentiating with malignant gallbladder diseases. World J Gastroenterol 2020;26:2967–2986.
[4] Matsui S, Tanioka T, Nakajima K, et al. Surgical and oncological outcomes of wedge resection versus segment 4b + 5 resection for T2 and T3 gallbladder cancer: a meta-analysis. J Gastrointest Surg 2023;27:1954–1962.
[5] Dincer HA, Cennet O, Dogrul AB. The utility of systemic immune inflammatory index in discriminating between gallbladder cancer and xanthogranulomatous cholecystitis: a single-tertiary center experience. Medicine (Baltimore) 2023;102:e35805.
[6] **Zhang J**, **Wu Y**, **Feng Y**, et al. The value of CT findings combined with inflammatory indicators for preoperative differentiation of benign and malignant gallbladder polypoid lesions. World J Surg Oncol 2023;21:51.
[7] Zhou QM, Liu CX, Zhou JP, et al. Machine learning-based radiological features and diagnostic predictive model of xanthogranulomatous cholecystitis. Front Oncol 2022;12:792077.
[8] Wang YF, Feng FL, Zhao XH, et al. Combined detection tumor markers for diagnosis and prognosis of gallbladder cancer. World J Gastroenterol 2014;20:4085–4092.
[9] Baichan P, Naicker P, Augustine TN, et al. Proteomic analysis identifies dysregulated proteins and associated molecular pathways in a cohort of gallbladder cancer patients of African ancestry. Clin Proteomics 2023;20:8.
[10] **Sahasrabuddhe NA**, **Barbhuiya MA**, Bhunia S, et al. Identification of prosaposin and transgelin as potential biomarkers for gallbladder cancer using quantitative proteomics. Biochem Biophys Res Commun 2014;446:863–869.
[11] Tan Y, Ma SY, Wang FQ, et al. Proteomic-based analysis for identification of potential serum biomarkers in gallbladder cancer. Oncol Rep 2011;26:853–859.
[12] Priya R, Jain V, Akhtar J, et al. Plasma-derived candidate biomarkers for detection of gallbladder carcinoma. Sci Rep 2021;11:23554.
[13] Akhtar J, Jain V, Kansal R, et al. Quantitative tissue proteome profile reveals neutrophil degranulation and remodeling of extracellular matrix proteins in early stage gallbladder cancer. Front Oncol 2023;12:1046974.
[14] Jain V, Akhtar J, Priya R, et al. Tissue proteome analysis for profiling proteins associated with lymph node metastasis in gallbladder cancer. BMC Cancer 2023;23:402.
[16] Altman N, Krzywinski M. The curse(s) of dimensionality. Nat Methods 2018;15:399–400.
[17] Mann M, Kumar C, Zeng WF, et al. Artificial intelligence for proteomics and biomarker discovery. Cell Syst 2021;12:759–770.
[18] Vashistha R, Noor Z, Dasgupta S, et al. Application of statistical machine learning in biomarker selection. Sci Rep 2023;13:18331.
[19] Hernández B, Pennington SR, Parnell AC. Bayesian methods for proteomic biomarker development. EuPA Open Proteomics 2015;9:54–64.
[20] Crook OM, Chung C, Deane CM. Challenges and opportunities for Bayesian statistics in proteomics. J Proteome Res 2022;21:849–864.
[21] von Elm E, Altman DG, Egger M, et al. The Strengthening the Reporting of Observational Studies in Epidemiology (STROBE) statement: guidelines for reporting observational studies. Bull World Health Organ 2007;85:867–872.
[22] Tin A, Yu B, Ma J, et al. Reproducibility and variability of protein analytes measured using a multiplexed modified aptamer assay. J Appl Lab Med 2019;4:30–39.
[23] Gene Set Knowledge Discovery with Enrichr - Xie - 2021 - Current Protocols - Wiley Online Library. https://currentprotocols.onlinelibrary.wiley.com/doi/10.1002/cpz1.90 (Accessed July 10, 2024).
[24] Ge SX, Jung D, Yao R. ShinyGO: a graphical gene-set enrichment tool for animals and plants. Bioinformatics 2020;36:2628–2629.
[25] Zhu W, Lévy-Leduc C, Ternès N. A variable selection approach for highly correlated predictors in high-dimensional genomic data. Bioinformatics 2021;37:2238–2244.
[26] Gold L, Ayers D, Bertino J, et al. Aptamer-based multiplexed proteomic technology for biomarker discovery. PLoS One 2010;5:e15004.
[27] ELISA Kit Search | Biocompare. https://www.biocompare.com/pfu/110627/soids/2-74765/ELISA_Kit (Accessed July 4, 2024).
[28] **Liu R**, **Wu Z**, Zhang Y, et al. Prognostic and clinicopathological significance of X-box-binding protein 1 and N-acetyltransferase 1 in gallbladder cancer. Front Oncol 2020;10:1124.
[29] Butcher NJ, Minchin RF. Arylamine N-acetyltransferase 1: a novel drug target in cancer development. Pharmacol Rev 2012;64:147–165.
[30] Leggett CS, Doll MA, Salazar-González RA, et al. Identification and characterization of potent, selective, and efficacious inhibitors of human arylamine N-acetyltransferase 1. Arch Toxicol 2022;96:511–524.
[31] Liu Y, Li Y, Wang Y, et al. Recent progress on vascular endothelial growth factor receptor inhibitors with dual targeting capabilities for tumor therapy. J Hematol Oncol 2022;15:89.
[32] Chen Y, Jiang L, She F, et al. Vascular endothelial growth factor-C promotes the growth and invasion of gallbladder cancer via an autocrine mechanism. Mol Cell Biochem 2010;345:77–89.
[33] Johnson LDS, Goubran HA, Kotb RR. Histidine rich glycoprotein and cancer: a multi-faceted relationship. Anticancer Res 2014;34:593–603.
[34] Roche FP, Pietilä I, Kaito H, et al. Leukocyte differentiation by histidine-rich glycoprotein/stanniocalcin-2 complex regulates murine glioma growth through modulation of antitumor immunity. Mol Cancer Ther 2018;17:1961–1972.
[35] Lin J, Long J, Wan X, et al. Classification of gallbladder cancer by assessment of CD8+ TIL and PD-L1 expression. BMC Cancer 2018;18:766.

[36] Song G, Shi Y, Meng L, et al. Single-cell transcriptomic analysis suggests two molecularly distinct subtypes of intrahepatic cholangiocarcinoma. Nat Commun 2022;13:1642.
[37] Wang W, Lopez McDonald MC, Hariprasad R, et al. Oncogenic STAT transcription factors as targets for cancer therapy: innovative strategies and clinical translation. Cancers 2024;16:1387.
[38] **Dai ZT**, **Xiang Y**, **Wang Y**, et al. Prognostic value of members of NFAT family for pan-cancer and a prediction model based on NFAT2 in bladder cancer. Aging (Albany NY) 2021;13:13876–13897.
[39] Mancini M, Toker A. NFAT proteins: emerging roles in cancer progression. Nat Rev Cancer 2009;9:810–820.
[40] Randi G, Franceschi S, La Vecchia C. Gallbladder cancer worldwide: geographical distribution and risk factors. Int J Cancer 2006;118:1591–1602.
[41] Rustagi T, Dasanu CA. Risk factors for gallbladder cancer and cholangiocarcinoma: similarities, differences and updates. J Gastrointest Cancer 2012;43:137–147.
[42] Said K, Glaumann H, Bergquist A. Gallbladder disease in patients with primary sclerosing cholangitis. J Hepatol 2008;48:598–605.
[43] Fan Z, Edelmann D, Yuan T, et al. Developing survival prediction models in colorectal cancer using epigenome-wide DNA methylation data from whole blood. NPJ Precis Oncol 2024;8:1–9.
[44] Kerekes DM, Khan SA. Lipid metabolism in biliary tract cancer: a new therapeutic target? Ann Surg Oncol 2022;29:2750–2751.
[45] Salcher S, Hagenbuchner J, Geiger K, et al. C10ORF10/DEPP, a transcriptional target of FOXO3, regulates ROS-sensitivity in human neuroblastoma. Mol Cancer 2014;13:224.
[46] Klaunig JE, Wang Z. Oxidative stress in carcinogenesis. Curr Opin Toxicol 2018;7:116–121.
[47] Liao Q, Kleef J, Xiao Y, et al. Preferential expression of cystein-rich secretory protein-3 (CRISP-3) in chronic pancreatitis. Histol Histopathol 2003;18:425–433.
[48] Mariotti V, Fiorotto R, Cadamuro M, et al. New insights on the role of vascular endothelial growth factor in biliary pathophysiology. JHEP Rep 2021;3:100251.
[49] Simone V, Brunetti O, Lupo L, et al. Targeting angiogenesis in biliary tract cancers: an open option. Int J Mol Sci 2017;18:418.
[50] Martin-Orozco E, Sanchez-Fernandez A, Ortiz-Parra I, et al. WNT signaling in tumors: the way to evade drugs and immunity. Front Immunol 2019;10:2854.
[51] Rolny C, Mazzone M, Tugues S, et al. HRG inhibits tumor growth and metastasis by inducing macrophage polarization and vessel normalization through downregulation of PlGF. Cancer Cell 2011;19:31–44.
[52] Zhou Y, Xu J, Luo H, et al. Wnt signaling pathway in cancer immunotherapy. Cancer Lett 2022;525:84–96.
[53] Zheng Z, Guo Y, Huang X, et al. CD8A as a prognostic and immunotherapy predictive biomarker can be evaluated by MRI radiomics features in bladder cancer. Cancers (Basel) 2022;14:4866.
[54] **Xie Q**, **Liu L**, Chen X, et al. Identification of cysteine protease inhibitor CST2 as a potential biomarker for colorectal cancer. J Cancer 2021;12:5144.
[55] Li W, Chen J, Xiong Z, et al. Dynactin 2 acts as an oncogene in hepatocellular carcinoma through promoting cell cycle progression. Liver Res 2022;6:155–166.
[56] Akhtar J, Priya R, Jain V, et al. Immunoproteomics approach revealed elevated autoantibody levels against ANXA1 in early stage gallbladder carcinoma. BMC Cancer 2020;20:1175.
[59] Jain A, Javle M. Molecular profiling of biliary tract cancer: a target rich disease. J Gastrointest Oncol 2016;7:797–803.
[60] Jansson H, Cornillet M, Sun D, et al. Preoperative immunological plasma markers TRAIL, CSF1 and TIE2 predict survival after resection for biliary tract cancer. Front Oncol 2023;13:1169537.
[61] Fu D, Hu Z, Xu X, et al. Key signal transduction pathways and crosstalk in cancer: biological and therapeutic opportunities. Transl Oncol 2022;26:101510.
[62] Zhao H, Wu L, Yan G, et al. Inflammation and tumor progression: signaling pathways and targeted intervention. Signal Transduct Target Ther 2021;6:263.
[63] Sabaawy HE, Ryan BM, Khiabanian H, et al. JAK/STAT of all trades: linking inflammation with cancer development, tumor progression and therapy resistance. Carcinogenesis 2021;42:1411–1419.
[64] Yao Y, Li X, Xu B, et al. Cholecystectomy promotes colon carcinogenesis by activating the Wnt signaling pathway by increasing the deoxycholic acid level. Cell Commun Signal 2022;20:71.

Keywords: Cholecystitis; Tumor; Biomarkers; Machine learning.
Received 31 July 2024; received in revised form 10 February 2025; accepted 13 February 2025; Available online 21 February 2025

Journal of Hepatology, Volume *7*

Supplemental information

Towards precision medicine strategies using plasma proteomic profiling for suspected gallbladder cancer: A pilot study

Ghada Nouairia, Martin Cornillet, Hannes Jansson, Annika Bergquist, and Ernesto Sparrelid

Towards precision medicine strategies using plasma proteomic profiling for suspected gallbladder cancer: A pilot study

Ghada Nouairia, Martin Cornillet, Hannes Jansson, Annika Bergquist, Ernesto Sparrelid

Table of contents

Fig. S1……………………………………………………………………………....2
Table S1…………………………………………………………………………....3
Table S2…………………………………………………………………………...21
Table S3…………………………………………………………………………...22
Supplementary references…………………………………………………….. 23

Fig. S1. Complementary data for the methods **(A1).** Singular Value Decomposition Analysis (SVD) showing the correlation of clinical parameters to the proteomic dataset (7 500 proteins). (**A2). Importance of principal components computed in the SVD analysis. (**B**)**.** Linear regression model iteration with different lambda values and the corresponding Mean-squared error (MSE). The number of proteins selected by each model is indicated at the top.

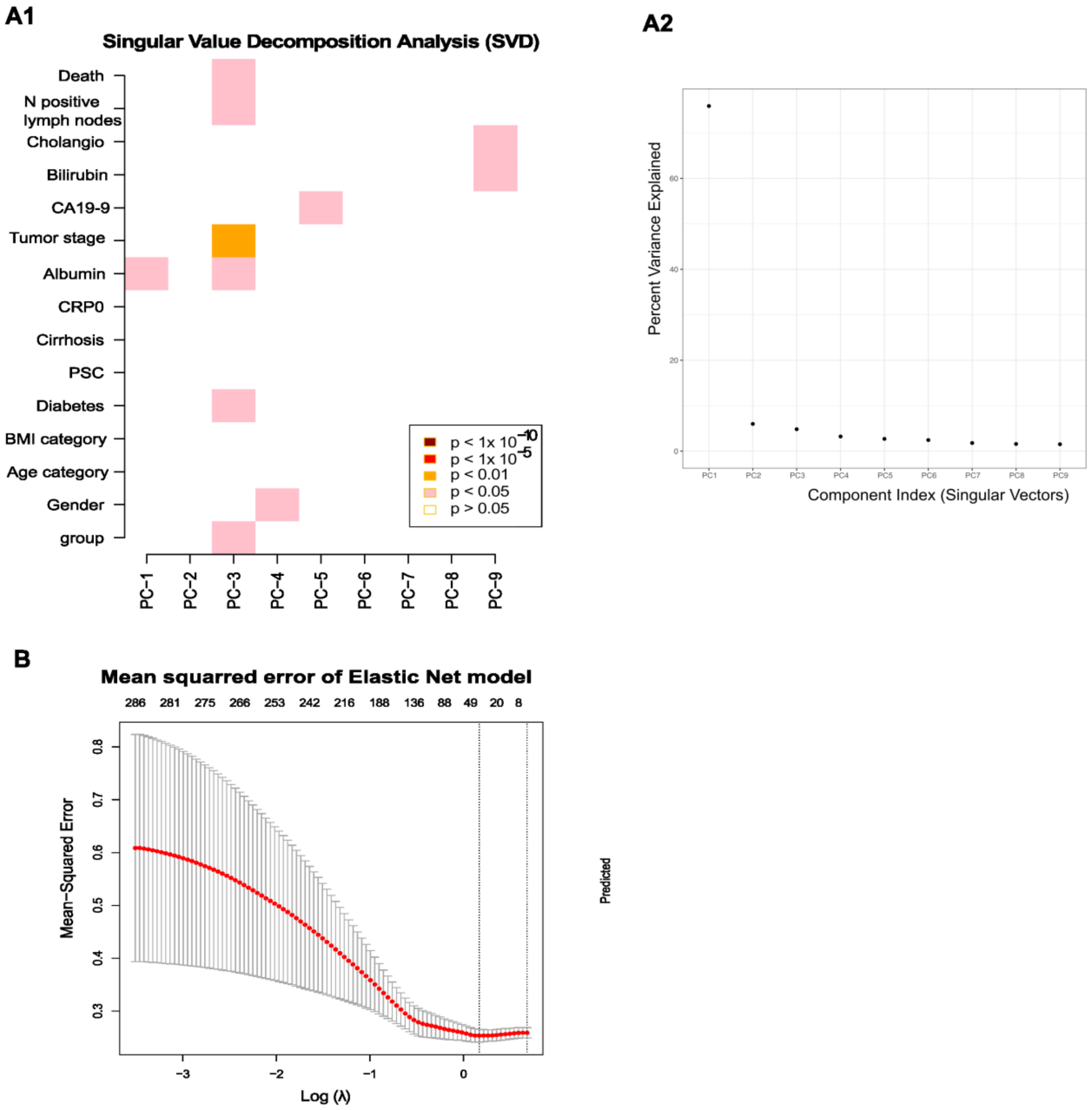

Table S1. List of the diagnosis-associated proteins (651) identified by machine learning methods (Elastic Net and LASSO) or significant statistical association based on t-test (Statistical significance cut-off for t-test is p-value < 0.05).
CI: Confidence Interval. EN: Elastic Net. GBC: Gallbladder Cancer. LASSO: Least Absolute Shrinkage and Selection Operator. MD: Mean difference. SMD: Standardized mean difference.

UniProt	Target	SMD	MD	Lower CI	Upper CI	P value	Mean GBC	Mean Cholecystitis	EN	Lasso
**P19419**	ELK1	-0.42	-40.15	-83.42	3.12	0.03	585.32	625.47	TRUE	FALSE
**P09622**	DLDH	0.2	130.02	-165.43	425.47	0.03	1554.17	1424.15	FALSE	FALSE
**P41235**	HNF4A	-0.56	-3391.97	-6009.02	-774.91	0.01	25352.3	28744.26	TRUE	FALSE
**P54274**	TERF1	-0.38	-48.36	-109.26	12.54	0.65	488.35	536.71	TRUE	FALSE
**O75884**	RBBP9	0.07	11.91	-58.74	82.57	0.03	1201.73	1189.82	FALSE	FALSE
**P25440**	BRD2	0.35	98.68	-18.34	215.7	0.14	850.28	751.6	TRUE	FALSE
**P07196**	NFL	0.36	68.81	-13.81	151.43	0.07	618.04	549.23	TRUE	FALSE
**P01106**	c-Myc	-0.28	-613.3	-1664.67	438.07	0.91	320.97	934.27	TRUE	FALSE
**Q9NVQ4**	FAIM1	-0.35	-74.77	-172.37	22.82	0.09	727.23	554.1	TRUE	FALSE
**P09923**	Alkaline phosphatase, intestine	-0.21	-47.13	-148.1	53.84	0.07	680.15	727.28	TRUE	FALSE
**Q92956**	HVEM	-0.06	-16.28	-127.22	94.66	0.02	375.91	416.33	TRUE	FALSE
**P09238**	MMP-10	-0.37	-424.75	-953.15	103.65	0.27	2643.26	3068.01	TRUE	FALSE
**Q96A25**	T106A	-0.34	-58.61	-141.29	24.06	0.07	214.01	272.63	TRUE	FALSE
**Q8WU66**	TSEAR	-0.35	-82.09	-192.3	28.12	0.3	628.6	710.69	TRUE	FALSE
**O00238**	BMP RIB	-0.49	-22.29	-42.96	-1.61	0.02	344.1	366.38	TRUE	FALSE
**Q9H5K3**	SG196	-0.24	-13.75	-37.93	10.44	0.04	313.6	327.34	FALSE	FALSE
**Q9UNP4**	SIAT9	0.07	9.89	-47.46	67.24	0.02	349.6	339.71	FALSE	FALSE
**Q5VY80**	RET1L	-0.22	-67.67	-213.85	78.52	0.41	389.32	376.87	TRUE	FALSE
**Q9NZ53**	PDXL2	-0.11	-53.99	-262.09	154.1	0.05	515.67	569.66	FALSE	FALSE
**Q6UXG2**	K1324:ECD	-0.28	-7.62	-19.57	4.33	0.46	241.83	249.45	TRUE	FALSE
**Q6UXG2**	K1324:CD	-0.28	-7.62	-19.57	4.33	0.46	241.83	249.45	TRUE	FALSE
**Q8N9N2**	ASCC1	-0.2	-27.86	-90.08	34.36	0.59	1076.87	1104.73	TRUE	FALSE
**Q16401**	PSMD5	-0.26	-229.29	-637.95	179.36	0.03	609.58	838.87	FALSE	FALSE
**P0DMV8**	HSP 70	0.38	385.64	-52.92	824.2	0.13	1966.03	1822.48	TRUE	FALSE
**P30740**	Serpin B1	-0.38	-596.31	-1284.88	92.27	0.02	4781.01	5377.32	TRUE	FALSE
**P09603**	CSF-1	-0.11	-1.39	-6.93	4.15	0.96	1566.49	1500.55	TRUE	FALSE
**Q9Y5E7**	PCDB2	-0.36	-282.41	-664.31	99.49	0.01	469.04	751.45	TRUE	FALSE
**Q8IZP9**	GPR64	-0.45	-35.78	-72.39	0.84	0.02	471.68	507.45	TRUE	FALSE
**Q12918**	KLRB1	0.23	20.97	-17.22	59.17	0.03	601.82	748.33	TRUE	FALSE

**Q08554**	Desmocollin-1	-0.32	-30.85	-72.52	10.83	0.03	650.52	681.37	TRUE	FALSE
**Q9UL19**	TIG3	0.1	20.06	-69.93	110.05	0.8	1138.98	1118.92	TRUE	FALSE
**P05113**	IL-5	0.16	63.12	-96.63	222.88	0.2	1859.42	2023.6	TRUE	FALSE
**P04792**	HSP 27	-0.17	-448.48	-1632.15	735.18	0.62	4241.75	4690.23	TRUE	FALSE
**Q8TB22**	SPT20	0.16	702.95	-1112.22	2518.13	0.01	2153.08	1450.13	FALSE	FALSE
**Q9UGC7**	RF1ML	0.45	128.91	6.27	251.55	0.14	967.21	838.29	TRUE	FALSE
**P12109**	Collagen a1(VI)	0.12	79.71	-211.04	370.46	0.12	9964.57	11265.36	TRUE	FALSE
**Q92922**	SMRC1	0.17	943.48	-1328.05	3215.01	0.05	2174.77	1231.29	FALSE	FALSE
**Q9UBS3**	DNJB9	-0.24	-950.38	-2735.62	834.86	0.71	10974	11924.38	TRUE	FALSE
**Q8TAT2**	FGFP3	0.33	77	-26.35	180.34	0.05	1223.5	1146.51	TRUE	FALSE
**P20061**	Holo-TC I	0.11	56.59	-152.76	265.93	0.01	9913.41	11624.43	TRUE	FALSE
**P31415**	CASQ1	-0.01	-14.04	-840.82	812.74	0	7586.03	7600.06	FALSE	FALSE
**P0CG30**	GSTT2	0.43	27.29	0.09	54.5	0.05	427.84	400.55	TRUE	FALSE
**Q9UBS5**	GABR1	0.42	214.9	3.59	426.21	0.42	684	469.1	TRUE	FALSE
**Q8TEY5**	CR3L4	0.35	70	-14.2	154.21	0.43	936.56	866.56	TRUE	FALSE
**Q96EP1**	CHFR	0.09	14.62	-51.99	81.24	0.05	1033.44	1018.81	FALSE	FALSE
**P67870**	CSK2B	-0.29	-105.62	-278.25	67.01	0.74	485.23	590.85	TRUE	FALSE
**O75022**	LIRB3	-0.06	-10.54	-84.82	63.74	0.41	1296.87	2114.11	TRUE	FALSE
**Q96P66**	GP101	0.08	12.97	-52.5	78.44	0.04	480.79	546.44	TRUE	FALSE
**Q9NTX7**	RN146	-0.03	-11.4	-167.57	144.77	0.04	1322.49	1333.89	FALSE	FALSE
**Q8NEZ4**	KMT2C	0.01	1.83	-53.36	57.03	0.69	239.2	237.36	TRUE	FALSE
**P55199**	ELL	0.19	30.65	-39.21	100.52	0.72	1109.63	1078.98	TRUE	FALSE
**Q8TDQ0**	TIMD3	-0.28	-3061.04	-8294.8	2172.72	0.3	2678.87	2770.12	TRUE	FALSE
**O14791**	Apo L1	-0.28	-2440.25	-6188.95	1308.45	0.58	2246.53	2168.7	TRUE	FALSE
**Q3SXY7**	LRIT3	0.39	77.38	-5.1	159.87	0.24	432.63	355.25	TRUE	FALSE
**O95822**	DCMC	0.18	55.09	-89.03	199.21	0.03	760.95	705.86	FALSE	FALSE
**Q8N6T3**	ARFG1	-0.29	-94.79	-240.47	50.9	0.14	1158.99	1253.78	TRUE	FALSE
**P21506**	ZNF10	0.23	26.02	-23.01	75.05	0.19	990.77	964.74	TRUE	FALSE
**O43663**	PRC1	0.07	27.29	-131.68	186.26	0.02	286.76	259.47	FALSE	FALSE
**O43825**	B3GT2	-0.53	-75.17	-140.68	-9.65	0.05	1001.22	1076.39	TRUE	FALSE
**Q9ULW2**	Frizzled-10:CD	-0.11	-38.31	-194.97	118.34	0.01	447.87	557.94	TRUE	FALSE
**Q9ULW2**	Frizzled-10:FZ	-0.11	-38.31	-194.97	118.34	0.01	447.87	557.94	TRUE	FALSE
**Q8IZN3**	ZDH14	0.52	22.57	4.52	40.61	0.06	167.19	144.62	TRUE	FALSE
**Q9NZC2**	TREM2	0.41	422.93	-22.88	868.75	0.12	3373.96	2759.04	TRUE	FALSE
**P07738**	PMGE	-0.51	-142.89	-269.91	-15.86	0.06	539.2	682.09	TRUE	FALSE
**Q9UM13**	APC10	0.22	26.74	-24.18	77.67	0.41	845.71	818.97	TRUE	FALSE
**P06753**	Tropomyosin alpha-3 chain	-0.39	-56.54	-125.73	12.65	0.35	333.73	390.27	TRUE	FALSE
**Q15102**	PA1B3	-0.58	-2428.82	-4364.68	-492.97	0.02	8362.3	10791.13	TRUE	FALSE

**P16152**	CBR1	0.31	370.97	-140.51	882.45	0.03	2900.39	2529.43	FALSE	FALSE
**Q9P2N7**	KLH13	-0.51	-193.18	-372.51	-13.85	0.11	1621.55	1814.72	TRUE	FALSE
**Q9NZA1**	CLIC5	-0.47	-874.91	-1714.99	-34.83	0.14	5648.22	6523.12	TRUE	FALSE
**Q15631**	TSN	-0.47	-134.5	-265.15	-3.85	0.07	1138.8	1273.29	TRUE	FALSE
**Q9ULC4**	MCTS1	-0.64	-264.26	-449.37	-79.14	0	1459.85	1724.11	TRUE	FALSE
**O75347**	TBCA	0.43	322.45	-8.74	653.63	0.03	2797.65	2475.21	TRUE	FALSE
**Q9Y3E7**	CHMP3	0.33	1069.72	-326.45	2465.88	0.2	7637.82	6568.1	TRUE	FALSE
**Q96F10**	SAT2	-0.34	-150.88	-359.03	57.28	0.01	1243.08	1393.96	TRUE	FALSE
**P48643**	TCP-1-epsilon	-0.26	-9299.77	-25052.08	6452.54	0.35	54405.24	63705.01	TRUE	FALSE
**O43281**	EFS	-0.48	-47.98	-93.43	-2.52	0.04	733.99	781.97	TRUE	FALSE
**Q9NQT5**	Exosome component 3	-0.15	-300.77	-1166.32	564.79	0.04	1126.13	1426.9	FALSE	FALSE
**P53365**	ARFP2	-0.26	-949.19	-2740.63	842.24	0.26	1408.27	2357.47	TRUE	FALSE
**P18440**	ARY1	-0.65	-132.7	-222.06	-43.35	0	1212.38	1345.08	TRUE	TRUE
**P43378**	PTN9	-0.23	-10.09	-29.83	9.65	0.82	321.26	331.35	TRUE	FALSE
**O75815**	BCAR3:Ras-GEF	0.47	100.9	9.04	192.76	0.07	963.59	862.69	TRUE	FALSE
**O75815**	BCAR3:SH2	0.47	100.9	9.04	192.76	0.07	963.59	862.69	TRUE	FALSE
**Q96AT9**	RPE	-0.21	-173.81	-562.63	215.01	0.76	1216.17	1389.98	TRUE	FALSE
**Q07866**	KLC1	0.31	1134.44	-432.25	2701.14	0.3	5000.82	3866.37	TRUE	FALSE
**Q13630**	FCL	0.15	167.58	-302.6	637.77	0.02	944.68	777.1	FALSE	FALSE
**Q12923**	PTN13	-0.6	-25.99	-46.45	-5.53	0.21	202.88	228.87	TRUE	FALSE
**Q6YP21**	KAT3	-0.29	-112.02	-290.72	66.69	0.28	696.66	808.68	TRUE	FALSE
**Q9P0V9**	SEP10	-0.23	-1104.66	-3432.14	1222.82	0.55	2596.45	3701.11	TRUE	FALSE
**P50990**	TCP-1-theta	-0.23	-5452.36	-16114.04	5209.32	0.38	37558.86	43011.22	TRUE	FALSE
**Q9HCZ1**	ZN334	-0.43	-67.29	-136.97	2.39	0.13	1399.47	1466.77	TRUE	FALSE
**Q96ER9**	CCD51	0.36	70.67	-10.42	151.75	0.11	587.05	516.39	TRUE	FALSE
**O95989**	NUDT3	-0.18	-35.67	-129.36	58.01	0.8	409.95	445.63	TRUE	FALSE
**P14316**	IRF2	-0.38	-32.2	-70.87	6.47	0.13	291.38	323.58	TRUE	FALSE
**Q15849**	UT2	-0.19	-49.19	-168.7	70.31	0.97	1027.78	1076.98	TRUE	FALSE
**Q86VK4**	ZN410	0.27	11.3	-6.97	29.57	0.11	344.99	333.69	TRUE	FALSE
**O60942**	MCE1	-0.33	-366.97	-912.17	178.22	0.66	4127.81	3969.26	TRUE	FALSE
**P16150**	LEUK	-0.36	-175.59	-411.44	60.26	0.03	632.09	807.68	TRUE	FALSE
**Q8IY33**	MILK2	-0.35	-1202.4	-2841.97	437.17	0.13	1719.53	2921.93	TRUE	FALSE
**Q8IYJ3**	SYTL1	-0.32	-29.99	-73.58	13.61	0.26	458.32	488.31	TRUE	FALSE
**Q9Y6T7**	DGKB	-0.47	-128.43	-250.18	-6.69	0.02	1802.75	1931.19	TRUE	FALSE
**P56278**	MTCP1	0.31	47.26	-17.21	111.73	0.07	863.17	968.92	TRUE	FALSE
**Q8IWT3**	CUL9	-0.32	-8.53	-19.89	2.83	0.04	227.2	235.73	FALSE	FALSE
**O14683**	P5I11	-0.31	-1014.26	-2500.87	472.36	0.25	4100.01	5114.26	TRUE	FALSE
**Q6P2E9**	EDC4	0.31	129.04	-51.15	309.24	0.25	2738.84	2609.79	TRUE	FALSE

**Q13976**	KGP1B	-0.32	-86.03	-203.58	31.52	0.15	1962.95	2048.99	TRUE	FALSE
**P35070**	BTC	0.33	100.8	-24.85	226.44	0.49	473.38	372.58	TRUE	FALSE
**Q9BXY4**	RSPO3	0.33	65.76	-20.44	151.96	0.06	766.93	703.76	TRUE	FALSE
**O43915**	VEGF-D	-0.09	-245.58	-1517.13	1025.97	0.74	1142.72	1300.67	TRUE	FALSE
**Q9BQB4**	SOST	0.18	75.23	-109.61	260.08	0.41	521.18	485.68	TRUE	FALSE
**P10451**	Osteopontin	0.44	71.34	0.36	142.33	0.03	779.89	708.54	TRUE	FALSE
**P41221**	WNT5A	-0.33	-172.85	-400.47	54.77	0.04	1312.78	1485.62	TRUE	FALSE
**Q8N8Q1**	C56D1	-0.55	-259.04	-481.13	-36.95	0.03	1721.86	1980.9	TRUE	FALSE
**O15263**	HBD-2	0.12	958.54	-2375.62	4292.7	0.98	9839.7	8881.16	TRUE	FALSE
**P20155**	2 ISK	0.06	8.23	-50.2	66.67	0.28	383.91	375.67	TRUE	FALSE
**Q8TEU8**	WFKN2	0.18	79.92	-120.48	280.33	0.23	2074.74	1994.81	TRUE	FALSE
**Q15427**	SF3B4	-0.48	-23.11	-45.64	-0.59	0.05	332.88	355.99	TRUE	FALSE
**P0DJ93**	SIM13	0.09	6.63	-26.18	39.44	0.56	593.92	587.29	TRUE	FALSE
**Q9NXH8**	CI167	0.45	29.73	1.31	58.15	0.07	606.68	576.94	TRUE	FALSE
**A8MVW 0**	F1712	-0.39	-28.94	-62.21	4.34	0.05	913.81	942.74	TRUE	FALSE
**Q93084**	AT2A3	-0.73	-143.42	-235.08	-51.76	0	1335.81	1479.23	TRUE	TRUE
**Q9UH65**	SWP70	0.5	128.25	18.23	238.28	0.06	1559.91	1431.66	TRUE	FALSE
**Q8IWE4**	DCNL3	0.28	195.46	-92.96	483.88	0.05	740.86	545.4	TRUE	FALSE
**P09211**	Glutathione S-transferase Pi	0.31	132.1	-49	313.2	0.18	3035.13	2903.03	TRUE	FALSE
**O76050**	NEUL1	-0.28	-40.89	-104.82	23.05	0.04	1012.28	1053.17	FALSE	FALSE
**P43363**	MAGE-10	-0.36	-345.71	-766.39	74.96	0.13	5361.17	5706.88	TRUE	FALSE
**P08246**	Elastase	-0.39	-3843.81	-8164.18	476.57	0.02	33753.52	37597.32	TRUE	FALSE
**P19784**	22,00 CSK	-0.3	-13.61	-33.57	6.35	0.99	744.7	1110.41	TRUE	FALSE
**P11926**	Ornithine decarboxylase	-0.39	-70.38	-151.66	10.89	0.16	1320.13	1390.51	TRUE	FALSE
**P07355**	annexin II	0.32	1616.54	-555	3788.08	0.12	964.3	865.05	TRUE	FALSE
**P05155**	C1-Esterase Inhibitor	-0.02	-71.79	-1510.13	1366.56	0.12	5919.91	5544.22	TRUE	FALSE
**Q15485**	FCN2	-0.37	-2647.9	-5732.95	437.16	0.04	57023.67	70010.83	TRUE	FALSE
**P24158**	Proteinase-3	-0.18	-170.52	-569.16	228.11	0	1424.98	1907.94	TRUE	FALSE
**P47736**	RPGP1	-0.39	-81.54	-179.28	16.2	0.02	571.27	652.81	TRUE	FALSE
**P08476**	Inhibin bA chain	0.03	8.15	-107.66	123.95	0.11	691.72	620.27	TRUE	FALSE
**P08476**	Activin A	0.03	8.15	-107.66	123.95	0.11	691.72	620.27	TRUE	FALSE
**P08833**	IGFBP-1	0.51	4259.46	566.5	7952.43	0.01	17101	12841.53	TRUE	FALSE
**Q9GZX6**	IL-22	-0.59	-432.85	-783.13	-82.56	0.02	1701.24	2134.08	TRUE	FALSE
**Q96P47**	AGAP3	0.27	63.02	-35.1	161.14	0.96	669.98	606.97	TRUE	FALSE
**Q08257**	QOR	0.25	1006.24	-833.92	2846.4	0	5054.94	4048.69	FALSE	FALSE
**Q9UHL0**	DDX25	-0.06	-5.58	-45.55	34.39	0.02	422.79	428.37	FALSE	FALSE

**Q92844**	TANK	0.27	413.42	-233.11	1059.96	0.55	2346.75	1933.32	TRUE	FALSE
**Q07011**	4-1BB	-0.36	-25.44	-58	7.11	0.01	440.64	466.09	TRUE	FALSE
**Q96PD4**	IL-17F	0.42	62.42	0.55	124.29	0.02	333.4	453.14	TRUE	FALSE
**P01215**	Glycoprotein hormones a-chain	-0.14	-19.87	-86.64	46.89	0.01	2310.8	1666.12	TRUE	FALSE
**P02747**	C1QC	-0.17	-112.17	-403.92	179.58	0.04	1150.52	1262.69	FALSE	FALSE
**Q16663**	MIP-5	0.51	253.46	41.87	465.06	0.04	1636	1382.54	TRUE	FALSE
**P26447**	S100A4	-0.22	-47.08	-139.88	45.73	0.47	665.52	728.68	TRUE	FALSE
**P27930**	IL-1 sRII	0.03	44.48	-583.94	672.89	1	422.63	520.79	TRUE	FALSE
**Q9NZH7**	IL-1F8	-0.37	-48.52	-107.9	10.86	0.01	623.58	672.1	TRUE	FALSE
**P52797**	Ephrin-A3	-0.21	-42.91	-139.17	53.35	0.05	2232.94	2754.76	FALSE	FALSE
**O75626**	PRDM1	-0.36	-52.99	-118.84	12.86	0.18	867.59	920.58	TRUE	FALSE
**P14649**	MYL6B	-0.19	-33.11	-114.6	48.39	0.65	642.16	675.27	TRUE	FALSE
**P29074**	PTN4	-0.58	-175.68	-312	-39.36	0.02	2130.5	2306.18	TRUE	FALSE
**Q9BYN0**	SRXN1	0.39	57.67	-5.24	120.59	0.39	478.13	420.45	TRUE	FALSE
**P31943**	HNRH1	-0.01	-10.66	-409.36	388.04	0	2549.44	2560.1	FALSE	FALSE
**O43617**	TPPC3	-0.48	-145.96	-284.24	-7.68	0.07	1200.46	1346.42	TRUE	FALSE
**P17655**	CAN2	0.41	496.64	-21.02	1014.31	0.07	4119.6	3622.96	TRUE	FALSE
**P35236**	PTN7	0.45	39.4	2.42	76.37	0.09	303.66	264.27	TRUE	FALSE
**Q15828**	Cystatin M	-0.58	-4290.66	-7581.86	-999.46	0.01	4452.36	5491.6	TRUE	FALSE
**O75462**	CRLF1	-0.04	-13.81	-163.96	136.34	0.01	423.93	473.2	TRUE	FALSE
**Q9H6E4**	CC134	0.05	72.42	-580.66	725.51	0.18	273.39	301.54	TRUE	FALSE
**Q9Y281**	COF2	-0.32	-110.47	-264.98	44.04	0.12	511.3	621.76	TRUE	FALSE
**P01042**	Kininogen, HMW, Two Chain	0.13	491.66	-1084.86	2068.18	0.33	3328.97	3512.56	TRUE	FALSE
**P01042**	Kininostatin	0.13	491.66	-1084.86	2068.18	0.33	3328.97	3512.56	TRUE	FALSE
**P01042**	Kininogen, HMW	0.13	491.66	-1084.86	2068.18	0.33	3328.97	3512.56	TRUE	FALSE
**Q8N8U9**	BMPER	0.33	131.27	-38.11	300.64	0.32	1686.05	1554.79	TRUE	FALSE
**P15086**	Carboxypeptidase B1	0.16	484.39	-832.24	1801.03	0.26	3067.36	2582.97	TRUE	FALSE
**P12104**	FABP2	0.41	286.61	-7.32	580.55	0.13	1731.22	1444.6	TRUE	FALSE
**P55789**	HERV1	0.25	105.09	-79.37	289.55	0.3	1323.79	1218.7	TRUE	FALSE
**P05014**	IFNA4	0.26	14.29	-9.06	37.63	0.96	308.56	294.28	TRUE	FALSE
**P00338**	LDHA	0.14	1592.31	-3225.93	6410.55	0.23	728.32	839.55	TRUE	FALSE
**P16519**	NEC2	-0.24	-867.14	-2629.36	895.08	0.05	3298.9	3103.55	TRUE	FALSE
**Q02297**	SMDF	0.07	63.73	-338.45	465.91	0.54	190.9	161.32	TRUE	FALSE
**Q02297**	NEUREGULIN-1	0.07	63.73	-338.45	465.91	0.54	190.9	161.32	TRUE	FALSE
**P01732**	CD8A	-0.66	-2730.88	-4570.98	-890.78	0.01	1371.73	1631.3	TRUE	TRUE
**P01732**	CD8A	-0.66	-2730.88	-4570.98	-890.78	0.01	12890.82	15621.7	TRUE	FALSE

**Q6UW15**	REG3G	0.18	1478.58	-1914.26	4871.42	0.41	506.92	568.95	TRUE	FALSE
**P19801**	ABP1	-0.37	-705.89	-1554.02	142.23	0.07	3068.27	3774.16	TRUE	FALSE
**P54802**	NAG	-0.37	-679.49	-1483.62	124.64	0.05	5396.89	6076.38	TRUE	FALSE
**P06276**	Pseudocholinesterase	-0.62	-1165.52	-1986.26	-344.78	0	55717.04	64338.51	TRUE	FALSE
**P63098**	Calcineurin B a	0.38	378.68	-55.01	812.37	0.17	5112.08	4733.39	TRUE	FALSE
**Q07075**	AMPE	-0.64	-357.55	-603.93	-111.18	0	2260.21	2617.76	TRUE	FALSE
**Q8WXI7**	CA125	-0.09	-16.6	-105.36	72.15	0	102.98	119.58	FALSE	FALSE
**P20023**	Complement receptor type 2	-0.46	-1056.65	-2099.39	-13.91	0.06	4814.11	5870.76	TRUE	FALSE
**P08887**	IL-6 sRa	-0.61	-372.05	-647.64	-96.46	0.01	10069.52	11292.57	TRUE	FALSE
**P17301**	Integrin alpha-2	0.39	55.13	-3.29	113.54	0.14	326.21	271.09	TRUE	FALSE
**Q9HAP6**	LIN7B	0.02	2.92	-66.21	72.05	0.01	870.03	395.61	FALSE	FALSE
**P02753**	RBP	-0.49	-414.5	-784.68	-44.33	0.02	25739.17	28642.1	TRUE	FALSE
**P43251**	Biotinidase	-0.01	-5.17	-183.73	173.39	0.35	13135.02	13674.02	TRUE	FALSE
**Q03692**	COAA1	0.37	212.56	-27.53	452.66	0.01	747.24	534.67	TRUE	FALSE
**P15056**	BRAF1	-0.27	-94.89	-258.15	68.38	0.39	1346.32	1441.21	TRUE	FALSE
**Q9UBU2**	2,00 DKK	0.3	1917.66	-794.78	4630.11	0	682.2	574.24	TRUE	TRUE
**Q9UBU2**	2,00 DKK	0.3	1917.66	-794.78	4630.11	0.59	17335.92	15418.26	TRUE	FALSE
**P35916**	VEGF sR3	-0.37	-527.06	-1154.32	100.2	0.08	6392.57	6919.63	TRUE	FALSE
**O75473**	LGR5	-0.36	-55.95	-125.71	13.81	0.19	812.34	868.29	TRUE	FALSE
**Q9BXB1**	LGR4	0.28	915.73	-454.94	2286.39	0.49	2412.87	1497.14	TRUE	FALSE
**P55285**	Cadherin-6	0.02	2.82	-54.9	60.53	0.1	1628.13	1707.75	TRUE	FALSE
**Q13158**	FADD	-0.48	-182.31	-348.57	-16.06	0.02	2976.25	3158.57	TRUE	FALSE
**Q86SX6**	GLRX5	-0.44	-60.54	-123.24	2.16	0.02	268.53	329.07	TRUE	FALSE
**O75056**	SDC3	-0.36	-166.13	-370.5	38.24	0.22	1689.03	1855.16	TRUE	FALSE
**Q2MKA7**	RSPO1	0.49	341.43	37.57	645.28	0.01	2344.11	2002.68	TRUE	FALSE
**P55107**	BMP-3b	0.25	48.58	-35.57	132.73	0.28	1398.35	1349.76	TRUE	FALSE
**P16860**	BNP	0.04	4.03	-35.94	43.99	0.03	481.26	460.29	TRUE	FALSE
**P16860**	BNP-32	0.04	4.03	-35.94	43.99	0.03	481.26	460.29	TRUE	FALSE
**P16860**	N-terminal pro-BNP	0.04	4.03	-35.94	43.99	0.03	481.26	460.29	TRUE	FALSE
**Q7Z4P5**	GDF7	0.34	45.57	-10.98	102.12	0.59	417.54	382.46	TRUE	FALSE
**Q01523**	HD-5	0.5	592.9	78.53	1107.26	0.03	3500.52	2907.63	TRUE	FALSE
**P30043**	BLVRB	-0.62	-11219.94	-19470.07	-2969.81	0.02	44542.11	55762.05	TRUE	FALSE
**O15075**	DCAK1	-0.27	-294.92	-801.02	211.18	0.68	2890.78	3185.7	TRUE	FALSE
**P12429**	Annexin III	-0.44	-168.95	-354.32	16.42	0.04	235.36	404.3	TRUE	FALSE
**P52564**	MP2K6	-0.21	-22.11	-70.74	26.52	0.76	621.35	643.46	TRUE	FALSE
**P13796**	L-plastin	-0.28	-888.71	-2274.74	497.32	0.04	19879.48	20768.19	FALSE	FALSE

**Q96MU8**	KREM1	0.12	23.84	-62.5	110.18	0.53	3213.13	3079.79	TRUE	FALSE
**Q70SY1**	CR3L2	0.37	24.17	-3.63	51.96	0.46	223.58	199.41	TRUE	FALSE
**O15079**	SNPH	0.28	47.51	-24.6	119.62	0.04	520.12	472.61	FALSE	FALSE
**P13804**	ETFA	-0.46	-117.44	-230.44	-4.44	0.03	1969.41	2086.85	TRUE	FALSE
**P21926**	CD9	-0.32	-115.8	-292.45	60.85	0.13	576.65	692.45	TRUE	FALSE
**P15328**	FOLR1	-0.47	-101.87	-203.48	-0.26	0.07	696.54	798.41	TRUE	FALSE
**Q9UNL4**	ING4	-0.21	-28.44	-88.15	31.27	0.01	533.15	561.59	FALSE	FALSE
**Q92598**	HS105	0.54	65.51	13.37	117.65	0.02	635.87	570.36	TRUE	FALSE
**Q9Y6N9**	USH1C	-0.26	-22.69	-62.52	17.14	0.21	386.9	409.59	TRUE	FALSE
**Q96EY8**	MMAB	0.27	675.51	-419.87	1770.88	0.36	5058.86	4383.36	TRUE	FALSE
**P37235**	HPCL1	-0.28	-95.78	-256.16	64.61	0.51	951.36	1042.93	TRUE	FALSE
**Q9NVS9**	PNPO	0.41	64.21	-2.55	130.97	0.12	1072.05	1007.84	TRUE	FALSE
**Q9BXS1**	IDI2	0.13	16.3	-41.23	73.83	0.4	307.29	290.99	TRUE	FALSE
**Q96FQ6**	S100A16	0.19	100.73	-129.61	331.06	0.47	2345.87	2245.15	TRUE	FALSE
**P49798**	RGS4	-0.25	-80.1	-235.33	75.12	0.03	781.67	834.71	TRUE	FALSE
**Q9Y294**	ASF1A	-0.3	-226.67	-588.08	134.73	0.23	870.12	1096.8	TRUE	FALSE
**Q96F85**	CB032	-0.59	-1212	-2149.7	-274.29	0.03	5767.72	6979.71	TRUE	FALSE
**Q9UKY0**	PRND	-0.56	-214.4	-382.38	-46.42	0.01	2156.87	2371.27	TRUE	FALSE
**A6NDG6**	PGP	-0.21	-159.65	-507.88	188.59	0.64	2576.86	2736.51	TRUE	FALSE
**Q04837**	SSB	0.49	518.81	75.89	961.74	0.01	1459.98	941.17	TRUE	FALSE
**Q9P287**	BCCIP	0.25	21.01	-15.32	57.34	0.5	991.41	970.4	TRUE	FALSE
**Q9H2U2**	PPase 2	0.29	73.24	-35.58	182.06	0.29	1071.55	998.31	TRUE	FALSE
**Q14194**	DRP-1	-0.3	-39.44	-101.91	23.02	0.59	344.29	383.73	TRUE	FALSE
**Q96G03**	PGM2	-0.27	-65.72	-178.2	46.75	0.64	995.21	1060.93	TRUE	FALSE
**P04062**	GLCM	0.21	309.07	-330.24	948.38	0.31	5430.15	5121.08	TRUE	FALSE
**P21964**	Catechol O-methyltransferase	-0.34	-77.69	-182.29	26.92	0.22	1312.46	1390.14	TRUE	FALSE
**P84085**	ARF5	-0.02	-4.41	-88.46	79.64	0.82	820.24	824.65	TRUE	FALSE
**Q9H0F7**	6,00 ARL	0.35	406.34	-84.31	896.99	0.39	2782.04	2375.71	TRUE	FALSE
**P62380**	TBPL1	0.12	20.14	-56.1	96.37	0.66	560.76	540.62	TRUE	FALSE
**Q96EQ0**	SGTB	-0.32	-434.04	-1089.9	221.82	0.01	455.94	889.98	TRUE	FALSE
**P62837**	UB2D2	0.22	17.91	-17.64	53.46	0.12	631.88	613.97	TRUE	FALSE
**P35030**	3,00 TRY	0.23	582.79	-493.49	1659.07	0.1	360.97	396.96	TRUE	FALSE
**Q9BQI0**	AIF1L	0.35	125.51	-31.69	282.7	0.09	1851.54	1726.03	TRUE	FALSE
**P49715**	CEBPA	0.18	220.28	-283.52	724.08	0.05	517.15	296.87	FALSE	FALSE
**Q9H4D0**	CSTN2	0.65	90.29	31.28	149.3	0	564.27	473.98	TRUE	TRUE
**A6NFH5**	FBP12	-0.3	-100.7	-264.56	63.17	0.26	828.54	929.24	TRUE	FALSE
**P32456**	GBP2	0.42	155.52	-7.7	318.75	0.04	1870.86	1715.34	TRUE	FALSE
**Q9Y3E1**	HDGR3	0.3	28.76	-12.45	69.97	0.19	328.48	299.72	TRUE	FALSE

**O94813**	SLIT2	0.21	209.7	-216.38	635.79	0.4	4038.96	3829.26	TRUE	FALSE
**O75094**	SLIT3	0.37	159.33	-29.62	348.27	0.04	2243.14	2083.82	TRUE	FALSE
**Q9HAN9**	NMNA1	-0.49	-109.38	-214.52	-4.25	0.46	543.34	652.73	TRUE	FALSE
**P49221**	TGM4	-0.29	-21.07	-53.44	11.29	0.03	370.72	391.8	TRUE	FALSE
**Q8NI38**	IKBD	-0.31	-512.72	-1303.66	278.22	0.03	1715.83	2228.55	TRUE	FALSE
**Q02252**	MMSA	0.2	1477.7	-1721.23	4676.62	0.5	9223.7	7746	TRUE	FALSE
**Q8N565**	MREG	-0.63	-74.96	-128.39	-21.52	0.01	854.42	929.37	TRUE	FALSE
**Q9HCU8**	DNA polymerase subunit delta 4	-0.28	-63.59	-174.1	46.92	0.19	409.97	473.55	TRUE	FALSE
**O14933**	UB2L6	0.22	23.68	-25.28	72.65	0.02	802.36	778.68	FALSE	FALSE
**P36639**	8ODP	-0.41	-69.55	-150.72	11.62	0.04	644.17	713.71	TRUE	FALSE
**Q92597**	NDRG1	0.3	372.16	-148.1	892.41	0.4	1427.07	1054.92	TRUE	FALSE
**Q16775**	GLO2	-0.43	-5088.64	-10407.73	230.45	0.08	41887.21	46975.85	TRUE	FALSE
**O14958**	CASQ2	0.11	17.99	-51.8	87.78	0.12	418.81	400.82	TRUE	FALSE
**Q01449**	MLRA	-0.56	-568.22	-1024.93	-111.51	0.02	4255.16	4823.37	TRUE	FALSE
**Q6UWS5**	PT117	-0.41	-30.24	-63.06	2.59	0.07	533.88	564.12	TRUE	FALSE
**Q86WC4**	OSTM1	-0.34	-7.1	-16.16	1.96	0.03	293.85	300.96	TRUE	FALSE
**O95897**	NOE2	-0.39	-267.15	-578.33	44.03	0.03	24573.57	27861.95	TRUE	FALSE
**Q9BW66**	CINP	-0.37	-371.24	-861.15	118.67	0.52	682.02	1053.26	TRUE	FALSE
**P55289**	Cadherin-12:ECD	0.21	56.52	-59.02	172.06	0.02	2071.17	791.17	TRUE	FALSE
**P55289**	Cadherin-12:CD	0.21	56.52	-59.02	172.06	0.02	2071.17	791.17	TRUE	FALSE
**Q16623**	STX1a	-0.23	-259.2	-809.49	291.09	0.56	430.97	418.94	TRUE	FALSE
**Q86Z14**	KLOTB	-0.44	-128.73	-259.82	2.37	0.05	717.89	846.61	TRUE	FALSE
**P00533**	EGFRvIII	0.17	869.47	-1268.07	3007.02	0.12	11585.47	12290.21	TRUE	FALSE
**P00533**	ERBB1	0.17	869.47	-1268.07	3007.02	0.12	11585.47	12290.21	TRUE	FALSE
**P24593**	IGFBP-5	-0.07	-131.24	-967.55	705.07	0	53106.23	57784.16	TRUE	FALSE
**Q96DX5**	ASB9	0.32	321.09	-117.59	759.76	0.13	2965.96	2644.88	TRUE	FALSE
**P08865**	40S ribosomal protein SA	-0.43	-70.98	-148.56	6.61	0.25	815.33	886.31	TRUE	FALSE
**P10997**	IAPP	0.26	188.82	-127.04	504.67	0.31	2616.4	2427.59	TRUE	FALSE
**Q6UX27**	VSTM1	-0.41	-92.95	-193.58	7.68	0.04	1472.06	1565.01	FALSE	FALSE
**P15085**	CBPA1	0.22	310.83	-286.19	907.85	0.94	3035.87	2725.04	TRUE	FALSE
**Q712K3**	UB2R2	-0.5	-141.05	-266.49	-15.6	0.05	2265.72	2406.77	TRUE	FALSE
**O75897**	ST1C4	0.31	30.53	-10.43	71.49	0.58	274.89	244.36	TRUE	FALSE
**Q86WK6**	AMGO1:ECD	-0.04	-3.03	-37.97	31.9	0.04	306.01	309.05	FALSE	FALSE
**Q86WK6**	AMGO1:CD	-0.04	-3.03	-37.97	31.9	0.04	306.01	309.05	FALSE	FALSE
**Q92630**	DYRK2	-0.23	-5.67	-16.38	5.03	0.02	211.25	216.92	FALSE	FALSE
**Q96PP8**	GBP5	-0.36	-142.79	-320.31	34.73	0.15	1721.58	1864.37	TRUE	FALSE

**O95045**	UPP2	0.36	31.47	-5.3	68.25	0.19	490.92	459.44	TRUE	FALSE
**Q969E8**	TSR2	-0.31	-123.27	-303.94	57.39	0.38	1350.25	1473.52	TRUE	FALSE
**Q6PID6**	TTC33	-0.33	-1155.81	-2867.09	555.47	0.04	3067.85	4223.66	TRUE	FALSE
**Q03014**	HHEX	-0.42	-59.36	-125.92	7.2	0.17	683.81	743.18	TRUE	FALSE
**P23582**	Natriuretic Peptide C-Type	0.25	13.34	-9.58	36.27	0.21	479.17	465.82	TRUE	FALSE
**A6NKN8**	PC4L1	-0.15	-26.25	-103.25	50.75	0.04	332.36	358.61	FALSE	FALSE
**Q68CL5**	TPGS2	-0.53	-138.37	-253.4	-23.35	0.02	2812.98	2951.35	TRUE	FALSE
**Q9UKA8**	RCAN3	-0.47	-675.01	-1309.87	-40.15	0.05	5823.76	6498.77	TRUE	FALSE
**Q9BW30**	TPPP3	0.24	219.1	-176.49	614.69	0.36	2943.44	2724.34	TRUE	FALSE
**Q9NY72**	SCN3B	-0.49	-48.01	-93.64	-2.37	0.07	337.04	385.05	TRUE	FALSE
**Q9Y6N8**	CAD10	-0.46	-16.97	-33.39	-0.56	0.07	252.84	269.82	TRUE	FALSE
**Q8NFR9**	I17RE	-0.29	-114.34	-294.06	65.38	0.46	1310.33	1424.67	TRUE	FALSE
**P59666**	HNP-3	-0.43	-1481.13	-3015.48	53.21	0.03	5485.4	6966.54	TRUE	FALSE
**Q9NTN9**	SEM4G	-0.39	-182.95	-389.14	23.24	0.12	2042.16	2225.11	TRUE	FALSE
**P29016**	CD1B	-0.24	-895.15	-2692.79	902.5	0.03	144.85	1039.99	TRUE	FALSE
**P01303**	NPY	0.43	132.26	0.43	264.1	0.09	1643.04	1510.77	TRUE	FALSE
**Q9Y2C3**	B3GT5	-0.11	-56.07	-296.91	184.77	0.04	1368.34	1424.41	FALSE	FALSE
**Q9Y4U1**	MMAC	-0.24	-197.23	-553.78	159.32	0.01	2381.03	2578.26	FALSE	FALSE
**Q9NZV6**	MSRB1	-0.45	-68.29	-138.57	1.98	0.1	809.35	877.65	TRUE	FALSE
**Q8TA86**	RP9	0.27	86.97	-55.85	229.79	0.27	1635.06	1548.09	TRUE	FALSE
**Q5SZJ8**	BEND6	-0.33	-20.88	-50.15	8.38	0.02	299.47	320.35	FALSE	FALSE
**Q8WUW1**	BRK1	-0.35	-82.81	-189.11	23.5	0.14	1397.22	1480.03	TRUE	FALSE
**A6NIH7**	U119B	-0.21	-657.54	-2166.22	851.15	0.94	1816.62	2474.16	TRUE	FALSE
**Q96A32**	MLRS	-0.31	-406.03	-1003.31	191.25	0.3	3075.34	3481.37	TRUE	FALSE
**P24941**	CDK2	-0.13	-185.94	-827.69	455.81	0.55	367.43	358.22	TRUE	FALSE
**O00141**	SGK1	-0.4	-33.01	-68.24	2.22	0.01	782.35	815.37	TRUE	FALSE
**O00294**	TULP1	-0.37	-174.32	-405.1	56.45	0.01	184.84	359.16	TRUE	FALSE
**A2RU49**	HYKK	0.33	299.48	-74.73	673.68	0.26	951.48	652.01	TRUE	FALSE
**Q8IUE1**	TF2LX	0.34	55.33	-12.58	123.23	0.72	226.16	170.84	TRUE	FALSE
**Q9H1K6**	MESD1	-0.64	-481.85	-828.27	-135.43	0.02	2389.72	2871.57	TRUE	FALSE
**Q9P2M1**	LR2BP	-0.01	-4.53	-162.01	152.95	0.01	1197.45	1201.98	FALSE	FALSE
**Q6QNY1**	BL1S2	-0.4	-80.55	-178.77	17.67	0.23	168.41	248.96	TRUE	FALSE
**Q9H596**	DUS21	0.43	29.63	-0.16	59.41	0.07	377.21	347.58	TRUE	FALSE
**Q14863**	PO6F1	-0.12	-522.58	-2349.51	1304.35	0.03	6383.34	6905.93	FALSE	FALSE
**Q9GZZ9**	UBA5	-0.36	-29.39	-65.99	7.22	0.11	487.93	517.32	TRUE	FALSE
**P21579**	SYT1	-0.53	-60.46	-114.11	-6.82	0.02	406.17	466.63	TRUE	FALSE
**Q6ZN17**	LN28B	-0.01	-1.07	-38.88	36.75	0.03	338.88	339.95	FALSE	FALSE
**Q8WUP2**	FBLI1	0.49	669.05	99.61	1238.48	0.29	1601.19	932.15	TRUE	FALSE

**Q96BD8**	SKA1	-0.18	-24.68	-82.32	32.95	0.04	776.68	801.36	FALSE	FALSE
**Q8IUF8**	MINA	0.45	43.48	2.69	84.27	0.04	348.75	305.27	TRUE	FALSE
**Q8TBN0**	R3GEF	-0.19	-517.47	-1774.26	739.31	0.04	1120.42	1637.89	FALSE	FALSE
**P0DN86**	b-CF	0.43	376.72	9.85	743.59	0.07	1770.11	1393.39	TRUE	FALSE
**Q16584**	M3K11	-0.29	-1320.36	-3443.76	803.05	0.04	7889.32	9209.68	TRUE	FALSE
**Q8WYN0**	ATG4A	-0.37	-38.39	-84.13	7.34	0.11	416.08	454.47	TRUE	FALSE
**Q969S2**	NEIL2	0.4	11.22	-0.54	22.99	0.08	152.28	141.06	TRUE	FALSE
**Q15846**	CLUL1	0.35	102.82	-21.39	227.03	0.38	1342.79	1239.98	TRUE	FALSE
**Q99807**	COQ7	0.19	115.6	-144.83	376.03	0.05	1289.52	1173.92	FALSE	FALSE
**Q96Q40**	CDK15	-0.36	-341.83	-763.19	79.52	0.08	5521.67	5863.51	TRUE	FALSE
**O60888**	CUTA	-0.28	-39.06	-100.37	22.25	0.03	795.39	834.45	FALSE	FALSE
**Q8NFZ3**	NLGNY	0.3	14.72	-5.48	34.91	0.45	223.14	208.42	TRUE	FALSE
**O14514**	BAI1	-0.28	-16.55	-42.26	9.16	0.04	286.85	303.4	TRUE	FALSE
**O75191**	XYLB	-0.33	-138.49	-336.38	59.39	0.83	766.63	905.13	TRUE	FALSE
**P10321**	HLA-C	0.23	3171.07	-2756.35	9098.5	0.53	20077.65	16906.58	TRUE	FALSE
**P18615**	NELFE	0.18	275.73	-343.44	894.89	0.02	1060.85	785.13	FALSE	FALSE
**P29508**	SCCA1	0.43	168.53	2.06	335	0.03	1400.45	1231.92	TRUE	FALSE
**P24539**	AT5F1	-0.38	-72.72	-162.92	17.48	0.62	2113.04	2248.94	TRUE	FALSE
**P35226**	BMI-1	-0.27	-146.27	-410.98	118.44	0.07	422.88	569.15	TRUE	FALSE
**Q9UBR1**	BUP1	0.16	51.32	-89.91	192.55	0.01	733.89	682.57	FALSE	FALSE
**Q8IZL9**	CDK20	0.15	32.01	-65.51	129.53	0.02	705.42	673.41	FALSE	FALSE
**P01033**	TIMP-1	0.31	451.19	-191.6	1093.97	0.14	7406.27	6955.08	TRUE	FALSE
**O60519**	CRBL2	-0.45	-23.99	-48.56	0.58	0.03	396.83	420.82	TRUE	FALSE
**P32242**	OTX1	0.11	42.37	-111.18	195.92	0.03	371.62	329.26	FALSE	FALSE
**Q96QV6**	H2A1A	0.46	1335.14	103.54	2566.74	0.1	4890.14	3555.01	TRUE	FALSE
**O60814**	H2B1K	0.44	557.63	23.12	1092.14	0.07	1972.76	1415.13	TRUE	FALSE
**Q96FA3**	PELI1	-0.35	-61.73	-145.94	22.47	0.02	221.3	283.03	TRUE	FALSE
**A2RU54**	HMX2	-0.74	-70.74	-115.14	-26.35	0.01	581.22	651.96	TRUE	TRUE
**Q99081**	HTF4	-0.27	-735.1	-1964.19	493.98	0.18	4835.99	5571.09	TRUE	FALSE
**Q6ZST4**	LCNL1	0.42	44.86	0.34	89.38	0.04	283.1	238.24	TRUE	FALSE
**Q9P086**	MED11	-0.28	-169.37	-452.76	114.01	0.05	1094.11	1263.48	TRUE	FALSE
**P29558**	RBMS1	0.42	254.9	-1.63	511.43	0.19	1617.93	1363.02	TRUE	FALSE
**Q9NNX6**	DC-SIGN	0.11	64.28	-185.13	313.69	0.15	2522.15	2200.3	TRUE	FALSE
**P58400**	NRX1B	0.31	13.78	-5.33	32.88	0.27	296.89	283.11	TRUE	FALSE
**Q9UF33**	EPHA6	0.16	326.78	-570.9	1224.47	0.45	7942.85	7616.07	TRUE	FALSE
**Q9HBM1**	SPC25	-0.44	-259.21	-525.76	7.35	0.03	1686.07	1945.28	TRUE	FALSE
**Q5MJ09**	SPXN3	-0.23	-15.58	-45.68	14.51	0.2	281.46	297.05	TRUE	FALSE
**Q9H0W7**	THAP2	0.26	296.7	-181.33	774.74	0.05	1289.24	992.53	FALSE	FALSE
**P10828**	THB	0.59	389	110.5	667.49	0.02	3032.2	2643.2	TRUE	FALSE

**Q9UBT3**	Dkk-4	0.13	15.86	-39.03	70.76	0.99	7988.56	7173.85	TRUE	FALSE
**Q13323**	BIK	-0.47	-882.15	-1780.64	16.33	0.02	1118.12	2000.28	TRUE	FALSE
**P29376**	LTK	-0.53	-38.41	-72.13	-4.69	0.03	409.67	448.08	TRUE	FALSE
**Q9HAE3**	EFCB1	0.29	100.12	-44.5	244.74	0.82	901.18	801.06	TRUE	FALSE
**Q8WVD5**	RN141	0.44	146.41	7.81	285.02	0.08	927.91	781.5	TRUE	FALSE
**Q5T4F7**	SARP-3	0.35	555.92	-112.08	1223.93	0.31	2769.37	2213.44	TRUE	FALSE
**Q6UWP2**	DHR11	-0.35	-519.5	-1189.73	150.73	0.14	4366.96	4886.46	TRUE	FALSE
**Q96MC5**	CP045	0.31	612.42	-202.5	1427.34	0.58	2445.2	1832.77	TRUE	FALSE
**Q14565**	DMC1	-0.32	-38.62	-91.39	14.16	0.05	907.33	945.95	TRUE	FALSE
**Q8TC99**	FNDC8	-0.41	-21.18	-44.41	2.05	0.07	420.41	441.59	TRUE	FALSE
**O14832**	PAHX	-0.7	-315.3	-516.52	-114.07	0	2300.2	2615.5	TRUE	TRUE
**Q9UFW8**	CGBP1	-0.1	-3.07	-16.39	10.25	0.01	99.31	102.38	FALSE	FALSE
**Q96GK7**	FAH2A	-0.29	-205.02	-530.45	120.4	0.3	1354.9	1559.92	TRUE	FALSE
**Q8N7B1**	HORM2	-0.33	-21.76	-52.14	8.62	0.03	222.15	243.91	FALSE	FALSE
**P57768**	Sorting nexin 16	-0.21	-31	-95.96	33.97	0.58	560.39	591.38	TRUE	FALSE
**Q9UL42**	PNMA2	-0.33	-81.86	-193.27	29.54	0.05	1399.64	1481.5	TRUE	FALSE
**Q8TAM6**	ERMIN	-0.1	-6.97	-36.35	22.42	0.03	359.61	366.58	FALSE	FALSE
**P55042**	RAD	-0.08	-19.81	-141.99	102.37	0.07	368.65	388.46	TRUE	FALSE
**Q9BZL3**	SMIM3	0.38	105.18	-12.12	222.48	0.11	947.87	842.68	TRUE	FALSE
**Q9UBU3**	Obestatin	0.54	126.27	25.79	226.75	0.04	1114.36	988.09	TRUE	FALSE
**Q9UBU3**	ghrelin	0.54	126.27	25.79	226.75	0.04	1114.36	988.09	TRUE	FALSE
**Q96AT1**	K1143	-0.27	-44.42	-121.03	32.19	0.43	1066.4	1110.82	TRUE	FALSE
**Q5VUM1**	CF057	-0.27	-26.21	-69.92	17.5	0.02	344.58	370.78	FALSE	FALSE
**Q8NDH3**	PEPL1	-0.33	-78.25	-192.18	35.68	0.09	308.27	386.52	TRUE	FALSE
**Q9NZN4**	EHD2	0.4	1038.02	-102.29	2178.33	0.05	11215.13	10177.11	TRUE	FALSE
**Q9UJ83**	HACL1	-0.28	-84.03	-228.58	60.52	0.27	657.75	741.78	TRUE	FALSE
**Q9BST9**	RTKN	-0.66	-67.93	-115.38	-20.48	0.01	545.36	613.29	TRUE	FALSE
**Q14833**	GRM4	0.29	1427.5	-604.45	3459.44	0.2	2057.4	629.9	TRUE	FALSE
**P35914**	HMGCL	0.26	557.45	-371.64	1486.53	0.26	3592.25	3034.81	TRUE	FALSE
**Q8N5L8**	RP25L	0.33	163.16	-43.44	369.77	0.06	935.22	772.06	TRUE	FALSE
**Q6NUJ5**	PWP2B	0.35	28.39	-7.54	64.33	0.04	736.53	708.14	TRUE	FALSE
**Q5VW32**	BROX	0.12	31.57	-79.41	142.56	0.29	1176.91	1145.34	TRUE	FALSE
**Q8N4X5**	AF1L2	0.46	155.46	12.38	298.53	0.19	758.18	602.72	TRUE	FALSE
**P53701**	CCHL	0.21	38.36	-39.69	116.4	0.03	486.46	448.1	FALSE	FALSE
**Q9Y6X0**	SETBP	0.34	39.04	-9.26	87.35	0.52	543.13	504.09	TRUE	FALSE
**Q6QNY0**	BL1S3	0.44	78.49	3.42	153.57	0.32	941.73	863.24	TRUE	FALSE
**P57055**	DSCR6	0.43	537.23	11.29	1063.16	0.07	1513.17	975.95	TRUE	FALSE
**P21549**	SPYA	0.11	72.35	-238.57	383.28	0.04	623.06	550.7	FALSE	FALSE

**Q5UCC4**	INM02	-0.45	-31.22	-61.3	-1.14	0.11	443.61	474.83	TRUE	FALSE
**Q9H3N1**	TXND1	0.06	49.77	-313.04	412.59	0.03	1401.17	1351.4	TRUE	FALSE
**O15266**	SHOX	-0.4	-48.3	-106.4	9.8	0.05	229.85	278.15	TRUE	FALSE
**Q4G0F5**	VP26B	-0.24	-58.04	-173.27	57.19	0.05	366.91	424.95	FALSE	FALSE
**Q86WN1**	FCSD1	-0.01	-4.15	-242.34	234.04	0.03	1548.08	1552.23	FALSE	FALSE
**P51178**	Phospholipase C-delta-1	0.04	24.15	-277.7	326	0.23	2337.75	2313.6	TRUE	FALSE
**P35712**	SOX6	-0.48	-52.22	-103.01	-1.44	0	368.4	420.62	TRUE	FALSE
**Q7Z5H3**	RHG22	-0.57	-12.53	-22.52	-2.55	0.01	199.97	212.5	TRUE	FALSE
**P48382**	RFX5	0.24	48.45	-36.07	132.97	0.45	860.25	811.81	TRUE	FALSE
**Q9ULA0**	DNPEP	-0.37	-51.93	-113.74	9.89	0.05	796.84	848.77	TRUE	FALSE
**O60609**	GFRa-3	0.24	47.92	-38.58	134.43	0.4	999.58	951.65	TRUE	FALSE
**P29144**	TPP2	-0.29	-356.18	-906.68	194.32	0.48	1958.11	2314.29	TRUE	FALSE
**Q8IUI8**	CRLF3	-0.34	-46.32	-108.64	15.99	0.02	493.53	539.86	TRUE	FALSE
**Q86WJ1**	CHD1L	0.41	47.64	-1.63	96.91	0.15	495.46	447.82	TRUE	FALSE
**Q13263**	KRIP-1	0.24	1036.29	-760.4	2832.98	0.05	3593.73	2557.44	FALSE	FALSE
**Q15124**	PGM5	0.5	782.05	104.75	1459.34	0.03	5358.78	4576.73	TRUE	FALSE
**Q9UBZ4**	APEX2	0.3	32.26	-13.29	77.82	0.32	540.17	507.91	TRUE	FALSE
**Q5TDH0**	DDI2	-0.62	-3210.25	-5582.4	-838.09	0.02	10664.55	13874.8	TRUE	FALSE
**Q8TD55**	PKHO2	-0.16	-227.87	-862.34	406.6	0.65	3028.07	3255.94	TRUE	FALSE
**O60784**	TOM1	-0.54	-27.89	-51.69	-4.09	0.03	283.11	311	TRUE	FALSE
**P05231**	IL-6	-0.12	-4.61	-22.39	13.16	0.32	534.12	456.82	TRUE	FALSE
**O14678**	ABCD4	0.1	95.75	-290.89	482.4	0.05	703.01	607.26	TRUE	FALSE
**Q92608**	DOCK2	-0.52	-58.42	-108.17	-8.68	0.02	1522.92	1581.35	TRUE	FALSE
**Q05066**	SRY	-0.33	-24.46	-58.15	9.23	0.24	464.16	488.62	TRUE	FALSE
**P21860**	ERBB3	-0.44	-301.97	-604.25	0.31	0.08	4172.89	4474.86	TRUE	FALSE
**Q16288**	TrkC	0.2	155.82	-181.64	493.29	0.23	3872.15	3716.32	TRUE	FALSE
**P06732**	CK-MM	-0.34	-111.27	-261.31	38.77	0.11	654.49	765.76	TRUE	FALSE
**P14210**	HGF	0.37	226.46	-32.44	485.37	0.17	1527.04	1300.58	TRUE	FALSE
**Q16674**	MIA	-0.39	-171.89	-366.92	23.14	0.04	2078.81	2250.7	TRUE	FALSE
**P14555**	NPS-PLA2	0.43	640.34	22.59	1258.09	0.05	2074.55	1434.21	TRUE	FALSE
**Q12904**	EMAP-2	-0.47	-204.82	-398.87	-10.77	0.09	2329.31	2534.13	TRUE	FALSE
**P24821**	Tenascin	0.17	41.74	-61.59	145.07	0.01	1450.11	1258.41	TRUE	FALSE
**O75173**	ADAMTS-4	0.08	1.18	-5.24	7.6	0.64	167.78	166.6	TRUE	FALSE
**O00253**	ART	-0.44	-76.02	-153.8	1.76	0.12	993.5	1069.52	TRUE	FALSE
**O43278**	HAI-1	-0.48	-613.8	-1182.28	-45.33	0.03	4441.3	5055.11	TRUE	FALSE
**Q9Y5K2**	Kallikrein 4	0.37	18.74	-3.4	40.87	0.04	348.13	329.4	TRUE	FALSE
**Q9ULZ9**	MMP-17	0.21	49.18	-52.51	150.88	0.63	1045.22	996.03	TRUE	FALSE
**O43291**	SPINT2	-0.21	-30.32	-94.89	34.25	0.35	678.88	709.2	TRUE	FALSE

**P55008**	AIF1	-0.4	-1166.44	-2490.78	157.91	0.16	10591.8	11758.23	TRUE	FALSE
**P55773**	MPIF-1	-0.16	-61.19	-234.94	112.55	0.36	30971.43	33614.24	TRUE	FALSE
**P55773**	Ck-b-8-1	-0.16	-61.19	-234.94	112.55	0.36	30971.43	33614.24	TRUE	FALSE
**Q9UNE0**	EDAR	-0.35	-94.75	-215.95	26.44	0.2	816.65	911.4	TRUE	FALSE
**Q9H2X3**	DC-SIGNR	0.52	179.23	27.93	330.54	0.04	3660.73	3481.5	TRUE	FALSE
**P21246**	PTN	0.29	5359.42	-2907.71	13626.55	0.08	37226.78	31867.36	TRUE	FALSE
**P04275**	vWF	0.5	4103.29	561.87	7644.7	0.02	26800.33	22697.04	TRUE	FALSE
**P07858**	Cathepsin B	0.14	47.86	-99.56	195.28	0.09	2132.19	1895.98	TRUE	FALSE
**O95998**	IL-18 BPa	0.18	192.87	-268.72	654.46	0.72	4386.69	4193.82	TRUE	FALSE
**P00742**	Coagulation Factor Xa	-0.12	-89.37	-415.16	236.42	0.42	5580.98	5674.66	TRUE	FALSE
**P00742**	Coagulation Factor X	-0.12	-89.37	-415.16	236.42	0.42	5580.98	5674.66	TRUE	FALSE
**Q9HAV5**	XEDAR	0.45	174.07	13.34	334.81	0.04	864.2	690.13	TRUE	FALSE
**P35475**	IDUA	0.37	148.24	-25.33	321.81	0.09	1460.31	1312.07	TRUE	FALSE
**Q9NQ76**	MEPE	-0.15	-8.54	-34.28	17.2	0.04	152.17	160.71	FALSE	FALSE
**Q9NR71**	ASAH2	-0.26	-151.55	-414.16	111.05	0.33	1676.57	1828.13	TRUE	FALSE
**Q14563**	Semaphorin 3A	-0.39	-30.27	-65.25	4.71	0.12	658.41	688.68	TRUE	FALSE
**P00736**	C1r	-0.47	-10337.57	-20129.59	-545.56	0.02	109867.32	120204.89	TRUE	FALSE
**P0CG37**	CFC1	0.11	35.97	-102.68	174.62	0.43	1347.58	1311.61	TRUE	FALSE
**O75015**	FCG3B	0.42	405.22	-15.73	826.17	0.03	3558.29	3153.07	TRUE	FALSE
**P56159**	GFRa-1	0.48	106.39	12.27	200.51	0.05	947.49	841.1	TRUE	FALSE
**Q16270**	IGFBP-7	0.32	1686.58	-570.84	3944	0.46	33284.76	31598.18	TRUE	FALSE
**Q13007**	IL24	0.3	18.89	-8.52	46.3	0.23	432.56	413.67	TRUE	FALSE
**Q6UXM1**	LRIG3	-0.59	-764.64	-1336.3	-192.99	0.03	6837.18	7601.82	TRUE	FALSE
**P13497**	BMP-1	-0.32	-925.04	-2235.45	385.36	0.28	10893.33	11818.37	TRUE	FALSE
**Q9BU40**	CRDL1	0.45	305.23	10.85	599.61	0.05	2262.78	1957.56	TRUE	FALSE
**P20718**	Granzyme H	0.29	33.12	-14.5	80.73	0.36	396.94	363.82	TRUE	FALSE
**P49862**	Kallikrein 7	-0.57	-223.12	-407.34	-38.9	0.01	878.21	1101.33	TRUE	FALSE
**O94907**	1,00 DKK	0.22	703.71	-626	2033.42	0.84	7286.53	6582.82	TRUE	FALSE
**P01009**	a1-Antitrypsin	0.37	3190.83	-594.11	6975.77	0.04	36040.56	32849.73	TRUE	FALSE
**P15586**	GNS	0.03	16.07	-242.89	275.03	0.19	1177.8	1030.8	TRUE	FALSE
**Q99538**	LGMN	0.27	74.04	-45.39	193.47	0.12	2642.93	2209.72	TRUE	FALSE
**P35968**	VEGF sR2	-0.69	-573.76	-944.28	-203.25	0	5359.31	5933.08	TRUE	TRUE
**P43489**	TNR4	0.52	118.79	23.12	214.46	0.03	772.97	654.19	TRUE	FALSE
**Q13443**	ADAM 9	0.34	389.34	-91.31	869.98	0.05	1425.67	1036.34	TRUE	FALSE
**Q16790**	Carbonic anhydrase 9	0.22	20.71	-19.51	60.94	0.41	437.64	416.93	TRUE	FALSE
**P51665**	PSD7	0.25	19.25	-13.8	52.29	0.23	640.71	621.46	TRUE	FALSE

**P16109**	P-Selectin	0.31	2020	-827.75	4867.76	0.31	25979.66	23959.66	TRUE	FALSE
**P00749**	uPA	0.32	150.74	-55.11	356.59	0.15	2129.42	1978.68	TRUE	FALSE
**Q9HCB6**	Spondin-1	0.43	93.31	-1.44	188.07	0.05	1167.51	1074.19	TRUE	FALSE
**P05362**	sICAM-1	0.59	379.86	96.57	663.14	0.02	2035.01	1655.15	TRUE	FALSE
**Q8IZJ0**	IFN-lambda 2	0.38	20.48	-2.87	43.83	0.08	350.73	330.24	TRUE	FALSE
**Q9GZX3**	CHST6	0.28	17.11	-9.27	43.5	0.83	431.29	414.17	TRUE	FALSE
**O75356**	ENTP5	-0.35	-311.37	-695.9	73.16	0.12	4457	4768.37	TRUE	FALSE
**Q08188**	TGM3	-0.35	-401.9	-911.98	108.19	0.03	592.54	994.44	TRUE	FALSE
**Q4KMG0**	CDON	-0.61	-796.01	-1366.81	-225.21	0.01	5992.99	6789	TRUE	FALSE
**P10909**	Clusterin	-0.4	-746.54	-1572.26	79.17	0.09	17241.82	17988.36	TRUE	FALSE
**P18509**	PACAP-38	0.04	5.89	-56.51	68.28	0.05	184.17	147.21	TRUE	FALSE
**P18509**	PACA	0.04	5.89	-56.51	68.28	0.05	184.17	147.21	TRUE	FALSE
**P18509**	PACAP-27	0.04	5.89	-56.51	68.28	0.05	184.17	147.21	TRUE	FALSE
**P31937**	3HIDH	-0.26	-1681.23	-4830.57	1468.11	0.68	572.65	2253.88	TRUE	FALSE
**P01374**	TNF-b	0.26	120.51	-69.59	310.62	0.02	197.6	213.18	FALSE	FALSE
**P23284**	PPIB	0.1	23.29	-76.83	123.41	0.93	1839.4	1816.11	TRUE	FALSE
**P31947**	STRATIFIN	0.39	689.61	-46.13	1425.35	0.14	4736.84	4047.23	TRUE	FALSE
**P29317**	Epithelial cell kinase	0.33	78.35	-24.01	180.7	0.12	1123.69	1045.34	TRUE	FALSE
**Q13873**	BMP RII	0.25	83.2	-52.34	218.75	0.42	606.14	522.93	TRUE	FALSE
**P52823**	Stanniocalcin-1	0.28	860.13	-395.53	2115.78	0.02	2484.32	1624.19	FALSE	FALSE
**Q07021**	C1QBP	-0.28	-48.52	-124.19	27.16	0.08	2379.86	2428.37	TRUE	FALSE
**P28799**	GRN	0.46	1104.1	37.96	2170.25	0.04	13597.42	12493.32	TRUE	FALSE
**P04196**	HRG	-0.71	-998.13	-1619.11	-377.15	0	5709.37	6707.5	TRUE	TRUE
**Q08380**	LG3BP	0.52	1731.32	275.54	3187.1	0.01	9705.94	7974.62	TRUE	FALSE
**P00568**	Myokinase, human	-0.33	-2677.37	-6362.17	1007.44	0.24	21165.08	23842.45	TRUE	FALSE
**P16333**	NCK1	-0.47	-114.53	-231.13	2.07	0.14	640.83	755.36	TRUE	FALSE
**P30566**	PUR8	-0.5	-1675.91	-3172.05	-179.77	0.01	4972.42	6648.33	TRUE	FALSE
**Q15762**	CD226	0.46	86.5	7.33	165.66	0.2	1353.59	1267.09	TRUE	FALSE
**P16471**	Prolactin Receptor	0.13	31.11	-78.86	141.08	0.03	1014.15	1065.53	TRUE	FALSE
**P04035**	HMGR	0.34	39.4	-8.34	87.14	0.03	311.98	272.58	TRUE	FALSE
**P20393**	NR1D1	0.46	25.71	1.73	49.7	0.03	522.83	497.12	TRUE	FALSE
**P45985**	MP2K4	-0.42	-3005.63	-6136.26	124.99	0.07	22088.4	25094.04	TRUE	FALSE
**O00408**	cGMP-stimulated PDE	0.4	21.55	-1.71	44.81	0.08	466.48	444.93	TRUE	FALSE
**Q14432**	PDE3A	0.31	70.25	-23.82	164.31	0.26	545.85	475.6	TRUE	FALSE
**P45379**	Troponin T	-0.27	-115.19	-317.45	87.07	0.81	1064.95	1180.14	TRUE	FALSE
**P05783**	Keratin 18	-0.32	-30.96	-76.96	15.04	0.29	302.04	333	TRUE	FALSE

**P11309**	PIM1	0.23	36.74	-31.99	105.47	0.41	1146.02	1109.28	TRUE	FALSE
**Q01105**	SET	-0.49	-1011.52	-1940.08	-82.96	0.04	12451.12	13462.63	TRUE	FALSE
**P26842**	CD27	-0.46	-481.99	-975.14	11.15	0	3123.69	3605.68	TRUE	FALSE
**P01160**	ANP	0.24	59.04	-47.87	165.96	0.36	1242.87	1183.82	TRUE	FALSE
**Q13740**	ALCAM	0.25	416.15	-302.09	1134.39	0.41	10458.24	10042.09	TRUE	FALSE
**O60243**	H6ST1	-0.3	-43.4	-108.22	21.42	0.26	1004.1	1047.51	TRUE	FALSE
**P61247**	RS3A	0.34	25.61	-6.43	57.65	0.48	551.43	525.82	TRUE	FALSE
**O14763**	TRAIL R2:ECD	0.37	26.45	-3.84	56.73	0.26	474.21	447.77	TRUE	FALSE
**O14763**	TRAIL R2	0.37	26.45	-3.84	56.73	0.26	474.21	447.77	TRUE	FALSE
**O14763**	TRAIL R2:Death	0.37	26.45	-3.84	56.73	0.26	474.21	447.77	TRUE	FALSE
**Q9H772**	GREM2	0.35	310.25	-76.33	696.83	0.18	4795.06	4484.81	TRUE	FALSE
**Q8NBM8**	PCYXL	0.38	110.59	-17.9	239.09	0.03	2009.28	1898.69	FALSE	FALSE
**Q5GAN6**	RNS10	0.1	30.03	-96.82	156.88	0.35	772.07	742.04	TRUE	FALSE
**Q00888**	PSG4	0.47	69.96	6.89	133.04	0.13	525.97	456	TRUE	FALSE
**A4D1T9**	PRS37	0.14	9.24	-18.67	37.16	0.2	172.04	162.8	TRUE	FALSE
**P05160**	coagulation factor XIII B	-0.29	-1020.41	-2618.6	577.78	0.13	26493.11	27513.52	TRUE	FALSE
**Q9NPH6**	OBP2B	-0.16	-80.27	-301.58	141.04	0.03	784.99	865.26	FALSE	FALSE
**Q9NTU7**	CBLN4	-0.59	-263.61	-460.77	-66.46	0.01	1601.49	1865.11	TRUE	FALSE
**Q99784**	NOE1	0.31	73.42	-24.89	171.73	0.05	256.33	182.91	FALSE	FALSE
**P51124**	Granzyme M	0.48	57.24	7.88	106.6	0.01	498.39	441.15	TRUE	FALSE
**Q6UWP8**	SBSN	0.11	56.8	-157	270.61	0.02	479.46	422.66	FALSE	FALSE
**Q5T2D2**	TRML2	-0.36	-316.92	-706.13	72.3	0.03	3121.35	3438.26	FALSE	FALSE
**Q9BUN1**	CA056	-0.37	-170.52	-375.44	34.4	0.2	1429.6	1600.12	TRUE	FALSE
**P13385**	Cripto	-0.49	-470.32	-898.65	-41.99	0.01	900.34	1370.66	TRUE	FALSE
**P42702**	LIF sR	-0.38	-114.44	-249.15	20.27	0.14	1709	1823.44	TRUE	FALSE
**Q13561**	Dynactin subunit 2	-0.69	-97.5	-159.64	-35.36	0	1165.15	1262.65	TRUE	TRUE
**P30042**	ES1	-0.35	-8.7	-19.66	2.26	0.03	161.49	170.19	TRUE	FALSE
**O95157**	NXPH3	-0.53	-155.94	-287.08	-24.79	0.02	1881.94	2037.88	TRUE	FALSE
**Q9NZK5**	CECR1	0.44	2644.46	18.69	5270.24	0.06	14803.19	12158.73	TRUE	FALSE
**Q9UNI1**	ELA1	0.35	2837.43	-704.36	6379.22	0.05	27987.31	25149.88	TRUE	FALSE
**Q9UQ74**	PSG8	0.36	50.25	-7.6	108.1	0.08	501.51	451.26	TRUE	FALSE
**Q9BXJ1**	C1QT1	0.46	469.98	38.94	901.02	0.06	4817.57	4347.59	TRUE	FALSE
**Q9Y5H3**	PCDGA	0.15	40.27	-86.77	167.32	0.02	1012.43	972.15	FALSE	FALSE
**Q92874**	DNSL2	0.54	246.61	50.19	443.02	0.04	2625.94	2379.33	TRUE	FALSE
**Q6UXI9**	Nephronectin	0.28	57.63	-31.5	146.75	0.39	1028.15	970.53	TRUE	FALSE
**Q86SI9**	CEI	-0.42	-679.64	-1381.42	22.14	0.07	4880.61	5560.25	TRUE	FALSE
**O43897**	TLL1	-0.01	-26.08	-1861.66	1809.5	0.02	7594.64	7620.72	FALSE	FALSE
**P22792**	CPN2	-0.21	-800.08	-2492.84	892.67	0.16	20455.95	21256.04	TRUE	FALSE

**Q00889**	PSG6	0	0.25	-24.55	25.05	0.45	623.06	622.81	TRUE	FALSE
**Q6DKI7**	PVRIG	-0.18	-28.35	-96.74	40.05	0.28	1471.62	1499.97	TRUE	FALSE
**Q9UJZ1**	Stomatin-like protein 2	0.13	56.61	-118.93	232.16	0.02	942.23	885.62	FALSE	FALSE
**Q5TAT6**	CODA1	-0.52	-137.3	-255.79	-18.81	0.05	1190.25	1327.55	TRUE	FALSE
**P23327**	SRCH	0.35	60.57	-11.78	132.92	0.7	235.02	174.45	TRUE	FALSE
**Q14435**	GALT3	-0.11	-21.05	-111.32	69.22	0.33	531.85	552.9	TRUE	FALSE
**Q6DN72**	FCRL6	-0.32	-105.96	-262.4	50.47	0.23	1400.03	1505.99	TRUE	FALSE
**P10696**	PPBN	-0.12	-53.76	-260.73	153.21	0.82	324.45	335.08	TRUE	FALSE
**P02786**	TR:CD	0.13	83.61	-194	361.21	0.21	4502.05	3997.54	TRUE	FALSE
**P02786**	TR:ECD	0.13	83.61	-194	361.21	0.21	4502.05	3997.54	TRUE	FALSE
**O94766**	B3GA3	-0.23	-874.67	-2694.12	944.77	0.76	442.79	1317.46	TRUE	FALSE
**Q6UWJ8**	C16L2	-0.49	-49.67	-96.58	-2.75	0.07	344.75	394.42	TRUE	FALSE
**Q8NBK3**	SUMF1	-0.49	-145.23	-278.12	-12.34	0.06	1361.18	1506.41	TRUE	FALSE
**Q92562**	FIG4	0.18	760.31	-997.67	2518.29	0.02	1587.89	827.58	FALSE	FALSE
**Q9BXS0**	COPA1	-0.26	-6.32	-17.42	4.77	0.61	166.27	172.59	TRUE	FALSE
**P58658**	F176C:CD	0.45	2722.47	78.43	5366.51	0.02	24416.47	21694	TRUE	FALSE
**P58658**	F176C:ECD	0.45	2722.47	78.43	5366.51	0.02	24416.47	21694	TRUE	FALSE
**P21854**	CD72	0.03	5.05	-76.06	86.16	0.53	1057.77	920.34	TRUE	FALSE
**O75023**	LIRB5	0.04	165.63	-1799.97	2131.23	0.84	11940.92	11775.29	TRUE	FALSE
**O75326**	Semaphorin-7A	0.22	163.72	-150.58	478.02	0.87	1818.13	1654.42	TRUE	FALSE
**Q8N807**	PDILT	0.36	23.36	-4.59	51.3	0.35	623.57	600.21	TRUE	FALSE
**Q8N475**	FSTL5	-0.33	-15.92	-37.17	5.34	0.04	400.24	416.16	TRUE	FALSE
**Q9NTK1**	DEPP	-0.71	-938.68	-1531.05	-346.3	0	3202.58	4141.25	TRUE	TRUE
**Q6UXH0**	TD26	0.46	38.72	3.66	73.79	0.09	489.28	450.56	TRUE	FALSE
**P14415**	AT1B2	-0.5	-511.71	-972.57	-50.85	0.03	3530.35	4042.06	TRUE	FALSE
**Q99584**	S100A13	0.61	870.87	244.01	1497.73	0	3487.22	2616.35	TRUE	FALSE
**A6NI73**	LIRA5	0.6	218.53	64.54	372.52	0.02	1879.53	1661	TRUE	FALSE
**Q8N109**	KI2LA	0.16	49.25	-80.89	179.39	0.02	879.01	505.36	TRUE	FALSE
**Q9UQ72**	PSG11	-0.36	-33.42	-74.91	8.08	0.24	729.02	762.44	TRUE	FALSE
**Q24JP5**	T132A	0.19	86.96	-107.63	281.55	0.66	1809.47	1722.51	TRUE	FALSE
**Q8N5Y8**	PAR16	0.47	95.79	11.64	179.95	0.83	569.9	474.11	TRUE	FALSE
**Q9Y2R0**	CCD56	0.25	124.07	-83.59	331.73	0.64	2234.11	2110.04	TRUE	FALSE
**P19224**	UGT 1A6	0.27	322.29	-186.97	831.55	0.7	1119.9	797.61	TRUE	FALSE
**Q8TC05**	MDM1	0.47	54.39	4.53	104.24	0.04	986.45	932.07	TRUE	FALSE
**Q9BVH7**	SIA7E	0.31	31.59	-11.1	74.28	0.64	376.41	344.82	TRUE	FALSE
**O60507**	TPST1	-0.32	-303.61	-723.66	116.43	0.18	3983.47	4287.09	TRUE	FALSE
**Q8NBV8**	SYT8	0.3	166.41	-62.13	394.94	0.71	384.72	218.31	TRUE	FALSE
**O95461**	LARGE	0.35	85.84	-20.17	191.86	0.12	1055.12	969.27	TRUE	FALSE

**Q86UP6**	CUZD1	-0.5	-94.91	-185.49	-4.32	0.03	284.02	378.93	TRUE	FALSE
**P19827**	ITI heavy chain H1	-0.66	-3764.09	-6264.88	-1263.29	0	39492.08	43256.16	TRUE	TRUE
**Q9BYC8**	RM32	0.2	23.57	-27.34	74.47	0.03	442.46	418.89	TRUE	FALSE
**Q9H3S3**	Spinesin	0.24	19.91	-15.48	55.3	0.93	421.5	401.59	TRUE	FALSE
**P84157**	MXRA7	0.34	97.69	-22.52	217.91	0.12	999.4	901.71	TRUE	FALSE
**Q96CU9**	FXRD1	-0.48	-79.59	-154.89	-4.29	0.07	682.84	762.43	TRUE	FALSE
**P15151**	Poliovirus receptor	-0.14	-193.54	-850.28	463.2	0.64	752.08	945.62	TRUE	FALSE
**P0C8F1**	PATE4	0.23	12.56	-9.95	35.08	0.9	223.94	211.38	TRUE	FALSE
**Q9Y6H6**	KCNE3	0.26	33.44	-19.44	86.31	0.7	289.18	255.74	TRUE	FALSE
**Q1L6U9**	PSMP	-0.59	-213.03	-377.4	-48.66	0.03	952.51	1165.54	TRUE	FALSE
**O15400**	Syntaxin-7	0.34	2327.04	-670.9	5324.98	0.25	15529.57	13202.53	TRUE	FALSE
**O00300**	OPG	0.44	933.25	0.05	1866.46	0.04	7666.62	6733.37	TRUE	FALSE
**Q6IA17**	SIGIRR	0.21	44.58	-48.43	137.58	0.1	856.05	811.48	TRUE	FALSE
**A8MWY0**	K132L	0.38	17.63	-2.11	37.38	0.13	415.83	398.2	TRUE	FALSE
**Q86XK7**	VSIG1	0.42	31.84	0.36	63.32	0.02	205.84	174	TRUE	FALSE
**P08138**	NGF R	-0.4	-1068.71	-2358.4	220.99	0.03	1302.46	2371.17	TRUE	FALSE
**P01229**	LSHB	0.42	44.6	-0.49	89.69	0.07	476.2	431.6	TRUE	FALSE
**Q9UBX1**	CATF	0.29	11.94	-6.41	30.28	0.26	1660.99	1468.89	TRUE	FALSE
**Q2I0M5**	RSPO4	0.3	47.97	-19.73	115.67	0.09	445.77	397.8	TRUE	FALSE
**Q12805**	FBLN3	0.47	301.32	30.36	572.28	0.02	2749.39	2448.07	TRUE	FALSE
**Q8NCW6**	GLT11	0.25	33.47	-23.94	90.88	0.37	1291.97	1258.49	TRUE	FALSE
**P18206**	Vinculin	0.49	178.75	24.42	333.09	0.01	794.55	615.8	TRUE	FALSE
**Q8N5W8**	FA24B	0.33	30.18	-8.5	68.85	0.76	420.58	390.4	TRUE	FALSE
**Q9P2E7**	PCD10:CD	-0.03	-51.66	-764.85	661.52	0.15	1536.5	1373.54	TRUE	FALSE
**Q9P2E7**	PCD10:ECD	-0.03	-51.66	-764.85	661.52	0.15	1536.5	1373.54	TRUE	FALSE
**Q9P218**	COKA1	-0.49	-11.49	-22.77	-0.2	0.21	70.4	81.88	TRUE	FALSE
**P60059**	SC61G	0.27	15.07	-10.21	40.35	0.04	399.25	384.17	TRUE	FALSE
**Q86U42**	PABP2	0.22	84.5	-78.14	247.13	0.45	1206.11	1121.62	TRUE	FALSE
**O75354**	ENTP6	-0.53	-40.07	-74.18	-5.95	0.02	839.91	879.98	TRUE	FALSE
**Q6UVK1**	CSPG4	-0.5	-57.9	-112.07	-3.73	0.01	502.88	560.78	TRUE	FALSE
**Q8IVH8**	M4K3	0.28	10.02	-5.54	25.58	0.15	338.38	328.36	TRUE	FALSE
**Q6Q8B3**	MO2R2	0.42	72.96	1.04	144.87	0.35	480.37	407.41	TRUE	FALSE
**P54709**	AT1B3	0.07	486.26	-2377.76	3350.28	0.04	8197.06	7710.81	FALSE	FALSE
**O94906**	PRP6:region 1	-0.06	-4.59	-34.83	25.64	0.04	726.93	808.72	TRUE	FALSE
**O94906**	PRP6:region 2	-0.06	-4.59	-34.83	25.64	0.04	726.93	808.72	TRUE	FALSE
**Q96D42**	TIM-1	0.29	587.97	-247.87	1423.81	0.09	1657.52	1069.55	TRUE	FALSE
**Q8NFZ4**	NLGN2:CD	-0.18	-48.37	-180.77	84.03	0.04	246.29	262.07	FALSE	FALSE

**Q8NFZ4**	NLGN2:ECD	-0.18	-48.37	-180.77	84.03	0.04	246.29	262.07	FALSE	FALSE
**P52943**	CRIP2	0.42	121.5	-3.55	246.55	0.11	1633.88	1512.38	TRUE	FALSE
**Q5XG99**	LYSM4	0.3	38.6	-14.29	91.5	0.45	314.49	275.88	TRUE	FALSE
**Q5DID0**	UROL1	0.38	29.32	-2.75	61.39	0.47	459.61	430.29	TRUE	FALSE
**P04155**	TFF1	0.37	513.42	-75.11	1101.95	0.23	2868.51	2355.09	TRUE	FALSE
**P60604**	UB2G2	-0.47	-108.58	-211.45	-5.7	0.04	1573.82	1682.4	TRUE	FALSE
**P50897**	PPT1	0.33	68.75	-22.19	159.69	0.33	999.7	930.95	TRUE	FALSE
**P16562**	CRIS2	-0.74	-381.2	-619.63	-142.77	0	1056.16	1437.36	TRUE	TRUE
**Q8TAE8**	G45IP	-0.16	-4.98	-18.31	8.35	0.49	273.43	278.41	TRUE	FALSE
**O60487**	MPZL2	-0.43	-112.81	-230.77	5.14	0.06	1110.68	1223.5	TRUE	FALSE
**Q96HG1**	CX069	0.26	46.76	-29.12	122.65	0.52	731.54	684.78	TRUE	FALSE
**O95156**	NXPH2	-0.25	-86.23	-236.73	64.26	0.23	828.55	914.79	TRUE	FALSE
**O00453**	LST1	0.4	231.29	-14.34	476.93	0.12	2195.55	1964.26	TRUE	FALSE
**Q13753**	Laminin gamma-2	0.45	609.45	44.54	1174.37	0.03	2724.49	2115.03	TRUE	FALSE
**Q96PZ7**	CSMD1	-0.4	-62.78	-134.27	8.71	0.21	740.72	803.51	TRUE	FALSE
**P43246**	MSH2	-0.47	-176.95	-353.47	-0.43	0.07	577.61	754.56	TRUE	FALSE
**Q8TDY8**	IGDC4	-0.53	-92.75	-170.65	-14.85	0.1	708.94	801.69	TRUE	FALSE
**Q9UKW4**	VAV3	-0.31	-82.62	-199.23	33.99	0.11	1381.66	1464.28	TRUE	FALSE
**P47813**	IF1AX	0.28	34.1	-17.65	85.86	0.53	660.86	626.75	TRUE	FALSE
**P48775**	T23O	-0.36	-269.31	-593.75	55.13	0.1	3031.69	3300.99	TRUE	FALSE
**Q8TED9**	AF1L1	0.2	106.2	-112.73	325.12	0.33	309.19	202.99	TRUE	FALSE
**P55316**	FOXGB	-0.38	-17.89	-38.52	2.74	0.16	145.7	163.59	TRUE	FALSE
**O75829**	LECT1	-0.28	-82.94	-211.69	45.82	0.18	2161.76	2244.7	TRUE	FALSE
**P04264**	Keratin-1	0.54	295.52	64.8	526.24	0.1	1660.37	1364.85	TRUE	FALSE
**Q9UN67**	PCDBA	-0.56	-147.88	-269.36	-26.41	0.01	1171.73	1319.61	TRUE	FALSE
**Q8N3Z0**	PRS35	-0.33	-67.38	-165.37	30.6	0.04	501.12	568.51	TRUE	FALSE

Table S2. KEGG database pathway enrichment analysis for all the proteins associated to diagnosis (651 proteins). N Genes: Number of genes. FDR: False discovery rate. Fold enrichment is calculated based on the observed number of genes in the pathway divided by the expected number of genes in the pathway[1]. The enrichment FDR is calculated based on the Benjamini-Hochberg correction of the Fisher's exact test[1].

Enrichment FDR	N Genes	Pathway Genes	Fold Enrichment	Pathway	Genes
**2.51085581412774e-06**	27	294	3.64811862244898	Path:hsa04060 Cytokine-cytokine receptor interaction	EDAR IL24 IL17F IL17RE CSF1 GDF7 GDF10 IL<br>L2 IFNA4 IL5 IL6 IL6R TNFRSF9 INHBA LIFR<br>NFRSF11B IL22 PRLR EDA2R BMPR1B BMPR2<br>4 IL1R2 TNFRSF10B CD27
**0.000191745669841688**	26	354	2.9175788606403	Path:hsa04151 PI3K-Akt signaling pathway	CDK2 COL6A1 CSF1 CREB3L4 EFNA3 EGFR EP<br>BB3 VEGFD FLT4 HGF TNC IFNA4 IL6 IL6R IT<br>DR LAMC2 MTCP1 MYC NGFR PRLR SGK1 CR<br>SPP1 VWF
**0.000343437089581484**	18	200	3.57515625	Path:hsa04510 Focal adhesion	VAV3 COL6A1 EGFR ELK1 VEGFD FLT4 MYLP<br>TNC ITGA2 KDR LAMC2 MYL7 SPP1 BRAF VC<br>CAPN2
**0.00210273506143515**	15	170	3.50505514705882	Path:hsa04310 Wnt signaling pathway	FZD10 CSNK2A2 DKK1 DKK4 DKK2 RSPO1 RS<br>C SOST PPP3R1 LGR4 SFRP5 WNT5A RSPO3 L
**0.00242140654688771**	11	99	4.41377314814815	Path:hsa04640 Hematopoietic cell lineage	CR2 CSF1 IL5 IL6 IL6R ITGA2 TFRC IL1R2 CD<br>A CD9
**0.00267867379709375**	64	1538	1.6530125704378	Path:hsa01100 Metabolic pathways	COQ7 B3GALT5 HIBADH SAT2 CKM HYKK CO<br>P2 ADSL DGKB DLD AGXT AK1 MLYCD ALPI<br>GALNT3 AOC1 B3GAT3 GNS PGP GSTP1 HAG<br>HMGCL HMGCR MMAB IDUA ALDH6A1 NAG<br>1 PAFAH1B3 PDE2A PDE3A ATP5PB UPB1 AD<br>2G2A PLCD1 UGT1A6 PNPO PGM2 PPT1 KYAT<br>H2 KMT2C RPE GALNT11 BLVRB NMNAT1 BP<br>D CA9 ST6GALNAC5 POMK B3GALT2 CBR1 ST<br>IDI2 LARGE1 ENTPD6 ENTPD5 FIG4 XYLB
**0.0026922193720459**	20	294	2.70231009070295	Path:hsa04010 MAPK signaling pathway	CSF1 EFNA3 EGFR EPHA2 ELK1 ERBB3 VEGFD<br>HGF HSPB1 KDR MAP3K11 MYC NGFR PPP3R<br>K6 PTPN7 MAP2K4 BRAF MAP4K3
**0.00778122472561134**	9	84	4.25613839285714	Path:hsa04012 ErbB signaling pathway	EGFR ELK1 ERBB3 NRG1 MYC NCK1 MAP2K4<br>BTC
**0.00778122472561134**	13	166	3.11091239959839	Path:hsa04022 cGMP-PKG signaling pathway	CREB3L4 KNG1 ATP1B2 ATP1B3 NPPA NPPB N<br>TP2A3 PDE2A PDE3A PPP3R1 PRKG1 CREB3L2
**0.00778122472561134**	14	181	3.07257136279926	Path:hsa04360 Axon guidance	SEMA3A CFL2 GDF7 EFNA3 EPHA2 EPHA6 NC<br>3R1 SEMA4G BMPR1B SLIT3 BMPR2 WNT5A S
**0.00778122472561134**	9	85	4.20606617647059	Path:hsa04610 Complement and coagulation cascades	CLU CR2 F10 F13B KNG1 PLAU SERPING1 C1C<br>F
**0.00778122472561134**	10	103	3.85669498381877	Path:hsa04974 Protein digestion and absorption	COL6A1 COL10A1 COL13A1 CPA1 CPB1 ATP1B<br>B3 PRSS3 COL20A1 COL25A1
**0.0172001314510004**	8	79	4.02267932489451	Path:hsa01521 EGFR tyrosine kinase inhibitor resistance	EGFR ERBB3 HGF NRG1 IL6 IL6R KDR BRAF
**0.0172001314510004**	12	162	2.94251543209877	Path:hsa04630 JAK-STAT signaling pathway	IL24 EGFR IFNL2 IFNA4 IL5 IL6 IL6R LIFR MY<br>PIM1 PRLR
**0.0172001314510004**	9	97	3.6857280927835	Path:hsa05215 Prostate cancer	CDK2 CREB3L4 EGFR GSTP1 PLAU CREB3L2 S<br>RAF IL1R2
**0.0285766206178678**	14	221	2.51644984917044	Path:hsa04024 cAMP signaling pathway	VAV3 CGA ADCYAP1 CREB3L4 LHB ATP1B2 A<br>NPY NPPA ATP2A3 PDE3A GHRL CREB3L2 BR
**0.0377136593677669**	9	112	3.19210379464286	Path:hsa04668 TNF signaling pathway	CSF1 CREB3L4 VEGFD ICAM1 IL6 MAP2K6 MA<br>REB3L2 FADD
**0.0398716524197991**	23	476	1.91943496148459	Path:hsa05022 Pathways of neurodegeneration-multiple diseases	DCTN2 FZD10 CSF1 CSNK2A2 DKK1 DKK4 DK<br>KLC1 NEFL ATP2A3 ATP5PB PPP3R1 MAP2K6<br>BRAF STX1A UBE2G2 WNT5A CAPN2 FADD U<br>FIG4
**0.0416364262884972**	13	210	2.45910218253968	Path:hsa04015 Rap1 signaling pathway	VAV3 CSF1 EFNA3 EGFR EPHA2 VEGFD FLT4<br>DR NGFR MAP2K6 RAP1GAP BRAF
**0.0416364262884972**	11	162	2.6973058127572	Path:hsa05161 Hepatitis B	CDK2 CREB3L4 ELK1 IFNA4 IL6 MYC MAP2K6<br>K4 CREB3L2 BRAF FADD

Table S3. Transcription factors families significantly enriched ($p < 0.05$) according to TRANSFAC and JASPAR databases (only human origin were included). P-values are based on Fisher's exact test and adjusted p-values (q-values) are corrected according to the Benjamini-Hochberg procedure[2].

term	p-value	q-value
NFAT2	0.0001995321074739234	0.06365074228418156
PPARA	0.0009267238837761202	0.14781245946229116
LTF	0.004558679501037194	0.44898873042817905
SMAD4	0.005629952732641744	0.44898873042817905
SREBF2	0.01003370616595746	0.5342158353744388
RUNX1	0.01004794674685465	0.5342158353744388
MIB2	0.012734118011709579	0.580311949390765
CEBPB	0.015326688654753916	0.6111517101083124
ETV4	0.017695930432271197	0.6272224230993901
USF2	0.020521200144212406	0.6546262846003758
PDX1	0.028282582098918877	0.7659509485165976
RBPJ	0.03103722012684079	0.7659509485165976
POU3F1	0.031214301977165418	0.7659509485165976
SND1	0.0405410668359135	0.7875124811922151
FOS	0.04391921013798342	0.7875124811922151
CBEPB	0.044121681497102466	0.7875124811922151
JDP2	0.04757238342557294	0.7987152796188299

Supplementary references

1. Ge SX, Jung D, Yao R. ShinyGO: a graphical gene-set enrichment tool for animals and plants. Valencia A, ed. *Bioinformatics*. 2020;36(8):2628-2629. doi:10.1093/bioinformatics/btz931

2. Gene Set Knowledge Discovery with Enrichr - Xie - 2021 - Current Protocols - Wiley Online Library. Accessed July 10, 2024. https://currentprotocols.onlinelibrary.wiley.com/doi/10.1002/cpz1.90
